
JOURNAL INFORMATION
==============================
NLM Title Abbreviation: JBMR Plus
Journal ID: JBMR Plus
NLM Journal ID: 101707013
Journal ID: jbmrplus

JBMR Plus

EISSN: 2473-4039
Publisher: Oxford University Press

ARTICLE INFORMATION
==============================
PMCID: PMC13430967
DOI: 10.1093/jbmrpl/ziag126
Article ID: ziag126
Article version: 1
Subjects: Abstracts, AcademicSubjects/MED00010, AcademicSubjects/MED00160, AcademicSubjects/MED00250

Abstracts of the Bone Research Society Annual Meeting 2026

Publication date: 2026 Aug
Electronic publication date: 2026 Aug 3
Volume: 10
Issue: Suppl 3
First page: iii1
Last page: iii122
Copyright: © The Author(s) 2026. Published by Oxford University Press on behalf of the American Society for Bone and Mineral Research.
Copyright year: 2026
License: This is an Open Access article distributed under the terms of the Creative Commons Attribution License (https://creativecommons.org/licenses/by/4.0/), which permits unrestricted reuse, distribution, and reproduction in any medium, provided the original work is properly cited.
License URL: https://creativecommons.org/licenses/by/4.0/

Page count: 1

==============================

Bone Research Society
ANNUAL MEETING 2026
8-10 JULY 2026 MILE END LONDON, UK

### Book of Abstracts

### London, UK

### Wednesday 8 – Friday 10 July 2026

**Oral Communications**

**Printed Poster Abstracts**

### Overview of the BRS Annual Meeting 2026

The Bone Research Society (BRS), formerly the Bone and Tooth Society, was founded in 1950. The BRS is one of the largest national scientific societies in Europe dedicated to clinical and basic research into mineralised tissues and is the oldest such society in the world. Meetings are held annually, attracting a wide audience from throughout the UK and beyond. The presentations are traditionally balanced between clinical and laboratory studies. The participation of young scientists and clinicians is actively encouraged.

We were delighted to welcome over 160 delegates, faculty and sponsors to our in-person meeting in London, which was held at Queen Mary University of London, Mile End Campus from Wednesday 8 to Friday 10 July 2026.

This year we held three full days of conference with a full programme of invited speakers, oral communications, printed posters and four satellite symposia from our industry partners. We would like to thank our industry sponsors for their valuable support of our meeting.

On Wednesday 8 July we also held two workshops in parallel with the main programme; High Resolution Imaging in the Engineering Building and In Vitro Models of Bone in the Bancroft Building, both also on the Mile End Campus.

These were followed by the popular Early Career Researchers Workshop and networking session on the Wednesday evening at the Graduate Centre.

Topics covered in the main programme were:

- Osteoporosis Management
- Rare Bone Diseases
- Cellular Crosstalk in Bone and Cartilage Disease
- Primary Bone Cancers
- Old Bones, New Tricks
- Multi-Modal Analysis
- What's New in Muscoloskeletal Science

We were delighted to have excellent submissions from our authors which resulted in 14 being selected for Oral Communication presentations, 30 more for Quick Fire Poster Pitches and we had 75 printed posters on display.

***BRS 2026 Programme Committee***
*(serving July-December 2025)*

Dr Stefaan Verbruggen (Chair)
Professor Duncan Bassett
Professor Alan Boyde
Dr Judith Bubbear
Dr Moira Cheung
Professor Agi Grigoriadis
Dr Katie Moss
Professor Andy Pitsillides
Dr Isabel Orriss
Dr Scott Roberts
Dr Gudrun Stenbeck

***BRS 2026 Local Organising Committee at QMUL***
*(serving December 2025-July 2026)*

Dr Stefaan Verbruggen (Chair)
Dr Lucie Bourne
Professor Himadri Gupta
Professor Karin Hing
Professor Simon Rawlinson
Dr Clare Shere
Dr Hamish Simpson
Soamopriya Som
Professor Elizabeth Tanner
Georgina Wherry

### Oral Communications

### OC1.1

### The Effect of a One-Year Vitamin D and Calcium Supplementation on pQCT Bone Outcomes Among Adolescents Living with HIV and Established on Antiretroviral Therapy in Zimbabwe

Tafadzwa Madanhire[1,2,3], Mícheál Ó Breasail[1,4], Nyasha Dzavakwa[2,3], Victoria Simms[2,3], Katharina Kranzer[2,3], Cynthia Mukwasi-Kahari[2,3], Farirayi Kowo-Nyakoko[2,5], Rashida Ferrand[2,3], Kate Ward[5,6], Celia Gregson[1,2]

[1]University of Bristol, Bristol, UK. [2]The Health Research Unit, Biomedical Research and Training Institute, Harare, Zimbabwe. [3]London School of Hygiene and Tropical Medicine, London, UK. [4]Monash University, Clayton, Australia. [5]University of Southampton, Southampton, UK. [6]MRC Unit The Gambia at London School of Hygiene and Tropical Medicine, Banjul, Gambia

#### Abstract

*Background:*

HIV and delayed ART initiation both negatively affect linear growth and bone mineral accrual in adolescents living with HIV (ALWH). In low-resource settings deficits may be worsened by nutritional deficiencies.

*Objective:*

To determine the effect of 48 weeks of vitamin $D_3$ and calcium supplementation on peripheral quantitative computed tomography (pQCT) measured bone density, mass, and strength.

*Methods:*

A pre-planned secondary analysis of the Vitality trial was performed (PACTR20200989766029), a phase III individually randomized, double-blinded, placebo-controlled trial of vitamin $D_3$/calcium carbonate (20,000 IU weekly/500mg daily) in ALWH (age 11−19 yr) with tibial pQCT scans measuring: total vBMD, trabecular vBMD, cross-sectional area (CSA), bone strength index of compression (BSIc), cortical vBMD, bone mineral content (BMC) and thickness, and strength-strain index (SSI). Linear regression estimated mean between-arm differences in pQCT outcomes, and stratified by sex, pubertal stage, dietary calcium intake and 25(OH)D status at baseline.

*Results:*

422 ALWH (median age 16.2 years [IQR:13.6–18.1]) were equally randomised to intervention or control arms. At baseline, regardless of treatment arm, participants reported low daily dietary calcium intake, with a median of 107.5 mg/day [IQR: 70.1–146.8] and 64% (n=270/422) had 25(OH)D<75nmol/L. At 48 weeks, ALWH in the intervention arm showed greater gains in total vBMD [adjusted mean % difference (AMD):1.49, 95%CI; 0.40, 2.57], trabecular vBMD [AMD:1.44, 95%CI; 0.06, 2.95], and BSIc [AMD:2.51, 95%CI; 0.38, 4.63] compared with controls. No differences were observed for total CSA [AMD: –0.46, 95% CI; -1.93, 1.01]. Total vBMD gains were greater among females, those earlier in puberty, and with higher baseline dietary calcium intake. Cortical BMC and SSI improved most in ALWH with 25(OH)D<75nmol/L and lower than the median dietary calcium intake at baseline.

*Conclusion:*

Combined vitamin D and calcium supplementation is an effective, potentially scalable solution to improving bone mineral accrual in ALWH. Supplementation most benefits females, those at early puberty, and with pre-existing 25(OH)D insufficiency. The optimal duration of supplementation during bone accrual remains to be determined, as does the long-term effects of supplementation.

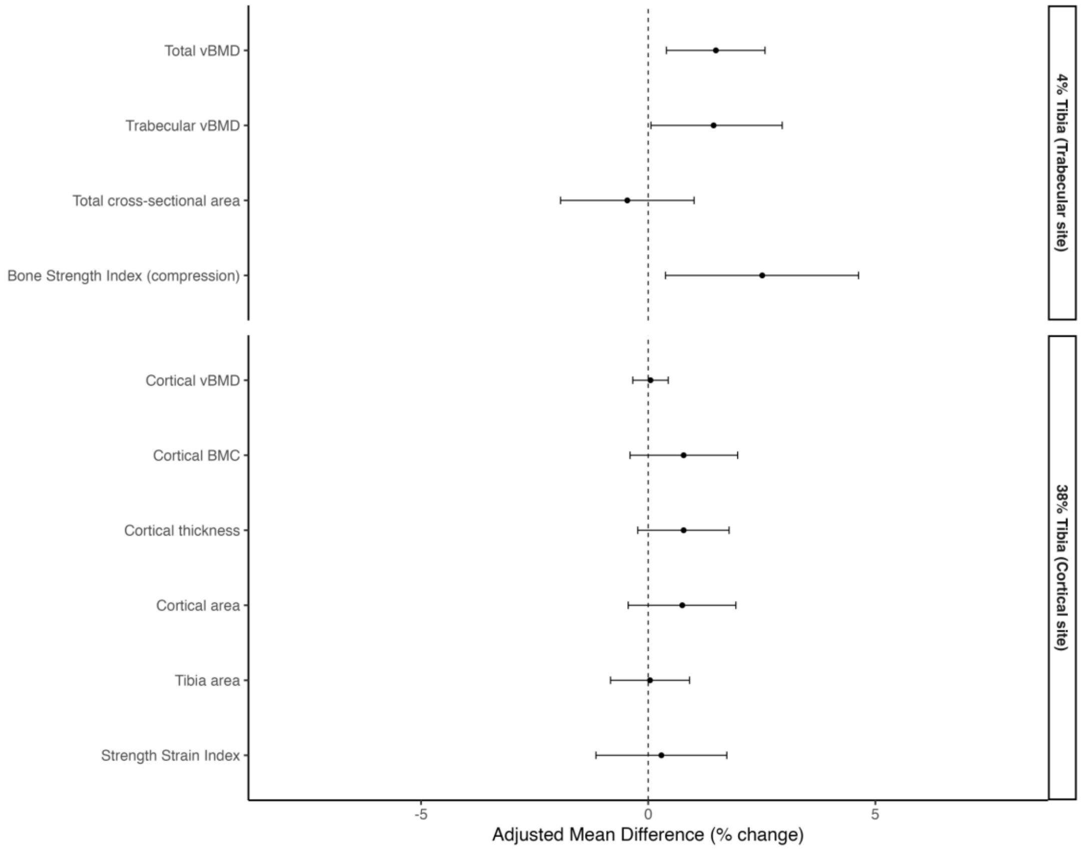

### OC1.2

### Palopegteriparatide Improves Skeletal Dynamics in Adults with Chronic Hypoparathyroidism: 214-Week Results from the Phase 2 PaTH Forward Trial

Jeremy Turner[1], Mishaela Rubin[2], Aliya Khan[3], Peter Schwarz[4], Andrea Palermo[5], Elena Tsourdi[6,7], Filomena Cetani[8], Lynn Kohlmeier[9], Rajesh Jain[10], Bart Clarke[11], Carol Zhao[12], Michael Ominsky[12], Bryant Lai[12], Jenny Ukena[12], Christopher Sibley[12], Aimee Shu[12], Lars Rejnmark[13]

[1]Norfolk and Norwich University Hospital, Norwich, UK. [2]Columbia University, New York, USA [3]McMaster University, Hamilton, Canada. [4]Rigshospitalet, Copenhagen, Denmark. [5]Fondazione Policlinico Campus Biomedico, Campus Bio-medico University, Rome, Italy. [6]Department of Medicine, Technische Universitat Dresden, Dresden, Germany. [7]Center for Healthy Aging, Technische Universitat Dresden, Dresden, Germany. [8]University of Pisa, Pisa, Italy. [9]Spokane Osteoporosis and Endocrinology - Arthritis Northwest Research, Spokane, USA. [10]University of Chicago, Chicago, USA. [11]Mayo Clinic, Rochester, USA. [12]Ascendis Pharma Inc, Palo Alto, USA. [13]Aarhus University Hospital, Aarhus, Denmark

### Abstract

Purpose:

Hypoparathyroidism is associated with low rates of bone remodeling and increased BMD due to insufficient levels of parathyroid hormone (PTH). Palopegteriparatide (TransCon PTH), a prodrug of PTH (1-34) administered once daily, is designed to provide active PTH within the physiological range for 24 hours/day in adults with chronic hypoparathyroidism. Palopegteriparatide has received regulatory approval in the US, EU, and several other countries. This analysis investigated skeletal endpoints through week 214 of the PaTH Forward trial.

Methods:

PaTH Forward was a phase 2, randomized, double-blind placebo-controlled trial (through Week 4) followed by an open-label extension period (through Week 266). BMD measured by DXA at the lumbar spine, total hip, femoral neck, and 1/3 distal radius, serum bone resorption marker CTx, and serum bone formation marker P1NP were all assessed at baseline and at regular intervals through week 214. Safety assessments included 24-hour urine calcium and treatment-emergent adverse events.

Results:

At baseline, participants (N=59) had a median (range) duration of hypoparathyroidism of 9 (1-39) years; mean (SD) age of 50 (12) years, and 81% were female. Of the 95% (N=56) of participants who continued to receive palopegteriparatide treatment through week 214, 93% were independent from conventional therapy (≤600 mg/day elemental calcium and no active vitamin D) and 98% had albumin-adjusted serum calcium levels in the normal range (8.3-10.6 mg/dL) with a mean (SD) of 8.94 (0.41) mg/dL.

As expected with hypoparathyroidism, mean CTx and P1NP were in the low end of normal at baseline, and increased with palopegteriparatide treatment, peaking at weeks 12 and 26, respectively. Thereafter, mean CTx and P1NP declined and remained stable and above baseline levels through week 214. The elevated baseline mean BMD Z-scores trended towards age- and sex-matched norms at the lumbar spine, femoral neck, and total hip and largely stabilized after 26 weeks of treatment, remaining above zero through week 214. Changes in BMD Z-scores were larger in participants with higher baseline values and longer duration of hypoparathyroidism. No new safety signals were identified.

Conclusions:

In adults with chronic hypoparathyroidism, continued treatment with PTH replacement therapy palopegteriparatide through week 214 of PaTH Forward showed sustained skeletal remodeling with trends toward a new skeletal steady state closer to age-appropriate norms.

Figure

Skeletal Responses to Palopegteriparatide

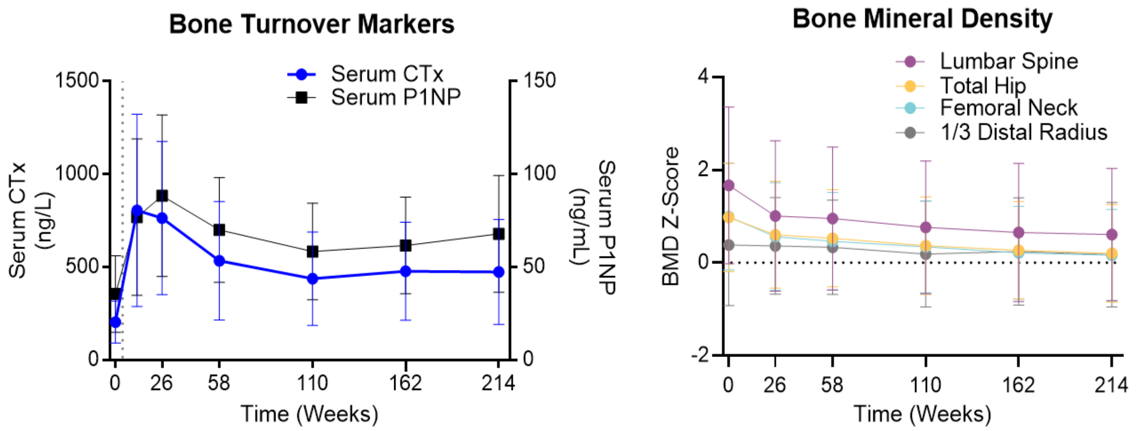

Means ± SD at each time point are plotted.

### OC2.1

### Navepegritide Combined with Lonapegsomatropin for the Treatment of Children with Achondroplasia: 52-Week Results from the Phase 2 COACH Trial

Melita Irving[1], Ciara McDonnell[2], Line Aagaard Nolting[3], Sohair G Abdelrahman[4], Lene Worsaae Dalby[5], Jennifer M Ikle[5], Simon Mejlhede Jensen[5], Allison S Komirenko[4], Michael Ominsky[4], Aimee Shu[4], Hanna Hove[3]

[1]Guy's and St. Thomas NHS Foundation Trust, Evelina Children's Hospital, London, UK. [2]Children's Health Ireland at Temple Street, Dublin, Ireland. [3]Copenhagen University Hospital, Rigshospitalet, Copenhagen, Denmark. [4]Ascendis Pharma Inc., Palo Alto, USA. [5]Ascendis Pharma A/S, Hellerup, Denmark

### Abstract

**Objective:** Navepegritide, a prodrug of C-type natriuretic peptide (CNP) designed to provide continuous exposure to active CNP, has been shown to significantly increase growth in children with achondroplasia (ACH) compared with placebo, while demonstrating a similar safety and tolerability profile. Navepegritide counterbalances the constitutively active fibroblast growth factor receptor 3 signaling in ACH via activation of the natriuretic peptide receptor B pathway to improve growth plate function. Adding once-weekly lonapegsomatropin, a prodrug of somatropin (human growth hormone [hGH]), to once-weekly navepegritide is hypothesized to augment effects on endochondral bone formation.

**Design and Methods:** In the open-label, phase 2 COACH Trial, children with ACH aged 2-11y received navepegritide (100 µg/kg/week) and lonapegsomatropin (starting at 0.30 mg hGH/kg/week). The trial had 2 cohorts: treatment-naive (TN) children, who began both treatments on Day 1 and navepegritide-experienced (NE) children, who added once-weekly lonapegsomatropin to weekly navepegritide monotherapy. The primary efficacy endpoint was the effect of navepegritide and lonapegsomatropin at Week 52 on annualized growth velocity (AGV) in the TN cohort versus navepegritide monotherapy in the ApproaCH Trial (NCT05598320) using an ANCOVA model with weights estimated by inverse probability treatment weighting.

**Results:** Twenty-one children were enrolled: 12 TN and 9 NE. In NE children, mean exposure to navepegritide was 2.56 years. At Week 52, TN children had a least squares mean AGV of 8.69 cm/year vs 5.95 cm/year for navepegritide alone in ApproaCH: difference=2.74 cm/year (95% CI, 2.11-3.38 $p<0.0001$). Statistically significant increases in linear growth from baseline to Week 52 occurred in TN and NE groups. TN children had a mean increase from baseline in AGV of +3.89 cm/year ($p=0.0015$) and of +1.02 in ACH-specific height Z-score ($p<0.0001$). NE children had a mean increase in AGV of +3.28 cm/year ($p<0.0001$) and of +0.86 in ACH-specific height Z-score ($p<0.0001$). At Week 52, AGV in both cohorts exceeded the 97th percentile for average stature children. Improvement in body proportionality was observed after 52 weeks of treatment and bone age remained consistent with chronological age. Arm span increased substantially (+9.4 cm in TN children; +7.9 cm in NE children), in line with gains in height. Navepegritide and lonapegsomatropin were associated with a safety and tolerability profile consistent with that observed for monotherapies.

**Conclusions:** One year of once-weekly navepegritide and lonapegsomatropin were well tolerated and augmented proportionate growth in children with ACH, suggesting that lonapegsomatropin, through a distinct mode of action, complements navepegritide's therapeutic effects in children with ACH.

### OC2.2

### New Skeletal Roles for the Dystrophin Glycoprotein Complex

Mark Hopkinson[1], Leah Wells[2], Scott Roberts[1], Andrew Pitsillides[1]

[1]Royal Veterinary College, London, UK. [2]Newcastle University, Newcastle, UK

### Abstract

The transmembrane dystrophin-glycoprotein complex (DGC) links the cell cytoskeleton to the extracellular matrix (ECM). Perturbation of this cell-ECM linkage due to mutations in components of the DGC or factors required for its functionally essential glycosylation cause muscular dystrophy (MD). MD patients suffer poor bone health and increased fracture risk which has historically been attributed to reduced muscle loading. Our phenotypic analysis of mutant murine MD models harbouring divergent DGC dysfunction, and in vitro studies have addressed the hypothesis that DGC:ECM interactions serve direct roles within the skeleton.

Micro-CT analysis in 3-week-old male mdx mice, a model of Duchenne MD, found longer tibiae and femora, with greater bone and tissue areas (versus WT) ($p<0.05$). In contrast, male mice harbouring FKRP mutations in which reduced α-dystroglycan glycosylation renders this core DGC component dysfunctional ($FKRP^{md}$), instead exhibited shorter bones with lower bone volume and mineral density (versus WT) ($p<0.05$), both at birth and adulthood before any loss in muscle force. These divergent long bone phenotypes were broadly echoed in the skulls, where mdx mice had larger, less dense skulls and $FKRP^{md}$ mice had reduced bone and tissue volumes (versus respective WT). Histological growth plate analysis revealed extended hypertrophic zones in both mdx and $FKRP^{md}$ mice ($p<0.01$ and $p<0.05$ respectively), with an extended proliferative zone only in mdx mice ($p<0.05$). Disruption of DGC:ECM interaction was further investigated in vitro by IIH6 antibody-mediated blockade of ATDC5 chondroprogenitor cell function in micromass, which found no effects of IIH6 on proteoglycan deposition (Alcian blue staining) but rather highly significant inhibition of mineralisation (Alizarin red staining $p<0.001$). Quantitative rt-PCR analysis (Sox9, Aggrecan, ColX, IBSP) of ATDC5 micromasses was consistent with a IIH6-mediated delay of hypertrophic maturation leading to reduced mineralisation. Moreover, microarray analysis of IIH6-treated ATDC5 cells revealed a transcriptional signature consistent with induction of anoikis, a form of apoptosis. Finally, generation of a tamoxifen-inducible cartilage-specific FKRP knockout mouse showed that selective FKRP loss led to smaller, weaker bones and changes in tibial trabecular and cortical bone akin to those seen in $FKRP^{md}$ mice.

Our data indicate: i) contrasting bone phenotype and growth plate behaviour in two divergent MD models, ii) direct in vitro effects of antibody-mediated blockade of DGC:ECM interaction in chondroprogenitor cell mineralisation, and iii) bone changes in cartilage-specific FKRP knockout mice. These findings suggest that the DGC serves direct skeletal roles that may contribute to phenotypes seen in MD patients.

A
Tissue Area
mm²
% Tibia Length
B
Bone Area
C
Bone Area/Tissue Area
%
D
WT
mdx
1mm
0%
100%
E
0%
100%
■ WT ■ mdx
3 mm
F
Density
> 500 um <

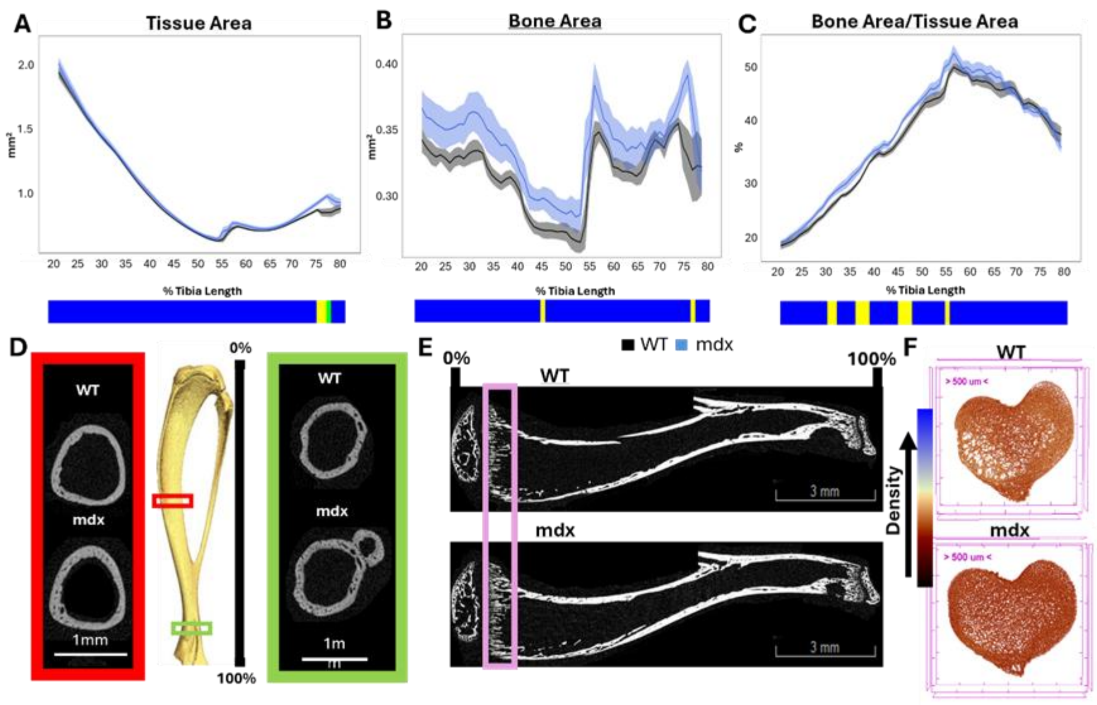

### OC3.1

### Screening Transcripomic and Enzymatic Modulators of Wnt Signalling to Drive Cartilage Homeostasis

Lydia Darby[1], Scott Roberts[2]

[1]Royal Veterinary College, London, UK. [2]Royal Vetinary College, London, UK

### Abstract

Osteoarthritis (OA) is a leading cause of disability globally; however, analgesia and joint replacement remain the only available treatments. In OA, chondrocytes become highly differentiated resulting in the loss of homeostasis and cartilage degeneration via hypertrophy and matrix mineralisation. Sclerostin antagonises canonical Wnt signaling through interaction with LRP4/5/6, and its downregulation in OA results in aberrant mineralisation through a process known as endochondral ossification. This study aimed to identify small molecule perturbants of gene expression for selected Wnt mediators, along with known inhibitors of Wnt pathway activation as a means to identify novel disease modifying OA drugs (DMOAD). It is hypothesised that transcriptomic modulation of canonical Wnt signalling will allow greater control over Wnt pathway activation. This is important as both cartilage-associated Wnt ablation and activation are known to result in OA, thus reinforcing the need to carefully modulate basal Wnt signalling or retain tight temporal control of inhibition. Transcriptomic perturbants were selected for their ability to downregulate *Lrp5*, or upregulate *Lrp4* or *Sost* using the Lincs 100 database of GEO (small molecules) from Harmonizome 3.0. To define efficacy, ADTC5 cells were cultured and plated in high-density micromasses in chondrogenic followed by mineralisation media to replicate the process of endochondral ossification. Selected compounds were initially screened at 1µM, and subsequently in a dose response. Micromasses were analysed using qPCR to determine gene expression; glycosaminoglycan deposition (Alcian Blue; AB) and matrix mineralisation (Alizarin Red; AR) was also quantified. Predicted perturbants showed variable effects across outcomes. Radicicol (RA) enhanced *Lrp4* (24h; 1µM: fold change (fc)=2.9, p<0.05), *Sox9* (24h; 1µM: fc=10.9, p<0.05) and *Acan* (24h; 1µM: fc=19.1, p<0.05) expression at early timepoints (24hrs), reduced mineralisation and increased glycosaminoglycan staining at day 14 (AR: fc=0.81, p<0.0001, AB: fc=1.5, p <0.01), suggesting maintenance of a healthy chondrocyte phenotype, whilst blocking pathological mineralisation. LGK974 (porcupine inhibitor that blocks Wnt secretion) increased expression of *Sox9, Sost* and *Acan* (14d; *Sox9:* fc=3.3, p<0.01, *Sost*: fc=4.7, p<0.05, *Acan:* fc=2.4 p<0.05) and significantly reduced terminal mineralisation (14d; AR; fc= 0.61, p<0.05). Collectively, these data indicate that RA and LGK974 block hypertrophic transition, and mechanistically this may be due to upregulation of the sclerostin anchor *Lrp4* and *Sost*, respectively. To test this hypothesis LGK and RA were co-dosed, resulting in a potent reduction in mineralised matrix suggestive of promising a synergistic effect (14d; AR; LGK974 0.01µM, RA 1µM fc=0.17, p<0.01). This study represents a novel approach to identify DMOADs that modulate pathological pathways via transcriptomic control of Wnt signalling.

### OC3.2

### Histological Study of the Association Between Hypertension and Structural Knee-Joint Changes

Makurata Zaninka, Sadaf Ashraf

Medway School of Pharmacy, Universities of Kent and Greenwich, Chatham, UK

### Abstract

**Background:** Ageing is associated with multi-morbidities leading to chronic pain, negatively impacting quality-of-life of individuals. Hypertension and osteoarthritis are common age-associated chronic pain conditions. People with osteoarthritis have a higher risk of developing hypertension [1] and individuals with hypertension are more likely to experience osteoarthritis [2, 3], however the exact mechanisms of their co-dependence are not known. Osteoarthritis can affect any joint, with knee-osteoarthritis accounting for 60%–85% of all reported cases [4]. Hypertension may increase intraosseous pressure (blood supply to the bone) leading to ischemia, thereby promoting vascular invasion at the osteochondral junction, contributing to bone remodelling [5, 6], however, detailed tissue analysis mapping the structural changes that occur in the knee following hypertension initiation have not been done.

The **aim of this study** was to map the peripheral changes in the knees of hypertensive and control rats to assess whether hypertension primes the knees to osteoarthritis-like changes.

**Methods:** Knees from spontaneously hypertensive (SHR) and normotensive Wistar-Kyoto (WKY) rats were assessed for structural and cellular changes using histology and immuno-histochemistry techniques. Changes within the synovium, cartilage and subchondral bone were quantified using Fiji-ImageJ and statistically analysed using GraphPad-prism. Data is presented as mean ± SEM of n=6 rats/group with a $p \leq 0.05$ as statistically significant.

**Results:** SHR rats had thicker trabecular bone (52.9±3.8%) and increased presence of multi-nucleated cells ($1.3 \times 10^{-4} \pm 7.9 \times 10^{-6}$ cells/$\mu m^2$) in the subchondral bone marrow compared with WKY rats (trabecular bone: 40.6±1.5%, $p<0.05$, multinucleated cells: $7.3 \times 10^{-5} \pm 2.1 \times 10^{-6}$ cells/$\mu m^2$, $p<0.01$). SHR rats had inflamed synovium, with higher total synovial cell count per $\mu m^2$ ($4.3 \times 10^{-3} \pm 0.2 \times 10^{-3}$), increased area covered by CD68$^{+ve}$ synovial macrophages (734±43.3$\mu m^2$) and increased number of small blood vessels (angiogenesis: 13.0±2.0), compared with WKY rats (cell count: $2.3 \times 10^{-3} \pm 0.4 \times 10^{-3}$ $p<0.01$, macrophages: 475±46.1$\mu m^2$, $p<0.05$, angiogenesis: 8.0±2.0 $p<0.05$). In addition, SHR rats had increased cartilage thickness, chondrocyte number and proteoglycan staining. These changes were mainly observed in the non-calcified cartilage.

**Conclusion:** Initial data suggests that hypertension primes the peripheral knee joints for osteoarthritis-like changes including synovitis, synovial angiogenesis, cartilage and subchondral bone remodelling. Understanding the shared mechanisms that drive these processes can lead to better disease control.

**References:**

1. Veronese N et al., (2018) Rejuvenation Res, 21:15-21
2. Lo K et al., (2022) J Orthop Translat, 32:12-20
3. Cruz CJ et al., (2025) Experimental Gerontology, 112938
4. Langworthy M et al., (2024) Ther Adv Musculoskeletal Dis, 16:1–22
5. Ching K et al., (2021) Nat Rev Rheum, 17:533-549
6. Yeater TD et al., (2023) Arthritis Res Ther, 25:7

### OC4.1

### Validation of STAT3 and MMP9 as Key Determinants of Osteosarcoma Formation and Metastasis

Wendy K Gomez Arenas[1], Daniela Paternina Martinez[2], Xuesong Wen[1], Scott J Roberts[2], Helen C Roberts[1]

[1]Middlesex University, London, UK. [2]Royal Veterinary College, London, UK

### Abstract

Osteosarcoma (OS), the most common primary malignant bone tumour in children and adolescents, is characterised by aggressive behaviour and frequent pulmonary metastasis. Survival is approximately 80% for localised disease but falls to 20–30% in metastatic cases. Advances in treatment have stagnated; consequently, survival rates have remained largely unchanged for decades, highlighting the need to better understand the mechanisms driving metastasis and to identify new therapeutic targets

To investigate factors regulating osteosarcoma migration and metastasis, our group previously developed a novel *in-vitro* metastasis Migratory Body (MB) assay using highly metastatic HOS-143B OS cells. MBs are spheroid-like structures characterised by enhanced stemness and the ability to generate secondary MBs, providing a functional readout of migratory potential. Using this model, signal transducer and activator of transcription 3 (STAT3) and matrix metalloproteinase 9 (MMP9) were identified as candidate regulators of metastatic behaviour and are interestingly associated with poor clinical outcomes in patients.

This allowed the formation of the hypothesis that inhibition of STAT3 and MMP9 would suppress metastatic potential. *In-vitro* small molecule-mediated inhibition of STAT3 and MMP9 significantly reduced MB formation in HOS-143B cells by 76% and 31%, respectively ($p \leq 0.001$). As proof of principle to establish anti-tumour activity, STAT3 inhibition was investigated using an optimised chorioallantoic membrane (CAM) xenograft model. This model enabled evaluation of STAT3 inhibitor treatment in HOS-143B tumours i*n-ovo*, providing a physiologically relevant system to complement *in-vitro* observations. Histological examination of excised tumours revealed a notable reduction in tumour neovascularisation and a trend towards reduced tumour cell integration (92.1%; $p = 0.07$) following STAT3 inhibitor treatment (Figure 1 A).

To further define the mechanistic roles of STAT3 and MMP9, CRISPR–Cas9 genome editing was used to generate isogenic STAT3 and MMP9 knockout (KO) clones in HOS-143B cells. Gene disruption was validated at genomic and transcript levels, with RT-qPCR demonstrating significant reductions in STAT3 and MMP9 expression of 92% ($p = 0.003$) and 81% ($p = 0.032$), respectively, relative to control cells. Functional analysis using the MB assay showed that loss of these genes significantly impaired migratory capacity, with STAT3 knockout reducing MB formation by 65.2% ($p = 0.0008$), while MMP9 knockout resulted in a 37.1% reduction ($p = 0.0035$) compared with controls (Figure 1 B and C).

Together, these findings show STAT3 and MMP9 act as key regulators of osteosarcoma tumour formation and cell migratory behaviour, validating them as drug targets to limit osteosarcoma progression.

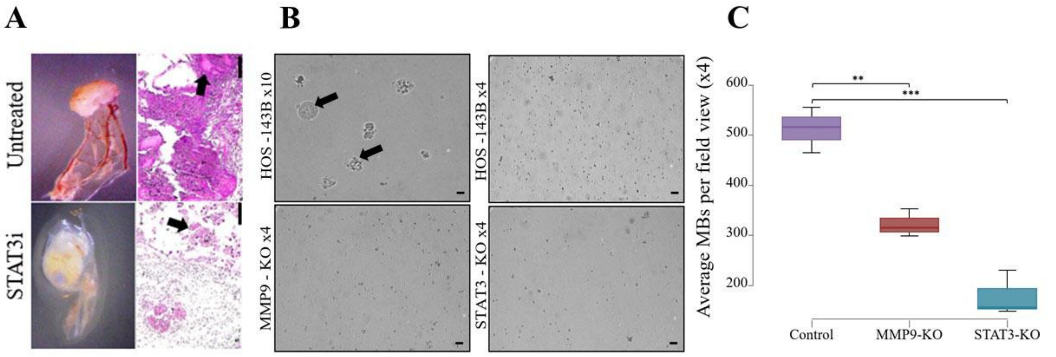

**Figure 1. STAT3 inhibition reduces vascularisation *in-ovo* and STAT3/MMP9 KO reduces migratory body (MB) formation in HOS-143B cells. (A)** CAM tumours derived from HOS-143B cells treated with a STAT3 inhibitor (STAT3i) or untreated control. Representative images of excised tumours and corresponding H&E-stained NuOss scaffold sections are shown. Arrows indicate blood vessels. Scale bar: 100 µm. **(B)** Representative phase-contrast images showing migratory body (MB) formation in parental HOS-143B cells compared with MMP9-KO and STAT3-KO cells. Arrows indicate MBs. Scale bars: 50 µm (4×) and 20 µm (10×). **(C)** Quantification of MB formation in parental HOS-143B, MMP9-KO and STAT3-KO cells at day 7. Data represent the average number of MBs per field of view (4×). Bars show mean ± SD (n = 3). $^{**}p < 0.01$; $^{***}p < 0.001$.

### OC4.2

### Investigating the Role of Alveolar Pneumocytes in the Lung Microenvironment of Osteosarcoma Metastases

Janice Pereira[1], Lauren McGee[1], Anjali Garg[1], Kate Silver[1], Troy McEachron[1], Christina Mazcko[1], Amy LeBlanc[1], Jessica Beck[1,2]

[1]National Cancer Institute, National Institutes of Health, Bethesda, USA. [2]The Ohio State University, Columbus, USA

### Abstract

Pulmonary metastasis is the leading cause of mortality in human and canine osteosarcoma (OS) patients. However, the contribution of lung-resident epithelial cells—particularly surfactant protein C (SPC) expressing type II alveolar pneumocytes (TIIPs)—to metastatic progression remains undefined. This study investigates the transcriptional as well as spatial changes in pneumocytes in OS lung metastases and explores their interactions with tumor cells.

Firstly, spatial transcriptomics was used to determine alveolar TIIPs' abundance and location in the tumor microenvironment of human OS lung matastases (n=7). Further, immunohistochemistry staining for SPC+ cells was carried out in both canine (n=14) and murine (n=9; orthotopic injection of K7M2 murine OS cell line) models of OS lung metastases. HALO quantitative image analysis was used to quantify SPC+ cells, and a paired *t*-test was performed for statistical analysis. To assess direct OS cell–pneumocyte crosstalk, human primary TIIPs were co-cultured with MG63.2 human OS cell line followed by RNA sequencing and cytokine analysis to identify gene expression changes and cytokine levels respectively. Furthermore, ELISA was used to detect and quantify functional protein levels.

Spatial transcriptomics of human patient tissues showed an accumulation of TIIPs in the region immediately adjacent to the tumor. Quantification of SPC+ cells in canine and murine tissue also demonstrated a significant increase of TIIPs within the tumor:non-tumor interface compared to non-tumor lung tissue. RNA sequencing, cytokine activity and ELISA demonstrated significant enrichment of the p53 pathway and elevated secretion of pro-fibrotic cytokines and TGFß1 in TIIPs co-cultured with OS cells versus TIIPs in monoculture. Together these findings suggests that there is overlap between the TIIP response in OS and idiopathic pulmonary fibrosis, a chronic lung disease driven by TIIPs.

This investigation highlights a previously under-recognized epithelial component of the OS metastatic microenvironment and suggests that pneumocytes play a role in the lung metastatic niche of OS. Future work aims to identify new approaches for targeting host–tumor interactions to limit metastatic progression of OS in both human and canine patients.

### OC4.3

Abstract Withdrawn

### OC5.1

### Bone Formation Without Osteocytes: Comparative Single-Nucleus Transcriptomics of Medaka and Zebrafish

Soher Jayash[1], Donald Dunbar[1], Sebastien Guizard[1], James Furniss[1], Andrew Pitsillides[2], Katherine Staines[3], Diego Robledo[1], Colin Farquharson[1], Louise Stephen[1]

[1]The Roslin Institute and Royal (Dick) School of Veterinary Studies, University of Edinburgh, Edinburgh, UK. [2]Comparative Biomedical Sciences, The Royal Veterinary College, Royal College Street, London, UK. [3]Centre for Lifelong Health, School of Applied Sciences, University of Brighton, Brighton, UK

**Abstract**

Bone adaptation to mechanical loading, and many other physiological roles, are widely attributed to osteocytes, which function as embedded mechanosensors within the mineralised matrix. Many teleost fish possess anosteocytic bone that lack osteocytes which, nonetheless, retains normal skeletal growth, maintenance, and (re)modelling. This raises a fundamental question: how is mechanical information detected and regulated in bone that lacks osteocytes? To address this, we compared vertebral bone from the anosteocytic teleost medaka fish species with that of the osteocytic teleost zebrafish. Single-nucleus RNA sequencing (snRNA-seq) was used to characterise cell populations and to pinpoint transcriptional differences specifically associated with the absence of osteocytes. Twelve distinct transcriptional cell type clusters were identified in both species. Only one cluster showed enrichment for genes associated with osteoblast and osteocyte function. Within this population, several osteoblast-related genes, including *col1a1a, col1a1b, runx2b,* and *sparc*, were more highly expressed in the anosteocytic medaka, whereas *sp7* and *spp1* were more strongly expressed in zebrafish. Genes commonly associated with osteocyte function, including *sost, irx5a and ngef* were detected in both species but were generally expressed in only a limited subset of nuclei. Functional enrichment analysis highlighted significant representation of genes involved in transient receptor potential (TRP) ion channel signalling in medaka. Consistent with this, *trpc5a* expression was significantly higher in medaka vertebrae ($p < 0.05$), as confirmed by qPCR. Spatial expression analysis using *in situ* hybridisation revealed that *sost* and *fgf23* were, as expected, localised primarily to osteocytes in zebrafish bone. In contrast, these archetypal osteocytic genes were restricted to osteoblasts lining the vertebrae surface in medaka. These data indicate that genes intimately linked to osteocytic delineation are expressed in the vertebrae of both species, suggesting that in anosteocytic fish, the functions attributed to osteocytes are transferred to another bone cell, most likely the osteoblast. This work therefore provides new insight into the evolutionary diversity of skeletal mechanotransduction mechanisms.

### OC5.2

### Uncovering Preclinical Endpoints in the *Sost*$^{-/-}$ Skull: Hearing Loss as a Novel Non-Invasive Readout

Timothy Dreyer[1], Eleanor Gouldie[1], Jacob Keen[1], Phil Salmon[2], Gill Holdsworth[3], Andrew Pitsillides[1], Scott Roberts[1]

[1]Royal Veterinary College, London, UK. [2]Bruker microCT, Kontich, Belgium. [3]UCB Pharma, Slough, UK

### Abstract

Hearing loss and potentially lethal raised intracranial pressure are key primary symptoms of sclerosteosis (OMIM #269500), an ultra-rare, autosomal recessive high bone mass (HBM) condition that predominantly manifests in skull pathologies.

Herein, we characterised skull parameters at later adult stages in a Sost-deficient (*Sost*$^{-/-}$*)* mouse model of sclerosteosis. Skulls of 9-month-old *Sost*$^{-/-}$ (n = 6 males and 4 females) and BL6 (n = 4 males and 4 females) mice were scanned by microCT (13.5 µm isotropic voxel), and bone morphometric parameters measured. Hearing response was assessed by exposing mice (4–9-month-old; n ≥ 4 for BL6 and *Sost*$^{-/-}$ age and sex groups) were exposed to high-frequency (~20 kHz) tone stimulus at 90 dB sound pressure level (dB SPL).

Increased bone mass was observed in the *Sost*$^{-/-}$ mouse skull and associated bony structures (male and female), thus replicating the predominant sclerosteosis-related pathologies (Fig. 1). Upon comparison of BL6 vs *Sost*$^{-/-}$ mice, *Sost*$^{-/-}$ mice had higher parietal/squamosal bone volume (BV; ~2-fold; $p \leq 0.0001$) in both sexes. Narrower foramen magna (foramen area lowered by >10%; $p \leq 0.0001$) were evident in both male and female *Sost*$^{-/-}$ mice. Otosclerosis, supported by greater ossicle BV (male: 32%; female: 18%; $p \leq 0.0001$; Fig. 1B), bone surface (BS; male: 38%; female: 27%; $p \leq 0.0001$), and otic capsule bone area (B.Ar; ~2-fold increase; $p \leq 0.0001$; Fig. 1C) were evident in the *Sost*$^{-/-}$ mouse middle and inner ear. Importantly, hearing response declined by 85% ($p \leq 0.0001$) and 34% ($p \leq 0.01$) in 8–9-month-old *Sost*$^{-/-}$ males and females, respectively. Increased mandibular BV (>65%; $p \leq 0.0001$) and BS (30%; $p \leq 0.0001$) highlighted *Sost*$^{-/-}$ mandibular hyperostosis.

Sexual dimorphism was evident in the *Sost*$^{-/-}$ mouse skulls, with higher parietal/squamosal bone mass (average bone thickness: 34%; $p \leq 0.001$; BV: 28%; $p \leq 0.0001$) and a narrower foramen magnum (>2% smaller area; $p \leq 0.01$) in *Sost*$^{-/-}$ females compared to males (Fig.1A). Females had higher otic capsule B.Ar (13%; $p \leq 0.01$; Fig. 1D), while, interestingly, males exhibited an ~300% ($p \leq 0.001$) higher degree of hearing loss. Furthermore, hyperostosis was also more prominent in female *Sost*$^{-/-}$ mandibles (BV: 13%; $p \leq 0.0001$), underscoring the importance of including both sexes in HBM studies.

This study provides new insight into *Sost*$^{-/-}$ skull pathologies and how these relate to functional outcomes, such as hearing. Importantly, hearing response may prove to be a valuable non-invasive preclinical measurement for sclerosteosis drug efficacy testing – a strategy which would also be in line with the 3R's principles.

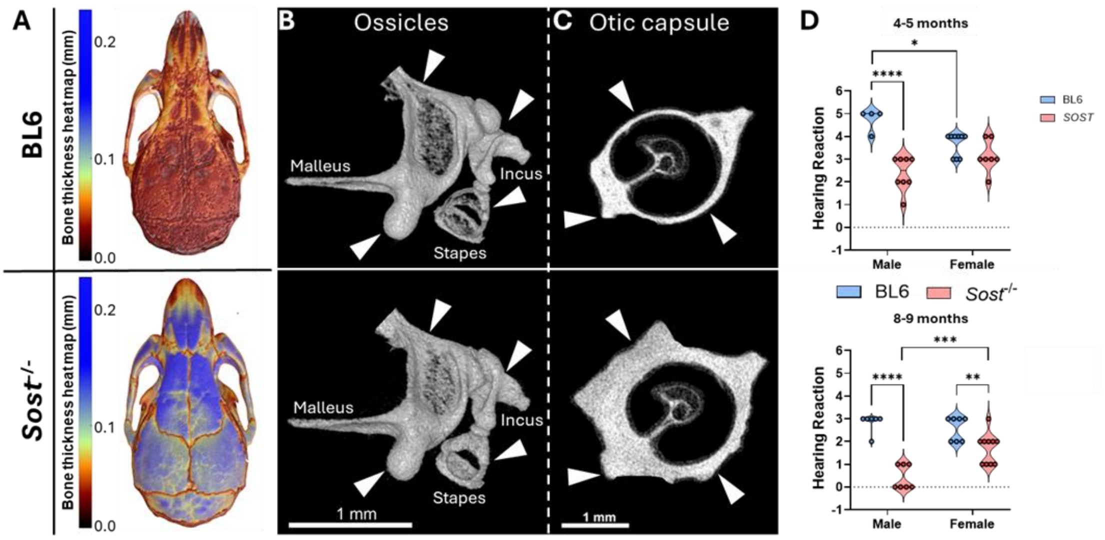

**Figure 1: Greater skull and middle/inner ear bone mass with declined hearing response in male and female *Sost*$^{-/-}$ mouse. A-C** Representative µCT images of different skull regions of 9-month-old, female BL6 (top) and *Sost*$^{-/-}$ (bottom) mice. **A** Colour coded (heat map of bone thickness) 3D µCT images of whole mouse skull. **B** 3D µCT images of middle ear ossicles. White arrows highlight otosclerosis in *Sost*$^{-/-}$ mouse ossicles. **C** Cross-sectional 2D µCT images of inner ear otic capsule. White arrows highlight hyperostosis in *Sost*$^{-/-}$ mice. **D** Hearing reaction in 4–5-month-old (top) and in 8–9-month-old (bottom) male and female BL6 and *Sost*$^{-/-}$ mice. Means indicated by solid line, and upper and lower dashed lines represent quartiles. Blue and red represent BL6 and *Sost*$^{-/-}$, respectively. $*P \leq 0.05$, $**P \leq 0.01$, $***P \leq 0.001$ and $****P \leq 0.0001$. For morphometric parameters, $n = 4$ BL6 males, $n = 4$ BL6 females, $n = 6$ *Sost*$^{-/-}$ males and $n = 4$ *Sost*$^{-/-}$ females. Hearing reaction BL6 and *Sost*$^{-/-}$ age and sex groups varied in size, with $n \geq 4$.

### OC6.1

### Hierarchical Phase-Contrast Tomography Enables Organ-to-Micron Scale Mapping of the Intact Human Spine

Aikta Sharma[1,2], Isla Henderson[1], Alissa Parmenter[1,2], Joseph Brunet[1], David Stansby[1], Christian Liebsch[3], Jishizhan Chen[1], Joanna Purzycka[4], Aurelien Delphin[5], Laurent Lamelle[5], Theresa Urban[4], Hector Dejea[6], Andrew Pitsillides[7], Hans-Joachim Wilke[3], Timothy Bray[1], Alexandre Bellier[5], Paul Tafforeau[4], Peter Lee[1,2]

[1]University College London, London, UK. [2]Research Complex at Harwell, Oxford, UK. [3]Ulm Medical Centre, Ulm, Germany. [4]European Synchrotron Radiation Facility, Grenoble, France. [5]Université Grenoble Alpes, Grenoble, France. [6]ANAXAM, Villigen, Switzerland. [7]Royal Veterinary College, London, UK

### Abstract

Spinal disorders, including osteoarthritis, osteoporosis and degenerative disc disease and axial spondyloarthritis, are major causes of chronic lower back pain and disability worldwide. Clinical diagnosis often relies on multimodal imaging, with radiography and computed tomography used for inspection of calcified structures while magnetic resonance imaging enables evaluation of intervertebral discs (IVDs), bone marrow, and surrounding soft tissues. However, none of these modalities have the spatial resolution required to resolve either the microarchitectural determinants of spinal integrity, or the early alterations underpinning disease initiation. Synchrotron-based hierarchical phase-contrast tomography (HiP-CT) uniquely addresses this limitation ex vivo by enabling non-destructive, micrometre-scale visualisation of calcified and non-calcified tissues within intact organ systems. Here, we apply HiP-CT to a complete adult human spine for the first time to explore whether this form of multiscale whole-organ imaging can resolve vertebral bone microarchitecture, endplate-disc interfaces and vascular and nervous tissues in three dimensions.

An intact adult human spine (C1-L5) from a 62-year-old male donor with no known history of spinal disease was dissected and chemically fixed in neutral buffered formalin and imaged using clinical 3 T MRI ($T_1$w-, $T_2$w-, $T_2$*w and $T_2$-w-STIR sequences) prior to HiP-CT. For HiP-CT, the spine was mounted in formalin-crushed agar and imaged on the BM18 beamline (16.26 µm/voxel) at the European Synchrotron Radiation Facility (MD1290)with selected regions of interest (chosen to correspond with areas of abnormality on MRI) imaged at 2.2 µm/voxel. Microarchitectural and geometric analyses were performed following tomographic reconstruction (Bruker, Belgium).

While MRI enabled gross spinal anatomy to be visualised, HiP-CT permitted resolution of fine tissue microstructure, including vertebral trabecular network organisation, endplates, IVD substructures, spinal vasculature and neural elements without exogenous contrast agents. Quantitative analysis of lumbar vertebrae (L1-L5) revealed regional variation in trabecular thickness, number and pattern factor corresponding to differences in vertebral trabecular eccentricity and polar moment of inertia along the cranio-caudal axis. These microarchitectural differences were observed at spinal levels demonstrating clinical features of IVD degeneration.

This study demonstrates the utility of HiP-CT for three-dimensional, multiscale ex vivo characterisation of the intact human spine. By exceeding the spatial resolution of clinical imaging while simultaneously resolving calcified and non-calcified tissues, HiP-CT enables direct visualisation of spinal microarchitecture. This approach provides a foundation for identifying new structural biomarkers of the earliest stages of spinal pathology in ex vivo tissues that are not detectable with routine imaging, without the need for exogenous contrast agents.

### OC6.2

### Multimodal Phenotypic Analysis of Cartilage:Bone Transition Reveals the Leptin-GAB2-Pi3K/mTOR Pathway as a Potential Mediator of OA

Olivia R A Caine, Lydia A Darby, Andrew A Pitsillides, Scott J Roberts

Skeletal Biology Group, Department of Comparative Biomedical Sciences, The Royal Veterinary College, London, UK

### Abstract

Osteoarthritis (OA) is the most common musculoskeletal condition, with cartilage:bone transition (Endochondral Ossification; EO) as a key event in joint destruction. Mutations in EO modulators manifest as short/long limb syndromes, such as achondroplasia due to *FGFR3* gain-of-function. Individuals with achondroplasia have characteristics that should increase OA risk (e.g. obesity, bowleg and joint hypermobility), however, OA is rarely seen in this population. Interestingly, FGF18 (Sprifermin; high-affinity FGFR3 ligand) is being clinically developed as a disease modifying OA drug and displays striking structural benefits in individuals with OA. This provides proof of principle regarding the identification of OA drug targets through an understanding of genes that underpin modified EO during skeletal development.

Herein, *in silico* discovery screening of the International Mouse Phenotyping Consortium (IMPC) for EO modulators (using tibial length, TL, as an output) revealed 220, both known and novel, genes that significantly modified TL ($p<0.0001$). Genes with likely osteoclast/osteoblast mediator function, which also cause alterations in TL, were removed through parallel assessment of whole-body BMD. This analysis identified 35 genes enriched in key regulators of osteoclast activity (e.g. *Ctsk*, *Lrrk1* and *Plekhm1*; Figure 1). Leptin (*Lep*) displayed greatest reduction in TL in both sexes (male fold change (FC): 0.82; female FC: 0.79; $p=3.14\times10^{-61}$). Furthermore, STRING network analysis of genes associated with decreased TL revealed *Gab2* to function as a novel node closely interconnected with *Lep* (Figure 1). Moreover, OAtarget/SkeletalVis analysis revealed *Gab2* expression to be lower in proliferative versus hypertrophic rat cartilage (FC: 0.22; $p=1.64\times10^{-14}$; *Col10a1* as hypertrophic marker FC=0.15; $p=2.60\times10^{-11}$). Intriguingly, this analysis also showed that whilst *Lep* expression was unaltered across cartilage zones, it exhibited raised levels in mesenchymal stromal cell (MSC)-derived osteoblasts vs MSCs (FC: 122; $p=9.9\times10^{-6}$), implying that *Lep* functions in a bone-to-cartilage paracrine fashion. To test this hypothesis, recombinant leptin (rLep, 50ng/ml) was added to ATDC5 cells in micromass undergoing EO *in vitro*, in the presence/absence of GDC-0980 (Pi3K/mTOR inhibitor, downstream of *Gab2,* 1µM;). This found that rLep induced a moderate trend to increased EO levels (mineralisation FC:1.24; p=0.22), which were rescued below baseline by GDC-0980 (FC:0.47; p=0.008) without affecting chondrogenesis (glycosaminoglycan content).

These data demonstrate a congruence between genes involved in cartilage:bone transition and functional outcomes using validated EO cell models. Clearly, further investigation of the *Lep-Gab2-Pi3k/mTor* signalling axis is warranted in the context of EO driven pathological processes in OA.

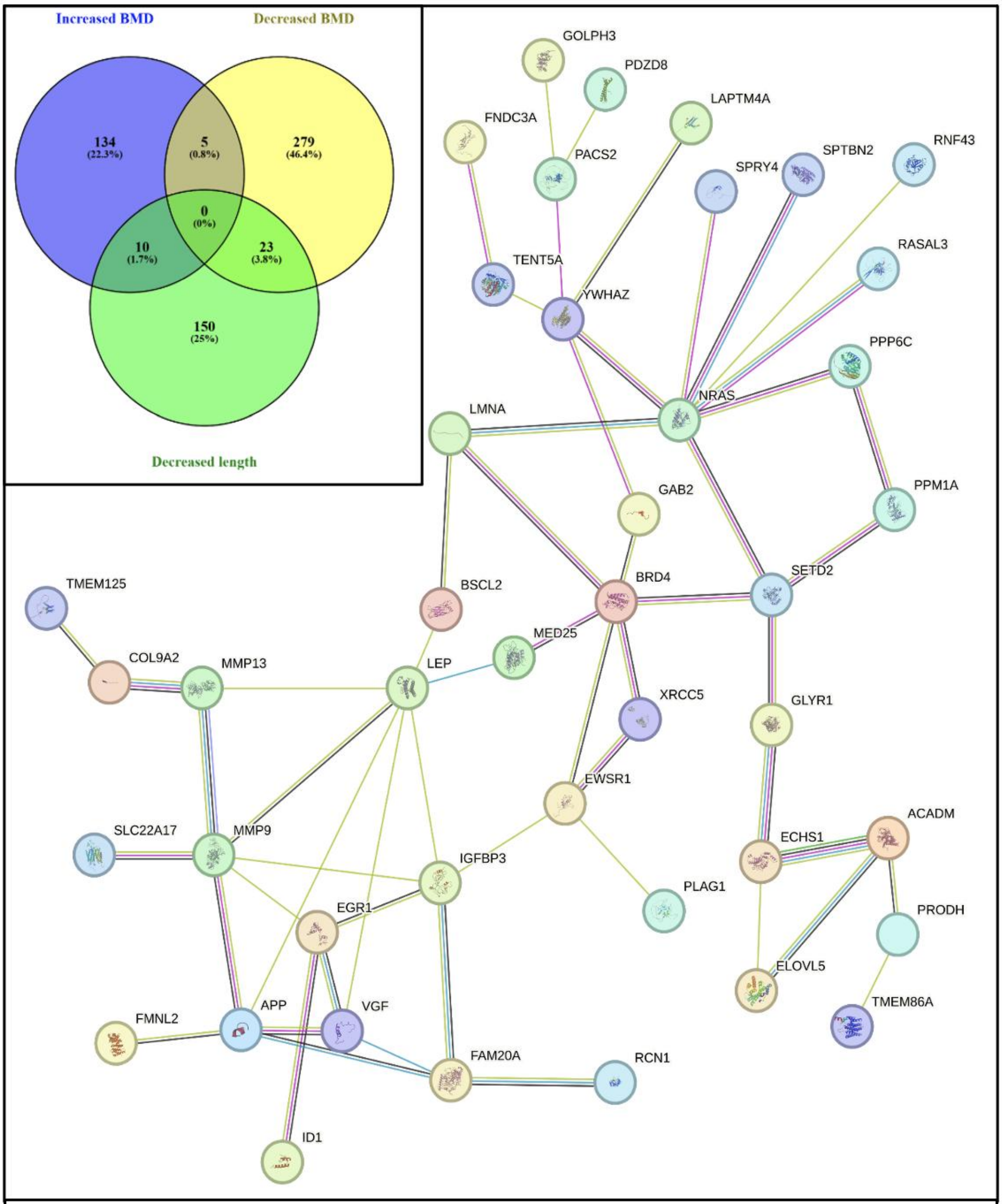

**Figure 1:** Genes involved in decreased tibial length (green) vs altered BMD (blue and yellow; inset) and associated interconnected STRING diagram of genes derived from IMPC in relation to short tibial length minus BMD modulators.

### OC7.1

### A Human iPSC-Derived Organotypic Bone Model Identifies Disease-Relevant Alterations in the Osteoblast / Osteocyte Lineage in Paget's Disease of Bone

Helen Knowles[1], Daniel Scott[2], Martina Marcotulli[1], Carla Cohen[1], Jennifer Brown[3], Antony Palmer[1], Muhammad Javaid[1], Mathew Baldwin[1], Sarah Snelling[1], Eleanor Stride[1,4], Dario Carugo[1], Philippa Hulley[1]

[1]NDORMS, University of Oxford, Oxford, UK. [2]University of Nottingham, Nottingham, UK. [3]Nuffield Orthopaedic Centre, Oxford, UK. [4]Institute of Biomedical Engineering, University of Oxford, Oxford, UK

### Abstract

Paget's Disease of Bone (PDB) is a rare metabolic bone disease displaying excessive osteoclastic bone resorption followed by pathological bone thickening, forming weakened bone with disordered collagen and hypomineralised patches. Germline mutations in sequestosome 1 (SQSTM1/p62), a multifunctional chaperone that coordinates proteostatic pathways including autophagy, occur in ~50% of cases.

Osteoclasts are key therapeutic targets in PDB but these short-lived cells are unlikely to be initiators of focal lesions. Osteocytes are the longest living bone cells. They form functional networks, sense mechanical load and control local bone resorption and formation. Mechanical sensitivity and osteocyte network connectivity are lost with age, suggesting that osteocytic networks may provide an initiation site for PDB lesions.

Exploring this hypothesis has been restrained by limitations of human osteocyte culture. We recently described development of a primary human bone cell organotypic model ('minibone') containing mature, sclerostin- and FGF23-producing osteocytes and osteoblasts within a mineralized matrix. We have now advanced this model, introducing human iPSC with a PDB-relevant null mutation in SQSTM1 (SQSTM1 KO) to model null mutations that cause severe PDB.

iPSC were seeded in fibrin/thrombin gels around 3D-printed scaffolds. Following cell-mediated contraction of the gel between the scaffold posts, osteogenic media was provided for <20 weeks. Both hBMSC- and iPSC-derived minibones secreted OPG and osteocytic markers sclerostin and FGF23, consistent with endocrine functions, and suppressed sclerostin secretion in response to ultrasound-based mechanical loading (21.7±10.9 pg/ml pre-ultrasound vs 3.4±4.8 pg/ml post-ultrasound, $p<0.001$).

Initial matrix contraction was stronger in SQSTM1 KO minibones (KO 55.2 ± 26.4% gel area vs isogenic control, $p<0.01$), suggesting altered cell-matrix interactions and/or loading response by SQSTM1 KO cells, which exhibit prominent F-actin stress fibres. SQSTM1 KO minibones also displayed increased mineral density at 20 weeks (KO 49.6±23.5% vs 15.4±10.7% isogenic control, $p<0.05$).

Secretome analysis of SQSTM1 KO minibones revealed reduced sclerostin secretion (18 weeks: KO 17.3±14.2 pg/ml vs 35.5±18.4 pg/ml isogenic control, $p<0.01$) and increased secretion of senescence (e.g. CXCL1-3, EGF, MCP3) and chronic inflammation-associated (e.g. TNFRI, BMP6) proteins, including the IL-6 receptor commonly upregulated in Pagetic tissue, versus isogenic controls. Importantly, this happens without osteoclasts, suggesting disease mechanisms beyond the traditional osteoclast focus.

This demonstrates that human iPSC-derived minibones containing mutations relevant to rare bone disease can detect differences in osteocyte outputs and effectively screen for osteocyte-targeting agents via effects on sclerostin secretion. They represent a valuable new tool for in vitro study of osteocyte-driven mechanisms of disease.

### OC7.2

### Characterising Zebrafish Osteocytes in Heath and Disease: A Foundation for Breakthrough Discoveries in Bone Biology

Beatriz Laraz-Prieto[1], Jiun Kai Ng[1], Ximena Hual[1], Faith Maes[2], Andrii Iakovliev[1], Martin Lee[1], Francesco Turci[3], P Eckhard Witten[2], Erika Kague[1]

[1]Institute of Genetic and Cancer, University of Edinblurgh, Edinburgh, UK. [2]Department of Biology, Ghent University, Ghent, Belgium. [3]School of Physics, Univeristy of Bristol, Bristol, UK

### Abstract

Osteocytes, the 'superstars' of bone biology, are now understood to be critical orchestrators of skeletal remodelling. Their complex, neuron-like networks, embedded within the bone matrix, govern mechanotransduction and intercellular communication, processes vital for bone health. Dysregulation of these networks underlies debilitating conditions like osteoporosis and osteoarthritis, impacting millions globally. While zebrafish has emerged as a premier vertebrate model for biomedical research, it lacks a comprehensive characterisation of zebrafish osteocytes, hindering our ability to leverage the powerful genetic tools of zebrafish to investigate fundamental osteocyte biology and translate findings to human health. Here we report the first characterisation of osteocytes during the lifespan of zebrafish, using advanced high-resolution imaging systems spanning from confocal microscopy, synchrotron micro-computed tomography, and customised Stimulated Raman Scattering (SRS) 3D confocal microscopy, and provide comparative gene expression profiling.

Our study shows the presence of functional osteocytes in zebrafish as early as one-month post-fertilisation. Strikingly, in adults, osteocyte distribution mirrors predicted mechanical loading, with sparse populations in low-load regions **(Figure 1).** Osteocyte orientation aligns perpendicularly to loading direction, and regions of active remodelling exhibit larger, less cylindrical osteocytes amidst woven bone. Consistent with mammalian models, zebrafish osteocytes form a dense, neuron-like network, displaying three distinct cell populations based on dendritic process complexity. We observed significant variations in dendrite process length, including extended, non-branching processes up to 30µm. Furthermore, aged zebrafish display osteocyte morphology consistent with mammalian aging, including rounded lacunae and micropetrosis. We demonstrate that Sp7, crucial for osteocyte dendritic process formation in mammals, functions similarly in zebrafish, as loss-of-function sp7 mutants exhibit rounded lacunae and abnormal osteocyte processes. Notably, wild-type zebrafish bones express a broad suite of mammalian osteocyte signature genes, with enrichment of differentially expressed genes involved in the TGFbeta pathway during ageing. Suggesting a high level of evolutionary conservation in osteocyte signalling in health and in disease.

Our detailed characterization establishes zebrafish as a robust model for studying osteocyte biology, revealing conserved architecture and functional responses, particularly in dendritic process regulation, thereby offering new avenues for investigating skeletal diseases.

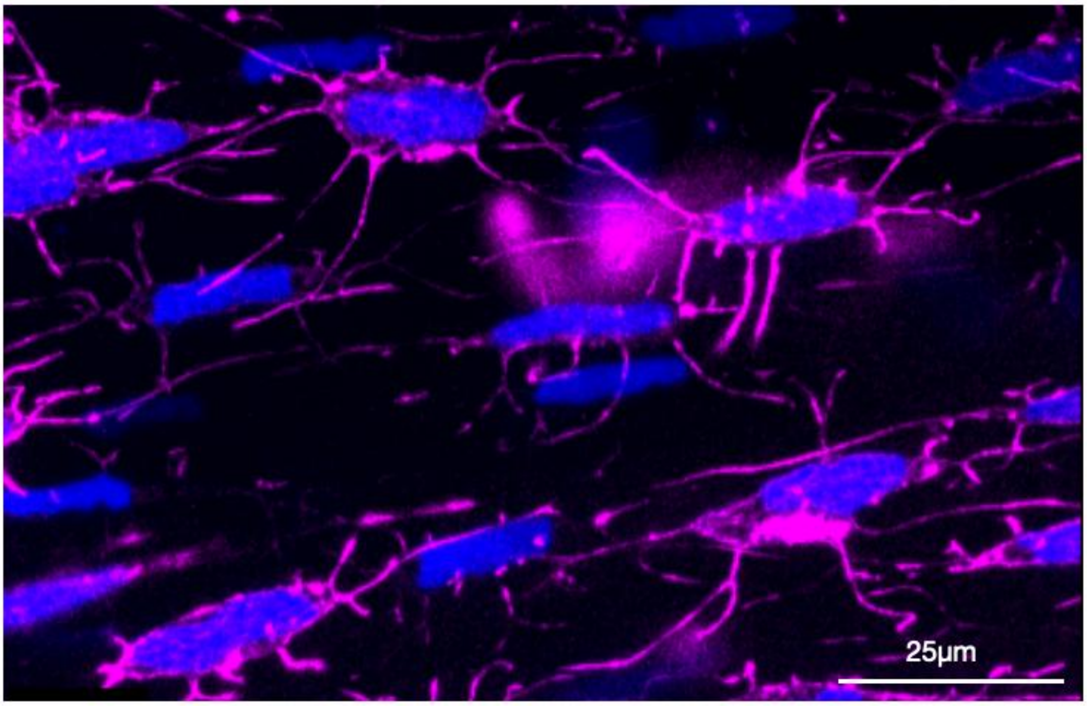

**Figure 1. Zebrafish osteocytes form a complex 3D network of dendritic processes densely organised in regions of higher mechanical stress.** Zebrafish dendritic processes (magenta) were stained with phalloidin and nuclei (blue) with DAPI. Image was acquired using a Stellaris confocal microscope.

### Printed Poster Abstracts

### PO1

Abstract Withdrawn

### P02

### Developing an Animal Model to Study the Role of Osteocytes in Hypogonadal Osteoporosis

Rachel L Wade[1], Donald R Dunbar[1], Anna Raper[1], Joanna Simpson[2], Natalie Z M Homer[2,3], Gurå T Bergkvist[1], Colin Farquharson[1], Jennifer A Fraser[1]

[1]Royal (Dick) School of Veterinary Sciences and Roslin Institute, Edinburgh, UK. [2]Mass Spectrometry Core, Edinburgh Clinical Research Facility, Institute for Neuroscience and Cardiovascular Research, Edinburgh, UK. [3]University/BHF Centre for Cardiovascular Science, Edinburgh, UK

### Abstract

During prostate cancer treatment (androgen deprivation therapy) and throughout the menopause, patients frequently present with osteoporosis and increased fracture risk, highlighting the impact of sex hormone deprivation on the skeleton. It is widely accepted this is caused by dysregulated osteoblast and osteoclast activity, which favours osteoclast-mediated bone resorption. In this study, the impact of androgen and oestrogen deprivation on osteocytes was explored, with the aim of ascertaining whether osteocytes may contribute to the skeletal frailty seen in hypogonadal osteoporosis.

To model sex hormone deprivation, 12-week-old male and female C57BL/6J mice were treated by subcutaneous depot injection with degarelix, a gonadotropin releasing hormone antagonist that blocks the HPG axis, or drug vehicle. Mice were sacrificed 28 days later and plasma and long bones were collected and processed for analysis by mass spectrometry, micro-CT, and RNA-Seq.

Liquid chromatography mass spectrometry analysis of plasma confirmed that degarelix treatment reduced circulating sex steroid levels, including testosterone levels in male mice (from 0.40 ± 0.06 ng/mL to 0.0102 ± 0.0005 ng/mL, P=0.03) and progesterone levels in female mice (from 4.7 ± 2.5 ng/mL to 0.36 ± 0.04 ng/mL, P=0.27). Degarelix treatment also caused significantly more weight gain in female mice (+8.3 ± 1.4%) compared to controls (+2.9 ± 1.3%, adjusted P=0.01), whilst degarelix treatment induced significant weight loss in male mice (-8.6 ± 0.8%) compared with weight gain in untreated controls (+6.6 ± 1.3%, adjusted P=0.01).

Micro-CT analysis confirmed degarelix treatment caused a significant (P<0.001) disruption of trabecular microarchitecture in male mice, with decreased trabecular thickness and number; increased trabecular separation and patterning factor; and reduced bone volume/tissue volume (BV/TV). In male mice, modest effects of degarelix were observed on cortical bone area, medullary area, and cortical thickness. RNA-Seq analysis of the cortical bone indicated some transcriptomic changes in male mice, however, large variability in expression of osteocyte genes within degarelix treated males (*Dkk1*, *Dmp1*, *Mepe*, *Sost*) made meaningful interpretation of the data challenging. In contrast, trabecular and cortical bone microarchitecture were unaffected by degarelix treatment in female mice (P>0.05) and minimal transcriptomic changes were detected between treated and control animals. Ongoing studies include quantification of circulating markers of bone turnover (P1NP, αCTx); osteocyte apoptosis and lacunar occupancy; and parameters of the lacuna-canalicular network.

Overall, this research highlights the sexually dimorphic response of the skeleton to degarelix and highlights further research is required to understand the role osteocytes play in hypogonadal osteoporosis.

### P03

### The Bone Phenotype of the *P2rx7*$^{KiKo}$ Mouse

Robin Rumney[1,2], Charles Wood[2], Dariusz Górecki[2]

[1]University of Southampton, Southampton, UK. [2]University of Portsmouth, Portsmouth, UK

### Abstract

The P2X7 receptor (P2X7R) has well-established roles in bone physiology, being responsible for maturation of bone forming osteoblasts and the fusion of bone resorbing osteoclasts. Here, we examine bones from our novel P2X7 knockin-knockout mouse (*P2rx7*$^{KiKo}$) and carry out functional analyses of isolated cells *in vitro*.

Tibiae from 8-week-old male C57BL/6, *P2rx7*$^{KiKo}$ and P2x7$^{-/-}$ mice were imaged with high resolution micro-CT (Zeiss Xradia); µCT data were analyzed with CTAN prior to histological processing for TRAP staining. Primary osteoblasts were characterized by Western Blot for P2X7 protein expression and alkaline phosphatase (ALP) activity and Alizarin Red (AR) staining for mineralization. Osteoclasts were derived from bone marrow precursors.

The morphometry of trabecular bone from both *P2rx7*$^{KiKo}$ and *P2rx7*$^{-/-}$ mice differed significantly from the wild-type C57BL/6 controls, but not from each other, despite a trend towards increased cortical bone volume in *P2rx7*$^{KiKo}$. TRAP staining revealed significantly decreased numbers of both osteoblasts and osteoclasts in *P2rx7*$^{-/-}$ mice, whereas in *P2rx7*$^{KiKo}$ mice osteoclast size was decreased. Osteoclast number *in vitro* was unchanged between C57BL/6, and *P2rx7*$^{KiKo}$. *In vitro* analyses of *P2rx7*$^{KiKo}$ primary osteoblasts revealed decreased P2X7 expression, higher ALP activity and increased AR mineralization staining compared to C57BL/6, controls.

In conclusion, partial knockdown of P2X7 in *P2rx7*$^{KiKo}$ mice results in a unique reduction of osteoclast size. The trend towards thicker cortical bone in *P2rx7*$^{KiKo}$ mice corresponds with an increase in mineralization activity in *P2rx7*$^{KiKo}$ osteoblasts *in vitro*. Therefore, the *P2rx7*$^{KiKo}$ mouse may be a useful model to enhance our understanding of the multiple roles for the P2X7R in bone.

### P04

### Restoration of Bisphosphonate-Inhibited Osteoblast Mineralisation with Ionic Medicine

Maria Florez-Martin, Kaveh Shakib, Gavin Jell

University College London, London, UK

### Abstract

Medication-related osteonecrosis of the jaw (MRONJ) is a serious complication of bisphosphonate therapy, but no treatment currently targets the impaired bone formation that may contribute to its development [1,2]. Recent systematic review evidence indicates that zoledronate (ZOL), the most potent bisphosphonate, directly affects osteoblastic cell behaviour *in vitro*, supporting osteoblast dysfunction as a relevant therapeutic target [3]. We therefore tested whether dissolution products from 45S5 Bioglass® (45 wt% $SiO_2$, 24.5 wt% $Na_2O$, 24.5 wt% CaO, and 6 wt% $P_2O_5$) could restore ZOL-inhibited osteoblast biomineralisation, and whether any benefit could be explained by Si alone.

Primary rat calvarial osteoblasts were cultured under continuous ZOL exposure and treated with 45S5-conditioned medium or sodium metasilicate matched for Si concentration. Outcomes included proliferation, metabolic activity, alkaline phosphatase (ALP), vascular endothelial growth factor (VEGF), reactive oxygen species (ROS) production, Alizarin Red staining (ARS), Raman spectroscopy, and interferometry. The latter was implemented as a non-contact 3-D surface-mapping approach to quantify bone nodule height and volume alongside conventional 2-D mineralisation assays.

ZOL impaired osteoblast function in a dose- and time-dependent manner, and all concentrations inhibited biomineralisation. Treatment with 45S5 (0.25 mM [Si]) significantly increased mineralised area relative to ZOL alone in cultures exposed to low-dose ZOL (0.067 µM; $p<0.0001$). In addition to 2-D staining, interferometry showed partial recovery of bone nodule volume relative to ZOL alone ($p<0.001$), while Raman suggested preservation of several mineral-associated spectral features in 45S5-treated ZOL cultures. In contrast, Si alone increased mineralised area in ZOL-free cultures but did not restore biomineralisation or nodule volume under ZOL exposure. Neither 45S5 nor Si restored ZOL-associated reductions in proliferation, metabolic activity, or ALP, and 45S5 did not reverse the stress-associated changes seen in VEGF or ROS.

These findings suggest that 45S5-derived ionic dissolution products can partially rescue ZOL-impaired osteoblast biomineralisation, and can be a promising biomaterial strategy for MRONJ management. As this effect was not reproduced by Si alone, the restorative response is more likely to depend on the combined ionic effect of 45S5 than on a single-ion mechanism. The study also highlights the value of combining conventional 2-D mineralisation assays with 3-D interferometric and compositional analyses, as apparent recovery in stained area did not equate to full restoration of bone nodule volume or broader osteoblast function.

[1] A. Alsalih, 2021, doi:10.3390/dj90800

[2] A. Abdolrahmani, 2024, doi:10.1007/s00520-024-08388-4.

[3] M. Florez-Martin, 2026, doi:10.1016/j.bone.2026.117796

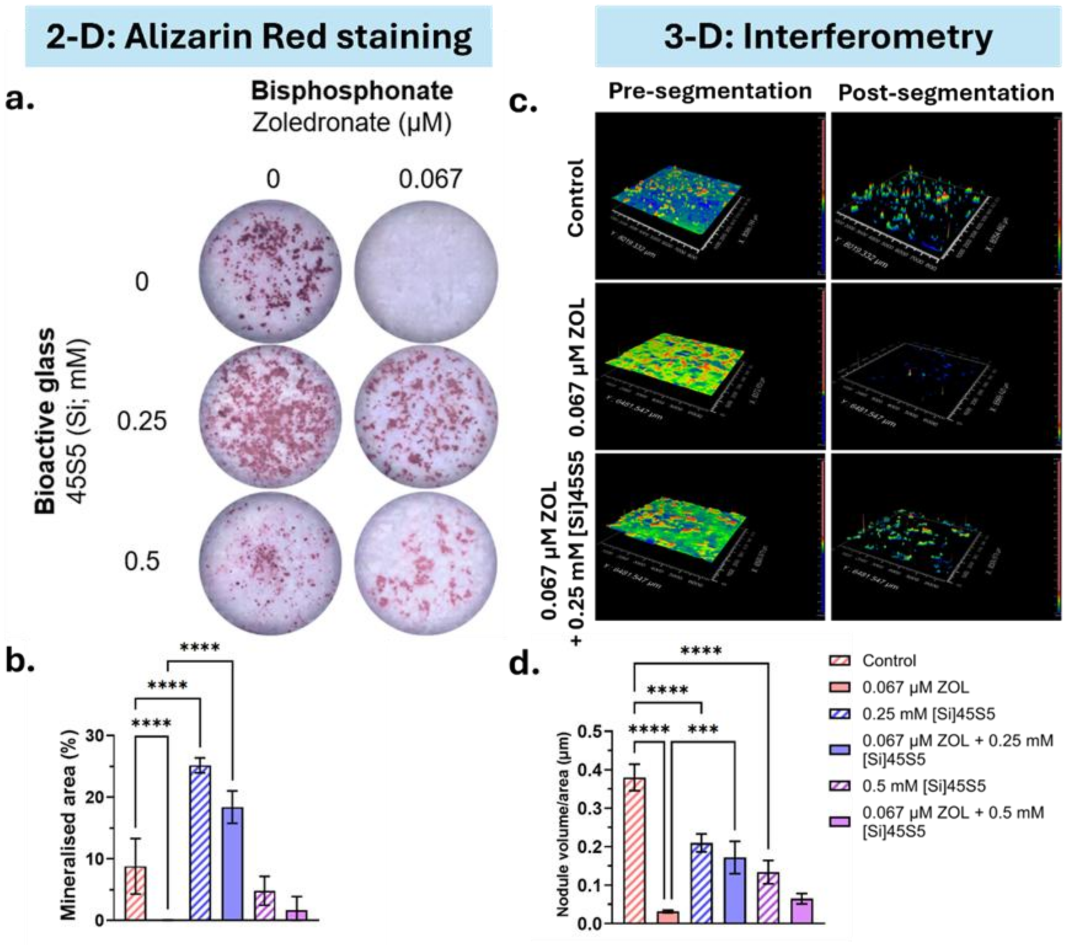

*Figure 1.* ***Restoration of ZOL-induced inhibition of mineralisation and bone volume with 45S5.*** *(**a**)ARS. (**b**)Quantification of mineralised area (**c**)Nodule visualisation through height segmentation. (**d**)Quantification of nodule volume.*

### P05

Abstract Withdrawn

### P06

### Effect of 3D-printed PLA-TCP-$SiO_2$ Scaffolds Associated with Photobiomodulation Therapy on Bone Tissue Repair

Gileade Pereira Freitas[1], Laiz Moreira de Paula[1], Higor Venancio de Melo[1], Natalia Queiroz Melo[1], Ana Vitoria Lobo de Souza[1], Eduardo Henrique Backes[2], Virgilio Moreira Roriz[1], Helena Bacha Lopes[1]

[1]School of Dentistry, Federal University of Goias, Goiania, Brazil. [2]Departament of Materials Engineering, Federal University of São Carlos, São Carlos, Brazil

### Abstract

Autogenous grafts remain the gold standard for treating bone defects, as they provide osteoinduction, osteoconduction, and osteogenesis. However, this therapy has limited availability, variable resorption rates, and may cause donor-site morbidity, infections, and other postoperative complications. Several composite materials have been investigated for scaffold fabrication to promote complete bone regeneration in critical-sized defects, potentially replacing autogenous grafts. Among these, the combination of polylactic acid (PLA), β-tricalcium phosphate (TCP), and silicon dioxide ($SiO_2$) has been shown, in vitro, to enhance cell proliferation, alkaline phosphatase activity, calcium deposition, and the overexpression of osteoblastic marker genes. However, it is necessary to demonstrate its effectiveness and evaluate its behavior in vivo to ensure optimal performance and enable future clinical applications. In addition, photobiomodulation therapy (PBMT) may further enhance bone regeneration by increasing osteocyte numbers, stimulating bone matrix deposition, and accelerating bone repair. Therefore, this study aimed to evaluate, in vivo, the tissue response to PLA-TCP-$SiO_2$ scaffolds associated or not with PBMT in bone tissue formation. The Ethics Committee approved this study on the Use of Animals of the Federal University of Goiás, Brazil. Critical-sized bone defects (5 mm in diameter) were created in the calvaria of male rats weighing approximately 250 g and treated with 3D-printed PLA-TCP-$SiO_2$ scaffolds, with or without PBMT. After 28 days, the newly formed bone was assessed by micro-CT analysis. Data were analyzed using Student's *t*-test ($n = 8$, $p < 0.05$). The morphometric parameters obtained from micro-CT analysis showed that bone volume, bone volume fraction, bone surface, trabecular number, trabecular thickness, and trabecular separation did not differ significantly between defects treated with 3D-printed PLA-TCP-$SiO_2$ scaffolds alone and those treated with scaffolds associated with PBMT ($p$ = 0.486, 0.486, 0.372, 0.377, 0.776, and 0.152, respectively). In conclusion, 3D-printed PLA-TCP-$SiO_2$ scaffolds induced bone formation; however, no additional benefit was observed when combined with PBMT for the treatment of bone defects.

### P07

### Guided Bone Regeneration Using a Hydroxyethyl Cellulose Adhesive Strip: A Study in a Critical Bone Defect

Helena Bacha Lopes, Higor Venancio de Melo, Laiz Moreira de Paula, Natalia Queiroz Melo, Ana Vitoria Lobo de Souza, Virgilio Moreira Roriz, Gileade Pereira Freitas

School of Dentistry, Federal University of Goias, Goiânia, Brazil

**Abstract**

Maxillofacial bone defects remain a clinical challenge, compromising function, aesthetics, and quality of life. Although autogenous, allogeneic, and xenogeneic grafts are commonly used, each presents important limitations. In this sense, tissue engineering strategies have emerged as promising alternatives for bone regeneration, combining principles of biology, chemistry, engineering, and materials science to develop biomaterials that act as biological substitutes, restoring, maintaining, or improving the function of tissues and organs. The hydroxyethyl cellulose adhesive strip is widely used in dentistry to promote oral mucosal wound healing; however, its potential role in bone defect regeneration has not yet been investigated. This study aimed to evaluate bone formation induced by a hydroxyethyl cellulose adhesive strip using the guided bone regeneration technique in a critical-sized calvarial defect model. The Ethics Committee approved the study on the Use of Animals of the Federal University of Goiás, Brazil. Critical-sized defects (5 mm in diameter) were surgically created in the calvaria of male Wistar rats (150–250 g) and were either left to heal with a blood clot (control group) or treated with the hydroxyethyl cellulose adhesive strip. After 28 days, bone formation was assessed by micro-computed tomography (micro-CT). Morphometric parameters were analyzed using Student's *t*-test ($n = 8$, $p < 0.05$). Micro-CT analysis demonstrated that bone volume, bone volume fraction, bone surface, trabecular number, trabecular thickness, and trabecular separation were significantly higher in the control group compared to defects treated with the hydroxyethyl cellulose adhesive strip ($p = 0.006$, 0.017, 0.006, 0.045, 0.013, and 0.012, respectively). In conclusion, the hydroxyethyl cellulose adhesive strip did not enhance bone formation compared to spontaneous healing with a blood clot in critical-sized bone defects.

### P08

### Ultrasound Responsive Agent for Delayed/ Non-Union Fracture Healing

Meng Chen Michelle Li[1], Dariusz Kosk[1], Janos Kanczler[1], Dario Carugo[2], Eleanor Stride[2], Nick Evans[1]

[1]University of Southampton, Southampton, UK. [2]University of Oxford, Oxford, UK

### Abstract

### Introduction

Non-union fractures occur in up to 10% of cases and frequently require invasive and costly secondary surgery. There is a need for non-invasive, targeted drug-delivery systems that can enhance bone repair. Phospholipid-coated gas microbubbles are highly responsive to ultrasound and can carry or be co-administered with therapeutic agents. Their rapid oscillation under ultrasound exposure enables spatially targeted drug release. This study investigated whether ultrasound can effectively stimulate microbubbles at bone-defect sites and identified optimal timing for local drug delivery.

### Methods

4mm calvarial defects were created in 3-month-old MF1 mice and placed on an acoustically transparent platform above a water bath containing ultrasound transducer. Saline or SonoVue® (clinical contrast agent) were injected intravenously. Focused ultrasound was applied to calvaria for 10 minutes and passive cavitation detection (PCD) was used to record acoustic emissions. Assessments of PCD and H&E staining of bone samples were made on D3, 7, 14 and 28.

### Results and Discussion

PCD measurements showed higher acoustic-emission amplitudes after SonoVue® injection compared with saline, with signal decay consistent with microbubble destruction. Emission power from SonoVue® was greatest on D3, decreased on D7, increased again on D14, and declined by D28. SonoVue® group exhibited a significantly greater increase in total signal on D3 (8.06±2.6%) compared with the saline group (1.12±1.9%; p=0.005). Changes in PCD signals likely correspond to changes in tissue perfusion at different healing stages. H&E images show bridging and tissue growth between the defects.

### Conclusions

These results confirm that microbubbles were able to perfuse the vasculature at defect site and there was sufficient ultrasound transmission to stimulate the microbubbles. Temporal changes in perfusion suggest that microbubble-mediated drug delivery could be timed to specific healing stages, with D14 emerging as an optimal window due to enhanced microbubble activity without interference with early inflammation.

**Acknowledgement:** Medical Research Council (Ref: MR/X009793/1)

### P09

Abstract Withdrawn

### P10

### Investigation of Novel Chitin Derived Scaffolds for Bone Regeneration

Suheyla Kose, Robin Rumney

University of Southampton, Southampton, UK

### Abstract

**Background:** Impaired healing of bone defects may cause bone non-union which is associated with long recovery times. Current treatment options such as autologous bone grafts require additional invasive surgery so alternative therapeutic options are needed, which could include biocompatible scaffolds inspired by nature. The strongest known biogenic material has been found from teeth of the Common Limpet (*Patella vulgata*). Recent studies have successfully generated a biomimetic material based on limpet tooth using a chitin-based scaffold. We hypothesised that chitin might be a compatible scaffold for osteogenesis.

**Aims:** The aim of this project is to assess whether chitin scaffolds derived from shrimp shells are compatible with osteoblasts for osteogenesis.

**Methods:** Chitin derived from shrimp shells were sonicated, freeze dried and solubilised in HFIP for electrospinning into scaffolds. Biocompatibility and osteogenesis were assessed using MG-63 cells seeded into 5mm chitin discs. Crystal violet staining was used to assess cell adhesion and viability. MG-63 cells were cultured on chitin scaffolds in osteogenic media; chitin scaffolds were also incubated with extracellular vesicles (EV) isolated from MG-63 cells. Alkaline phosphatase and mineralisation were quantified with NBT-BCIP staining and Alizarin red staining respectively. RNA was extracted from cells grown on chitin scaffolds and control non-chitin substrates for cDNA synthesis, PCR and qPCR on chitin specific and osteogenic transcripts.

**Results:** Crystal violet staining confirmed cell adherence to the chitin scaffolds. FBS treatment of chitin increased both cell adherence and mineral deposition on chitin ($p < 0.05$). NBT-BCIP staining demonstrated alkaline phosphatase activity on cell seeded scaffolds but not those treated with EVs. Alizarin red staining confirmed calcium deposition on both cell-seeded scaffolds and EV treated scaffolds. PCR confirmed the expression of Chitinase-3-like protein 1 (CHI3L1) and Collagen Type I Alpha 1 Chain (COL1A1) but qPCR found no significant effects of chitin substrates upon expression of these transcripts.

**Conclusions:** Chitin scaffolds can support MG-63 cell growth and mineralization, the latter of which can be mediated by EVs alone. FBS treatment of chitin enhances mineralization by MG-63 cells suggesting that in clinical applications, autologous serum could be used to enhance biocompatibility of chitin.

### P11

Abstract Withdrawn

### P12

Abstract Withdrawn

### P13

### Osteochondral Cement Line Mineralisation and Nanoindentation Modulus are Similar to Well-Mineralised Bone and Articular Calcified Cartilage

Michael Doube, Andy Bushby, Alan Boyde

Queen Mary, University of London, London, UK

### Abstract

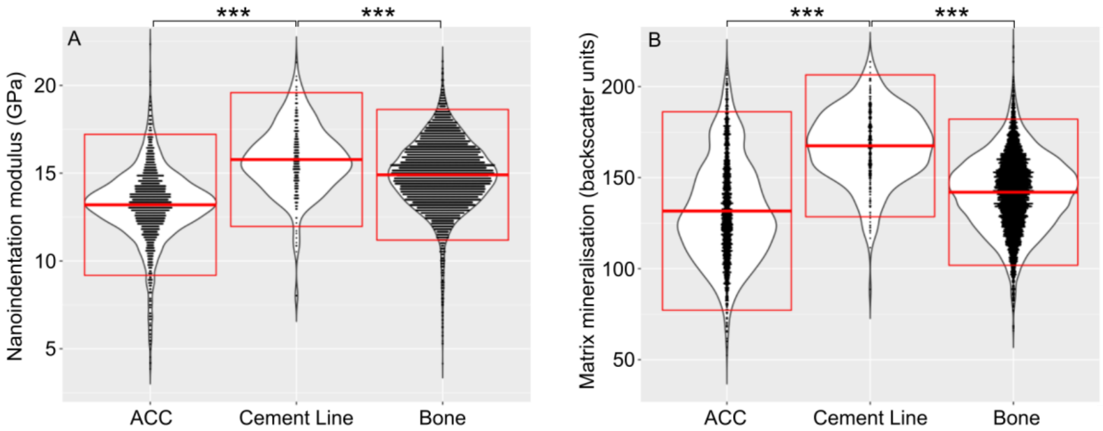

The osteochondral cement line is the boundary between bone and cartilage, where adhesion between the two tissues occurs. Remodelling by osteoclasts and osteoblasts, as in secondary osteonal remodelling of bone, integrates articular calcified cartilage (ACC) with subchondral bone (SCB), leaving a cement line, visible as a mineralised discontinuity between ACC and SCB. The degree of cement line mineralisation relative to ACC and SCB is unknown, but is sometimes described as 'hypermineralised'. Collagen is an important component of cement line, so it is not solely mineral [1]. Demineralised thin sections do not normally separate along the cement line during histological processing, suggesting that mineral has a partial at most contribution to cement line integrity.

Osteochondral blocks were cut from twelve equine distal third metacarpal bones, embedded in polymethylmethacrylate and diamond ultramilled flat. Twenty-four arrays of 708-1492 sites (two arrays per specimen, ~20 × 50 sites per array, 20 µm spacing) extending from hyaline cartilage to SCB were nanoindentation tested. Fields were imaged in quantitative backscattered scanning electron microscopy, the nanoindentation sites classified as ACC, SCB or cement line and mineralisation density recorded per site. Cement line micromorphology was catalogued in qBSE across the joint width.

Mean (±sd) nanoindentation modulus ($E$) and mineralisation density ($d_m$) were higher ($p < 0.001$) in cement line ($E$ 15.8 ± 1.9 GPa; $d_m$ 167.4 ± 19.8) than ACC ($E$ 13.2 ± 2.0 GPa; $d_m$ 131.4 ± 27.5) and SCB ($E$ 14.9 ± 1.9 GPa; $d_m$ 141.9 ± 14.9), however, cement line mineralisation and nanoindentation modulus ($E_{max}$ 21.3 GPa; $d_{mmax}$ 218.5) were within the same ranges as ACC ($E_{max}$ 22.3 GPa; $d_{mmax}$ 210.1) and bone ($E_{max}$ 21.4 GPa; $d_{mmax}$ 222.5). Distinctive mineralisation patterns appeared, such as a band of high mineralisation in SCB a few µm from the interface, another band in SCB at the interface and another band a few µm from the interface in ACC, or any combination or absence of these bands.

Osteochondral cement line has mineral and material properties that are not unusual to find in bone and ACC, reflecting the tissues' common origin and composition. Mineralisation patterns may offer clues to the formation mechanisms of osteochondral cement line.

1.Hayat K, Marr N, Mak KKL, Doube M. 2025 Type-I and -II collagens from bone and cartilage colocalize at the osteochondral cement line. *Bone & Joint Research* **14**, 735–744. (doi:10.1302/2046-3758.148.BJR-2024-0396.R1

### P14

Abstract Withdrawn

### P15

### Transgenerational Effects of Maternal Resistance Exercise on Musculoskeletal in Mice Offspring

Anna Julia Paes de Barros, Vithor Hugo Carvalho Peixoto, Edgar Willibaldo Allebrandt Neto, Alison Henrique Ferreira, Maria Gabriela de Mendonça Mazetti, Marcos Almeida de Souza, Suelem Franca Lemes, Roberto Lopes de Souza

UFMT, Cuiabá, Brazil

### Abstract

**Background:** The intrauterine environment exerts profound and lasting epigenetic effects on offspring phenotype. Physical exercise during pregnancy programmes offspring physiology beyond the postnatal phase. Whilst maternal aerobic exercise benefits are increasingly documented, combined transgenerational effects on skeletal muscle and bone remain inadequately explored. This investigation examines how maternal resistance exercise during gestation programmes musculoskeletal development in offspring (F1-F2), emphasizing epigenetic inheritance and phenotypic expression across successive generations.

**Methods:** Pregnant C57BL/6 mice were assigned to sedentary (SED) or exercise (EXER) groups. The EXER group performed structured resistance training (vertical ladder climbing, 50-75% body weight) three times weekly throughout gestation. Female offspring from F1 and F2 generations were analysed at 12 weeks. Skeletal muscle morphometry (Biceps brachii, Gastrocnemius, Soleus) was assessed in F1 via histomorphometric analysis, quantifying muscle fibre perimeter and length. Tibial trabecular bone microarchitecture was analysed in both generations using micro-computed tomography (micro-CT), evaluating bone volume fraction (BV/TV) and structural model index (SMI), trabecular thickness and number.

**Results:** In the F1 generation, offspring from exercised dams (EXER-F1) exhibited significant skeletal muscle fibre hypertrophy, with increased fibre perimeter and length across all analysed muscles compared to the SED-F1 group ($p < 0.05$). No significant differences were observed in muscle fibre number, suggesting maternal exercise exerts hypertrophic rather than hyperplastic effects. In bone, whilst the F1 generation demonstrated early signs of trabecular reorganisation, the most profound and mechanically significant effects were observed in the F2 generation. EXER-F2 offspring displayed a markedly superior bone phenotype, characterised by a significant increase in bone volume (BV/TV: 20.36% vs. 18.42% in SED-F2) and a substantially more robust, plate-like trabecular architecture, evidenced by a dramatically lower structural model index (-0.84 vs. 1.95 in SED-F2, $p < 0.05$). These findings indicate progressive amplification of skeletal benefits across generations.

**Conclusion:** Maternal resistance exercise induces a positive and heritable programming effect on the musculoskeletal system that manifests progressively across generations. The benefits are evident in the first generation through significant muscle fibre hypertrophy and are markedly amplified in the second generation, which exhibits substantially more robust bone microarchitecture and enhanced mechanical competence. These findings suggest that maternal exercise can promote long-term, transgenerational benefits for skeletal and muscle health, likely mediated by epigenetic inheritance , offering a compelling preventive strategy against age-related musculoskeletal disorders. This work extends the paradigm of developmental programming to encompass the musculoskeletal system and underscores the profound impact of maternal lifestyle on offspring health trajectories.

### P16

Abstract Withdrawn

### P17

### Associations Between Biomechanical Markers of Walking Onset and Bone Strength Across Later Childhood

Alex Ireland[1], Christopher Ruff[2]

[1]Manchester Metropolitan University, Manchester, UK. [2]Johns Hopkins University, Baltimore, USA

### Abstract

Previous research has shown that earlier walking onset is associated with greater bone strength in infancy (Ireland *et al* 2014, Bone), but it is unknown whether these associations are consistent across childhood. The ratio of femur to humerus polar section modulus (PSM), a measure of bone torsional and average bending strength, increases rapidly around walking onset age. It has therefore been proposed as a marker of the onset of independent walking.

To further investigate this issue, we employed data from the Denver Longitudinal Growth Study, in which limb radiographs were taken in 20 individuals (10 female) from near birth through late adolescence. PSM was determined from cortical and periosteal breadth measurements taken at 50% and 40% of distal-proximal bone length in the femur and humerus, respectively. Both values were adjusted for body size as the product of bone maximal length and body mass (Ruff 2000, Journal of Human Evolution). From plots of femur/humerus PSM against age, individuals with a rapid increase prior to 18 months were grouped as early walkers ($n$=11) and those after 18 months as late walkers ($n$=9). Associations between walking age group and size-adjusted femur and humerus PSM were assessed using linear mixed effects models with adjustment for sex; sex and age interactions with walking age group were also examined.

Complete data were available in almost all participants at six-monthly intervals from six to fourteen years of age, and then at yearly intervals until seventeen years. In total, data obtained from 377 femoral and 373 humeral radiographs were assessed. Early walking age was associated with higher femoral PSM ($P$=0.049), and there was no evidence of sex or age interactions (both $P$>0.2). In contrast, there was no main association or sex interaction between walking age group and humerus PSM ($P$>0.3). However, an age interaction ($P$ =0.001) showed a smaller increase in humerus PSM with age in early walkers.

This study assessed, for the first time, associations between biomechanical markers of walking onset and bone strength characteristics across childhood. We found that later inferred walking age was associated with lower femoral but not humeral bone strength, and that these associations were consistent across mid- and late childhood. Conversely, humerus strength increased less rapidly across childhood in early walkers. This may reflect preferential skeletal adaptation of the lower limbs following early loading during locomotion, with incomplete catch-up growth among late walkers.

### P18

### Impact of the Bone Microenvironment on Multiple Myeloma Dormancy

Hannah Smith, Alexandria Sprules, Haider Al-Janabi, Chloe Harris, Abigail Foston, Alanna Green

University of Sheffield, Sheffield, UK

### Abstract

Multiple myeloma is the second most common form of blood cancer, but it is currently incurable due to a subpopulation of quiescent myeloma cells. These ‘dormant’ cells are a type of non-cycling cancer cell which are resistant to chemotherapy. They remain within the bone marrow niche until they activate and cause relapse. Interactions between these ‘dormant’ myeloma cells and the surrounding microenvironment are currently unknown, with studies suggesting osteoblast-like bone cells may be involved in promoting dormancy, while bone forming osteoclasts could be involved with reactivating the dormant cells.

To investigate regulatory pathways and interactions for dormant myeloma cells, single cell RNA sequencing was performed on a previously developed U266 mouse model. NSG mice were inoculated with and without U266 myeloma cells. When the tumours reached high abundance, a subset of the mice were treated with a combination of lenalidomide and bortezomib for two weeks. After treatment, bone marrow was extracted from the femurs and tibias and bone fragments were collagenase-digested to obtain bone lining cells. To ensure rare dormancy niche cells were captured, these cells were enriched prior to barcoding (including dormant and proliferative myeloma cells, CD31-Sca-1+ stem cells, and CD45-CD51+ osteoblast-lineage cells) using fluorescence activated or magnetic cell sorting. The cells were then processed using both the 10X Genomics Chromium GEM-X system, and the Parse Bioscience Evercode WT system before sequencing.

Using the Parse Bioscience Trailmaker software, distinct bone clusters have been generated, showing separation of bone matrix related and cartilage related cells. This is evidenced by the upregulated relative gene expression of *Col1a1* and *Bglap* (logFC 2.42 and 1.69 respectively) in the bone-matrix related clusters, and *Acan* and *Col2a1* (logFC 2.187 and 1.875 respectively) in the cartilage related clusters. Ongoing analysis of the single cell RNA sequencing data from these two systems will generate insight into not just the innate transcriptome of the dormant and proliferative myeloma cells, but also the cells of the surrounding microenvironment from mice with myeloma and healthy mice. Understanding the unique transcriptome of the dormant microenvironment will generate targets for the development of ‘dormalytics’; specialised treatments that directly target dormant cells, and/or the dormancy niche. Targeting dormant cells will result in reduced relapse rates and improved outcomes for myeloma patients.

### P19

### Phosphate-Specific Regulation of Osteoblast Mineralisation Informs Design of Regenerative Bone Scaffolds

Joseph Weightman[1,2], Karthik Vaidya[2,1], Mohamed Patel[3], Ellie Northall[2], Pola Goldberg-Oppenheimer[3], Amy Naylor[2], Liam Grover[1,3]

[1]Healthcare Technologies Institute, University of Birmingham, Birmingham, UK. [2]Department of Inflammation and Aging, University of Birmingham, Birmingham, UK. [3]School of Chemical Engineering, University of Birmingham, Birmingham, UK

### Abstract

Phosphate is essential for bone formation and mineralisation; however, the influence of its chemical form on osteoblast function and mineral development has not been extensively investigated. We hypothesise that phosphate chemistry plays a key role in regulating these processes. Understanding how cells process different phosphate sources is essential for replicating physiological bone formation and for designing biomaterials that emulate native mineralisation pathways and support repair. This study examined how distinct phosphate donor sources influence osteoblast differentiation, mineral formation, and mineral composition.

Murine pre-osteoblastic MC3T3-E1 cells, human adipose-derived stem cells (ADSCs), osteosarcoma SAOS-2 cells, and human fetal osteoblast hFOB cells were cultured under osteogenic conditions supplemented with defined phosphate sources, including orthophosphate, β-glycerophosphate, ATP, and phosphocholine. To assess translational relevance, 3D self-structuring bone models were also used to evaluate whether phosphate-dependent mineralisation responses observed in 2D cultures are maintained in three-dimensional environments. Mineral deposition was evaluated using alizarin red staining and phase-contrast imaging to visualise matrix formation. Raman spectroscopy characterised mineral composition through phosphate ($\nu_1$ and $\nu_3$) and carbonate vibrational band analysis, providing information on mineral maturity and crystallinity. Expression of osteogenic markers, including *ALPL*, *RUNX2*, *COL1A1*, *BGLAP*, was quantified by qPCR to determine transcriptional responses to distinct phosphate environments.

Phosphate source strongly influenced the extent and organisation of mineral deposition. Phosphocholine supplementation generated uniform, highly organised mineralised matrices, whereas orthophosphate produced more diffuse mineral clusters, indicating divergent mineral growth kinetics. Gene expression analysis revealed phosphate-specific regulatory effects, with lower *ALPL* and *BGLAP* expression in orthophosphate cultures, while phosphocholine significantly enhanced *BGLAP* expression, consistent with greater osteoblast maturation and upregulated production of bone-matrix protein. In 3D models, Raman spectral analysis confirmed compositional differences, showing shifts in phosphate and carbonate peaks reflective of altered mineral phase development and substitution chemistry.

Overall, these findings identify phosphate chemistry as a central regulator of osteoblast-driven mineralisation and mineral phase evolution. The study provides insight into phosphate utilisation during bone formation and informs the rational design of regenerative biomaterials that more accurately reproduce physiological mineralisation pathways.

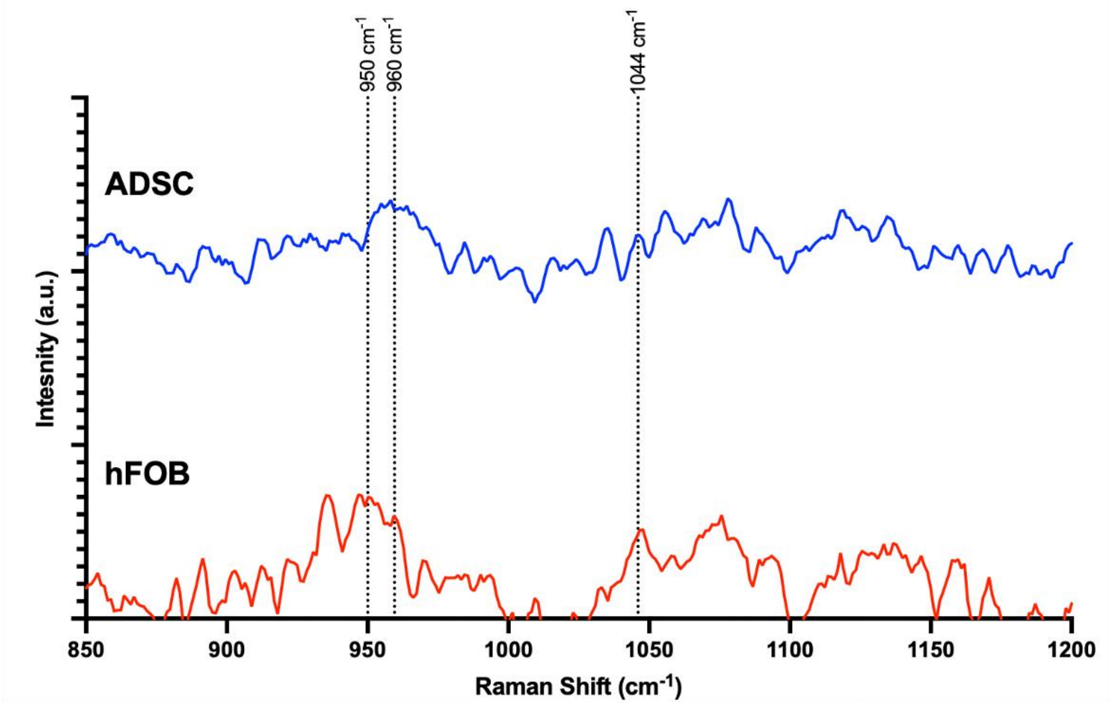

**Figure 1. Raman spectral analysis analysis of self-destructing bone model derived from hFOB and ADSC cultures at 12 weeks.** hFOB constructs show peaks at 950 $cm^{-1}$ and 960 $cm^{-1}$, corresponding to amorphous calcium phosphate and stoichiometric hydroxyapatite. ADSC models shift toward 1044 $cm^{-1}$, indicating increased carbonate substitution and formation of a more biological substituted apatite phase.

### P20

### DNA Damage in Dormant Myeloma Cells in the Bone Microenvironment - Chemotherapy Evasion Mechanism or Potential New Target?

Abigail Foston, Haider Al-Janabi, Alexandria Sprules, Victor Canuas-Landero, Helen Bryant, Alanna Green

The University of Sheffield, Sheffield, UK

### Abstract

### Introduction

Multiple myeloma is a prevalent haematological cancer but remains incurable despite advances in research and treatments. Currently approved therapeutics often lead to a state of 'minimal residual disease' (MRD), but patients inevitably relapse, initiated by reactivation of dormant, chemo-resistant myeloma cells. Dormancy is controlled by bone microenvironment cells, such as osteoblasts and osteoclasts, however the possible intrinsic mechanisms of dormancy are unknown. Entry into dormancy may be a protective mechanism to prolong cellular survival, possibly triggered by DNA damage. This work aims to compare DNA damage levels between proliferating and dormant myeloma cells and assess the potential for targeting the DNA damage response (DDR) in dormant cells.

### Methods

To assess DNA damage levels *in vivo*, an immunocompetent model (5TGM1-GFP-Luc cells and C57BL/KaLwRij mice) and an immunocompromised model (U266-GFP-Luc cells and NSG mice) were studied. C57BL/KaLwRij mice did not receive treatment, and NSG mice received standard of care (SOC) chemotherapy 50mg/kg lenalidomide and 0.75mg/kg bortezomib. Tumour was monitored by bioluminescent imaging. Flow cytometry was used to identify dormant cell populations using DiD-retention, AXL and p21 expression. DNA damage levels were assessed by γH2AX staining of bone marrow samples.

*In vitro* comparisons were made between dormant and proliferating cells after 7 days culture, using flow cytometry and immunofluorescence staining for γH2AX. $DiD^{Hi}$ (dormant) and $DiD^{Low}$ (proliferating) cell populations were isolated by fluorescence activated cell sorting (FACS). DNA damage foci were quantified using ImageJ. Activity of DNA damage response inhibitor drugs (olaparib, abemaciclib, berzosertib and Ku55933) on proliferating myeloma cells was investigated using resazurin viability assay 1 week after dosing. Dormant cell vulnerability was assessed by flow cytometry using annexin V staining.

### Results and Discussion

NSG and C57BL/KaLwRij models exhibited significantly increased levels of DNA damage (γH2AX+) in dormant cell populations versus proliferative. *In vitro* experiments supported this, where γH2AX foci were significantly higher in the $DiD^{Hi}$ groups compared to $DiD^{Low}$ in both U266 and 5TGM1, indicating dormant myeloma cells have increased levels of DNA damage.

Abemaciclib (CDK4/6 inhibitor), berzosertib (ATR inhibitor) and Ku55933 (ATM inhibitor) dose-dependently inhibited viability in proliferating cells. PARP inhibitor olaparib reduced viability in BRCA-deficient 5TGM1 cells, but not U266 cells.

### Conclusion

Dormant myeloma cells exhibit higher levels of DNA damage across modelling approaches. Thus, targeting the DDR could be a promising approach to target chemo-resistant dormant myeloma cells.

### P21

### Agent-Based Numerical Model of Metastatic Bone Remodeling

Claire Villette[1,2], Liesbet Geris[2,3]

[1]Queen Mary University of London, London, UK. [2]KU Leuven, Leuven, Belgium. [3]University of Liege, Liege, Belgium

### Abstract

### Introduction

Bone metastases (BMs) severely disrupt bone remodeling by shifting the balance between osteoclast mediated resorption and osteogenic bone formation toward excessive osteolysis. Mechanical loading induces opposing, anabolic effects [1]. To support the investigation of loading regimens with therapeutic potential, this study developed a computational model of load induced bone remodeling in the presence of metastatic breast cancer cells.

### Methods

A hybrid cellular automaton (HCA) model was implemented in Python, integrating three interconnected modules:

1- The cell module used a cellular automaton [2] to describe migration, proliferation, death, and functional activity of osteogenic cells, osteoclasts, and cancer cells. Logistic functions defined the influence of mechanical stimulation and receptor occupancy on ECM-modifying behavior and biochemical signaling.

2- The biochemical module used partial differential equations (PDE) describing diffusion and binding kinetics of RANKL, OPG and PTHrP (key drivers of bone remodeling and cancer modulation).

3- The mechanics module solved a quasi static equilibrium problem using a hyperelastic neohookean constitutive law. Tetrahedral meshes with ~4.5 µm resolution were used, and von Mises stress served as the mechanical stimulus regulating cellular responses.

All PDE problems were solved alongside the cellular automaton using the FEniCSx solver.

### Results

As proof of concept, predicted RANKL and OPG concentrations, integrated across the model volume, were compared with gene-expression data from in-vitro layered hydrogel cultures of osteogenic cells, osteoclasts, and breast cancer cells [3]. Both static and dynamic loading (0.5% uniaxial compression) were evaluated. The model captured the experimentally observed order of magnitude increase in RANKL/OPG ratio in the presence of cancer cells, and its strong reduction under dynamic loading (Figure 1).

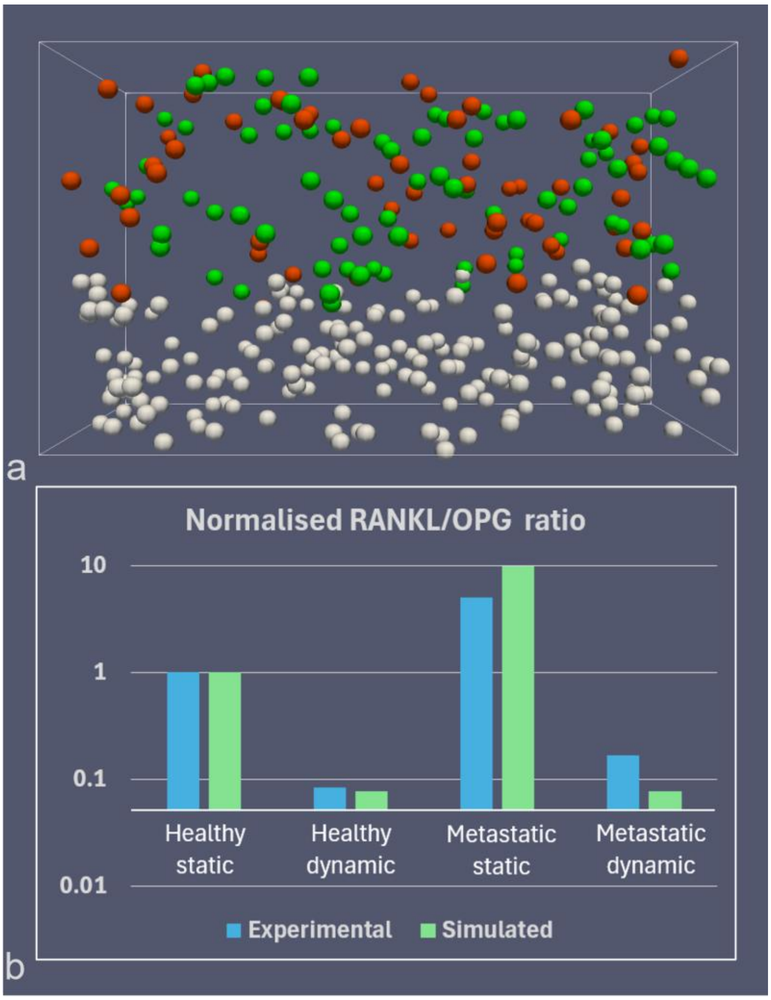

**Figure 1**: (a) Model slice showing layered osteogenic cells (white), osteoclasts (red), and cancer (green) (b) Simulated RANKL/OPG concentration ratio compared to experiments.

**Discussion**

An HCA framework was implemented which reproduces key cancer-induced perturbations of bone remodeling signals and their modulation by mechanical stimulation. Further work involves investigation of loading regimens with therapeutic potential.

**References**

1. Lynch et al., 2013. Journal of Bone and Mineral Research, 28(11), pp.2357-2367
2. Lai et al., 2022. International Journal for Numerical Methods in Biomedical Engineering 38.1, e3542
3. Kumar et al., 2024. Cell Reports 43.5

**Acknowledgements:**

The authors would like to thank **FWO** for providing financial support to this project.

### P22

### Dormant Myeloma Cells can be Killed Through Oxidative Stress in the Bone Niche

Chloe Harris[1], Haider Al-Janabi[1], Sherif El-Khamisy[1,2], Alanna Green[1]

[1]University of Sheffield, Sheffield, UK. [2]University of Bradford, Bradford, UK

### Abstract

Multiple myeloma is an incurable cancer of plasma cells in the bone marrow. Myeloma treatments can induce stable disease, but do not fully eradicate disease. Cancer cell dormancy presents one of the major challenges in overcoming inevitable therapy resistance and relapse. The bone microenvironment directly influences dormancy induction, maintenance, therapy resistance and regrowth. Yet, there are no 'dormalytic' therapeutics clinically approved for myeloma. Therefore, identification of both intrinsic and microenvironmental targetable weaknesses remains paramount to eliminate dormant cells and improve patient outcomes. We found elevated reactive oxygen species (ROS) in dormant myeloma cells compared to proliferating myeloma, contrasting known low ROS levels in healthy quiescent bone marrow haematopoietic stem cells. Therefore, we aimed to determine whether oxidative stress presents a targetable weakness of dormant myeloma cells, and the roles of the bone microenvironment in oxidative stress regulation in dormant myeloma.

Sensitivity of proliferating myeloma cells to ROS inducer auranofin and a novel 'dormalytic' and rescue with antioxidant N-acetyl cysteine (NAC) was tested using resazurin dose-response assays. To investigate dormant cells, Vybrant$^{TM}$ DiD labelled myeloma cells were plated in monoculture or co-cultured with osteoblastic lineage cells (MC3T3) to support dormancy, with dormant cells identified by DiD retention. Apoptosis, ROS levels and oxidative damage markers (8-oxo-G, 4-HNE, nitrotyrosine) were assessed by flow cytometry and immunofluorescence microscopy.

Viability of myeloma cell lines (U266, JJN3, 5TGM1) was reduced by the dormalytic or auranofin, and rescued with NAC pretreatment (4mM, both drugs p<1e-15 in U266). Apoptosis was induced and viability decreased by the dormalytic (1µM, p<0.0001) and auranofin (0.4µM, p=0.0059) in monocultured dormant (DiD+++) U266 cells, with significant NAC rescue (dormalytic p=0.0028, auranofin p<0.0001). Treatments also induced oxidative stress markers. However, dormant cells were less sensitive to the dormalytic (4.8-fold) and auranofin (1.3-fold) when co-cultured with osteoblasts compared to monoculture, suggesting osteoblasts protect dormant cells from oxidative stress. NAC treatment in co-culture provided significant rescue (48.6% viability increase) from the dormalytic and near full reversal of auranofin toxicity, providing further evidence of osteoblast induced therapeutic resistance to oxidative stress in dormant myeloma cells.

This study highlights ROS induction as a potential novel therapeutic strategy for dormant myeloma cells and shows direct influence of osteoblasts on oxidative stress regulation, suggesting both a dormancy support and therapeutic resistance mechanism in the bone microenvironment. Therefore, dormalytics are promising for killing dormant cells to increase survival of myeloma patients, and full eradication may require dual targeting of microenvironment cells.

### P23

### Developing Patient-Derived 3D Tumour Explants and Cell Cultures as Models to Study Osteosarcoma

Georgina Wherry[1,2], Christopher Pinder[1], Dinethri Patel[1], Anna Tollit[3], Fatine Oumlil[3], Nina Melzer[3], Justas Stanislovas[1], Adrienne Flanagan[1,3], Sandra Strauss[1], Lucia Cottone[1]

[1]University College London, London, UK. [2]Queen Mary University of London, London, UK. [3]Royal National Orthopaedic Hospital, London, UK

### Abstract

INTRODUCTION:

Osteosarcoma (OS) is the most common primary sarcoma of the bone in children, adolescents and young adults[1]. Standard treatment currently consists of a regimen of neoadjuvant chemotherapy followed by tumour resection and further adjuvant chemotherapy[2]. Treatment options, however, have remained largely unchanged for the last 50 years despite poor survival rates[2]. A significant portion of OS research is carried out on commercially available cell lines, which provide ease of use but have largely diverged from the original source material over decades of passaging, or *in vivo* mouse models, which whilst helpful in recapitulating the disease within an organism, do not accurately represent the evolution of disease in humans. To better understand OS and ultimately improve treatment options and outcomes, representative *in vitro* models that more closely resemble the disease in patients are needed.

METHODS:

As part of a wider collaborative effort, we have collected tissue from 55 patients diagnosed with OS with associated histological and clinical data. 26 samples were digested with collagenase II to derive 2D primary cell cultures and selected cultures were treated for 7 days with different doses of chemotherapeutic agents commonly used in the treatment of OS (doxorubicin, cisplatin and methotrexate). Additionally, 42 samples were dissected into tumour explants[3] (1mm$^3$ fragments of tissue) which were cultured *ex vivo* each in 1 well of a 96-well plate and treated for up to 72 hours with doxorubicin.

RESULTS:

We have established 19 new primary cell cultures retaining the genomic and transcriptomic landscape of the tumour of origin, with selected cultures showing sensitivity to chemotherapeutic agents in a dose-dependent manner. Concurrently, we have worked towards optimising the culture of OS tumour-derived explants with the fragments retaining the tumour architecture and microenvironment. Using H&E staining we show that the structure of the tumour explants is maintained up to 72 hours in culture, serving as a short-term 3D tumour model. Moreover, we show decreased cellular viability of the explants upon treatment with doxorubicin.

CONCLUSIONS:

Collectively, our data show the establishment of newly developed patient-relevant 2D and 3D models derived from fresh patient material which can be used as platforms to study treatment response in OS and provide the basis for more complex 3D model development.

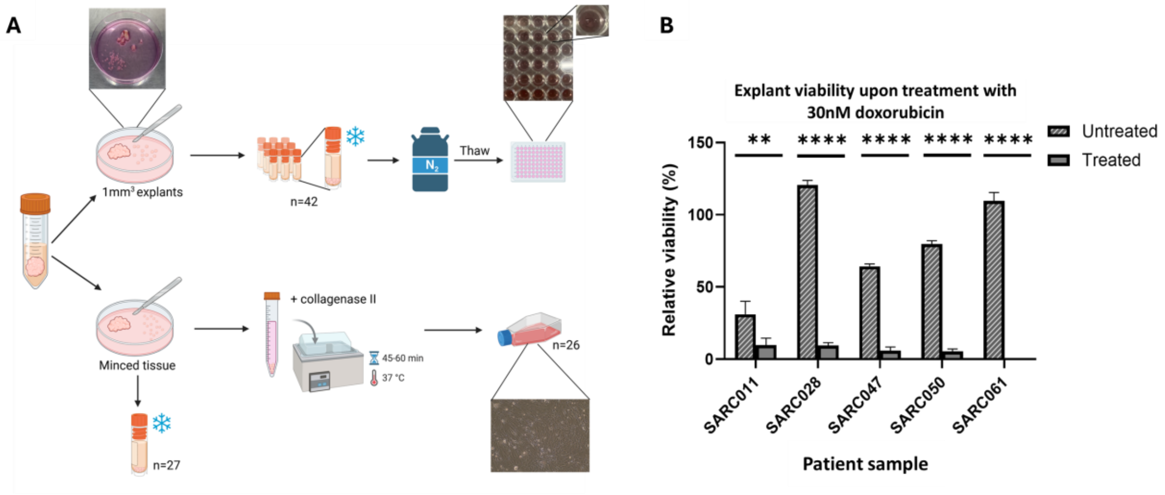

**Figure 1: (A)** Methods of processing tumour tissue for explants or 2D cell cultures. **(B)** Relative viability of explants from 5 patient samples after 48 hours of culture either in basal conditions (untreated) or treated with 30nM doxorubicin. Error bars represent 4 technical repeats of pooled supernatants from 5 explants per condition. Viability calculated as a percentage of PrestoBlue™ fluorescence measurement at 0h timepoint (**P>0.001, ****P>0.00001).

References

1. Nie, Z. et al. (2018). *Oncol Lett*. 16(5):6502-6514.
2. Beird, H.C. et al. (2022). *Nat Rev Dis Primers*. 8(1):77.
3. Roelofsen, L.M. et al. (2023). *STAR Protoc*. 4(2):102282.

### P24

### Investigating Breast Cancer–driven Reprogramming of the Osteogenic Lineage during Bone Metastasis

Soamopriya Som[1], Oliver Pearce[2], Stefaan Verbruggen[1]

[1]Centre for Bioengineering, School of Engineering and Materials Science, Queen Mary University of London, London, UK. [2]Barts Cancer Institute, Faculty of Medicine and Dentistry, Queen Mary University of London, London, UK, London, UK

### Abstract

### Introduction

Bone is the most common site for metastasis in breast cancer [1], resulting in the development of expanding lesions that degrade and weaken bone tissue, resulting in complex fractures and severe bone pain in 75% of patients [2]. However, despite much research, the mechanisms underlying pre-metastatic niche formation remain incompletely understood. Furthermore, previous research in this area has predominantly focused on osteoclast-mediated bone destruction. In contrast, less is known about how cells within the osteogenic compartment, in particular osteoblasts and osteocytes, contribute to tumour colonisation of bone.

The aim of this study was to examine the molecular mechanisms through which breast cancer cells regulate osteogenic niche cell phenotypes and how these changes contribute to tumour-supportive remodelling of the bone microenvironment.

### Materials and Methods

MLO-Y4 osteocyte-like cells, MC3T3-E1 osteoblasts and C3H10T1/2 mesenchymal stem cells (MSCs) were used as robust osteogenic cell lines. 4T1, E0771, and HC11 cell lines were used to model different breast cancer subtypes and effects of non-tumourigenic cells. Conditioned media experiments were conducted to investigate whether metastatic priming of cells in the osteogenic niche may occur. Pathways of interest were identified through analysis of bulk RNA-sequencing and data from a breast cancer patient cohort that developed bone metastases, publicly available on GEO (GSE2034) [3].

### Results

In silico analysis revealed upregulation of pathways associated with tumour invasion, extracellular matrix remodelling and interleukin-mediated signalling (Figure 1A). Consistent with this, osteocytes and osteoblasts treated with breast cancer conditioned media exhibited increased expression of extracellular matrix genes, including COL1A1, FN1 and POSTN. Upregulation of TGF-β-associated signalling components was also observed (Figure 1B), in keeping with our previous findings that a TGF-β-mediated feedback loop may spur tumour development [4].

### Discussion

These preliminary findings identify candidate signalling pathways that may drive early breast cancer metastasis to bone, including JAK/STAT6 and TGF-β, suggesting the potential presence of a 'cancer-associated' osteogenic niche phenotype. Ongoing work aims to further characterise tumour-driven reprogramming of the osteogenic niche and determine how these changes promote tumour colonisation and therapeutic resistance.

### References

1R.E. Coleman (2001) Cancer treatment reviews. 27(3):165-76

2Coleman R, Smith P, Rubens R. Clinical Course and Prognostic Factors Following Bone Recurrence from Breast Cancer. Vol 77.; 1998.

3 Wang Y, Klijn JG, Zhang Y, Sieuwerts AM et al. Gene-expression profiles to predict distant metastasis of lymph-node-negative primary breast cancer. Lancet 2005 Feb 19-25;365(9460):671-9

4 Verbruggen, S.W. et al. (2023) 'A novel primary cilium-mediated mechanism through which osteocytes regulate metastatic behavior of both breast and prostate cancer cells', Advanced Science, 11(2)

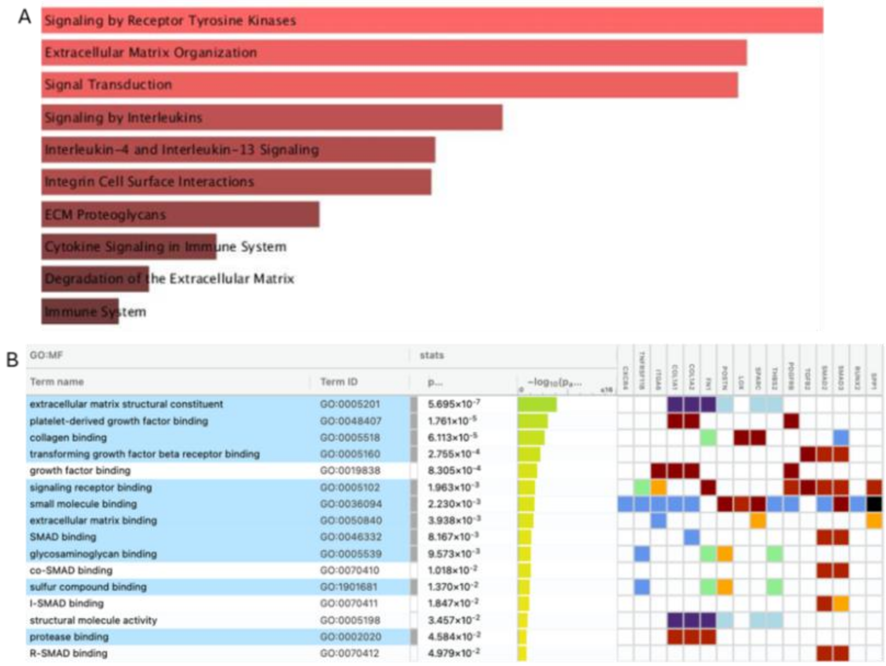

**Figure 1. Pathways involved in breast cancer remodelling of bone microenvironment**
(A) Bone relapse enriched pathways identified from dataset GSE2034 and mapped to Reactome 2024 pathway library. Reactome pathways were ranked by –log10(p-value). (B) GO molecular function enrichment for genes identified as overexpressed in 'cancer-associated' osteocytes and osteoblasts

### P25

Abstract Withdrawn

**P26**

Abstract Withdrawn

### P27

### DNMT3/1 Enzyme Inhibition as a Rejuvenation Strategy for Aged Skeletal Stem Cells

Izzy Matthews[1], Chao Jiang[2], Udo Oppermann[2], Emma Siragher[3], Courtney Hanna[3], Isabel Orriss[1], Ravenska Wagey[4], Scott J Roberts[1]

[1]Royal Veterinary College, London, UK. [2]University of Oxford, Oxford, UK. [3]University of Cambridge, Cambridge, UK. [4]STEMCELL Technologies, Vancouver, Canada

### Abstract

Age-related functional decline of skeletal stem cells (SSCs) is marked by diminished proliferation/differentiation potential. *In vivo* this contributes to skeletal dysfunction, and *in vitro* limits cell therapy applications. Epigenetic drift is a major molecular driver of this decline.

shRNA knock-down of epigenetic targets recovered proliferation in aged human mesenchymal stromal cells (hMSCs). Small-molecule inhibitors of these targets were tested on aged human periosteum-derived stem cells (hPDCs) (P23:P5, osteogenesis; fold change (fc)=0.27, p=0.0002, chondrogenesis; fc=0.48, p<0.0001, proliferation; fc=0.65, p<0.0001). Utilising a reduced factorial design, DNA methyltransferase 3/1 inhibition (DNMTi) was identified as a promising intervention.

DNMTi during expansion reduced alkaline phosphatase (ALP) activity (pNpp; DNMTi:control fc=0.473, p<0.0001) and induced subcellular morphological change (cells/field of view; fc=6.63, p=0.0003). Transient DNMTi (trans-DNMTi) revealed the impact on morphology was reversible, and had a lasting positive impact on phenotype, indicated by increased refractive index (RI; trans-DNMTi:control fc=2.18, p=0.0002). Cytotoxicity was determined by DNA analysis (ng/mL; DNMTi:control fc=1.25, p=0.007), with metabolic analyses indicating recovery of drug-induced stress (LDH release; trans-DNMTi:control fc=1.4, p=0.34 (ns), DNMTi:control fc=2.2, p=0.0038).

Pre-exposure (trans-DNMTi or continued (cont-DNMTi)) resulted in increased proliferative capacity (ng/mL; cont-DNMTi:control fc=2.57, p=0.041), and osteochondral potential (osteogenic; cont-DNMTi:control fc=5.22, p<0.0001, trans-DNMTi:control, fc=4.64, p<0.0001 (Alizarin Red/ARS), chondrogenic; trans-DNMTi:control fc=1.08, p=0.038 (Alcian Blue/AB). RNAseq revealed a recycling/metabolic signature consistent with cellular reset/rejuvenation, enriched for ribosomal, lysosomal, proteasomal and oxidative phosphorylation activity (fold enrichment 11, 6.8, 10.3, 6.9 (trans-DNMTi:control), and 5.9, 5.8, 8.4, 6 (cont-DNMTi:control) respectively). Pre-exposure also improved chondrogenic and proliferative potential in both aged hMSCs and murine PDCs (mPDCs), indicating efficacy across niches and species (cont-DNMTi:control, AB; fc=1.14, p=0.0015 (mPDC), fc=1.33, p=0.0025 (hMSC), DNA concentration; fc=7.8, p<0.001 (mPDC), fc=1.5, p=0.0002 (hMSC)).

DNMT3a- and DNMT1-specific inhibitors (DNMT3ai and DNMT1i; 1µM) displayed the same trend towards ALP modulation as DNMTi but without reaching significance, indicating the importance of dual targeting of DNMT3/1 for rejuvenation (pNpp; DNMT3ai:control fc=0.93, p=0.99; DNMT1i:control fc=0.7, p=0.255).

To further define DNMT function in skeletogenesis, µCT was carried out on E18.5 wild-type (WT) and *Dnmt3b* conditional knockout (cKO, Sox2-Cre) mouse embryos, revealing reduced bone volume ($\mu m^3$; cKO:WT fc=0.7, p=0.039 (whole-embryo), fc=0.65, p=0.001 (femur)) (Figure 1). These data indicate the importance of DNMT3b in embryonic bone formation, consistent with the notion of DNMT involvement in tissue maturation.

This study reports a novel rejuvenation strategy for aged SSCs via DNMT3/1 modulation. Abrogating the functional decline of SSCs associated with ageing holds potential for regenerative medicine and skeletal repair.

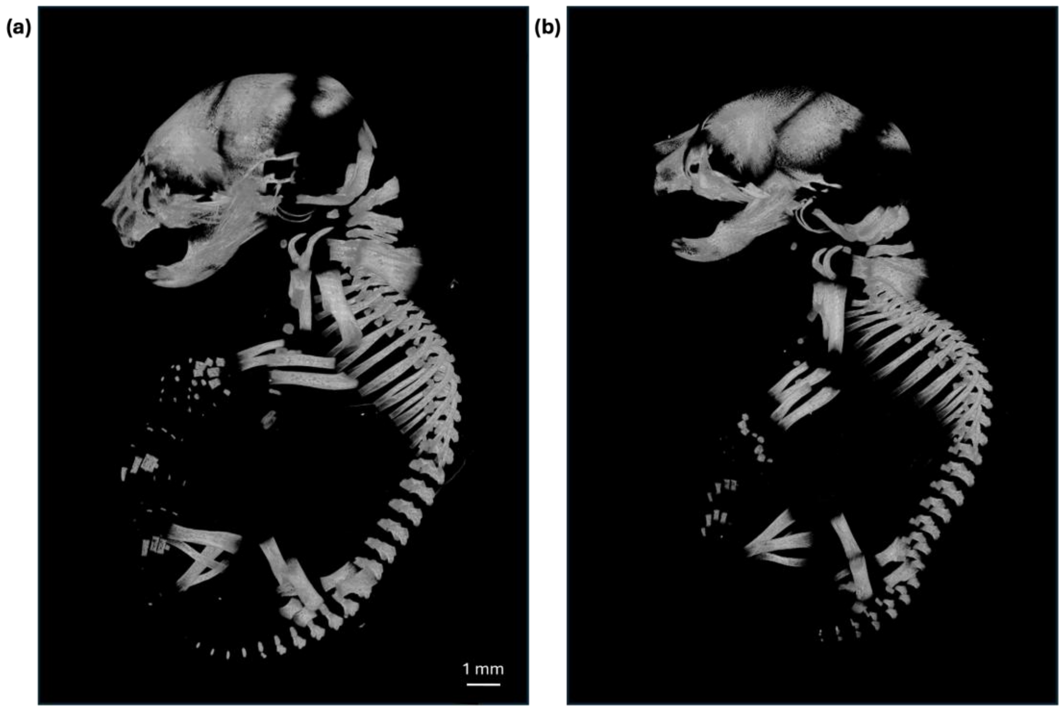

**Figure 1. Conditional *DNMT3b* knockout impairs skeletogenesis *in utero*.** Representative µCT images of wild-type (a) and *Dnmt3b* cKO (Sox2-Cre) (b) E18.5 mouse embryos. Scale bar applies to both images, 1 mm.

### P28

### Investigating Novel Mechanisms Underpinning Inhibition of Bone Mineralisation by Uremic Toxins

Lucie Bourne[1], Sebastian Koci-Edwards[1], Sahil Day[1], Worachet Promruk[2], Colin Farquharson[2], Katherine Staines[1]

[1]University of Brighton, Brighton, UK. [2]Roslin Institute, University of Edinburgh, Edinburgh, UK

### Abstract

Chronic Kidney Disease–Mineral and Bone Disorder (CKD-MBD) is a growing global health concern driven by the rising prevalence of diabetes, hypertension, and an ageing population. CKD-MBD leads to secondary hyperparathyroidism, hyperphosphatemia, and elevated FGF23, and this altered systemic milieu compromises bone formation while promoting ectopic calcification. The aim of this study was to investigate the effects of the uremic toxin indoxyl sulfate (IS) on osteoblast mineralisation and identify new signalling targets for CKD-MBD.

Primary calvarial osteoblasts were isolated from 3–5-day old mice and cultured for ≤21 days in Alpha Minimum Essential Medium supplemented with 10% FBS, 100 U/ml penicillin, 100 µg/ml streptomycin, 2 mM β-glycerophosphate and 50 µg/ml L-ascorbic acid. At day 4, cells were treated with 2 µM IS and the effects on mineralised bone nodule formation (days 14 & 21) and gene expression (days 7,14, 21) were assessed.

IS significantly reduced bone nodule formation by ≤88% at both early (day 14) and late (day 21) stages of mineralisation ($p<0.001$). After 21 days of culture, this was associated with the reduced expression of *Sost* and *Phex* by 8.3 and 3.8-fold respectively ($p<0.001$), yet *E11* and *Dmp1* expression were unchanged. Despite this, osteoblast marker expression (*Bglap, Alpl, Spp1, Rankl, Opg, Col1a1*) were unchanged during the proliferation and maturation stages of culture (days 7 & 14). To investigate novel pathways underpinning the observed effects on matrix mineralisation, a PCR array targeting oxidative stress and antioxidant defence pathways was performed at day 14. This identified differential expression of 45 genes significantly upregulated, and 4 genes significantly downregulated with IS treatment, many of which were associated with the nuclear factor erythroid 2-related factor 2 (Nrf2) pathway. To target this pathway, osteoblasts were treated with sulforaphane (1 & 5 µM) or betaine (3, 10, 30 mM) – both natural activators of the Nrf2 pathway. However, neither of these were able to rescue the IS-mediated inhibition of mineralisation nor alter mineralisation when cultured alone.

Taken together, these data suggest that in osteoblasts, the uremic toxin IS impairs mineralisation, possibly through inhibition of the Nrf2 pathway and is related to altered osteocyte differentiation. Ongoing experiments will further interrogate this pathway to identify potential novel therapeutic targets for CKD-MBD.

### P29

Abstract Withdrawn

### P30

### EZH2 Inhibition Reveals an Epigenetic Basis for Osteocyte Mineralisation

Sejal Kapoor[1], Rebecca McIntyre[2], Andrew A Pitsillides[1], Scott J Roberts[1]

[1]Royal Veterinary College, London, UK. [2]Relation Therapeutics, London, UK

### Abstract

The MLOA5 pre-osteocyte cell line engages in rapid and extensive matrix mineralisation and is widely reported to express negligible levels of the osteocyte marker and Wnt antagonist Sclerostin. Wnt signalling is critical for bone homeostasis and has roles in osteoblast differentiation/mineralisation and osteocyte formation. As mature osteocytes must restrict mineralisation and Sclerostin is epigenetically regulated we hypothesise that the MLOA5 hyper-mineralisation phenotype is due to unchecked Wnt pathway activity through epigenetic blockade of natural Wnt antagonists, including Sclerostin (*Sost*). To address this, we aim to identify enzymes that exert an epigenetic control of mineralisation through Wnt pathway modulation. Understanding how DNA modifications induce alterations in bone tissue homeostasis may lead to novel therapeutics for tissue regeneration.

MLOA5 cells cultured in monolayer, seeded at 8000 cells/$cm^2$ were differentiated for 8 days in standard osteogenic induction media along with DMSO or with inhibitors of epigenetic enzymes (n=3/condition). To quantify mineralisation, cells were fixed, stained with 1% Alizarin Red and leached, with absorbance measured at 570nm. Cell viability was measured by a resazurin-based metabolic assay. RNA was extracted and cDNA prepared prior for qRT-PCR. Gene expression was calculated relative to the average expression of housekeeper genes (*Tbp*, *Hprt1*) via the delta-Ct method

Ten epigenetic enzymes reported to block *Sost* expression/Wnt signalling were selected from the literature. Inhibitors of each enzyme reduced MLOA5 mineralisation (1µM, p<0.001) suggesting epigenetic control of matrix mineralisation. EZH2 inhibitor, EPZ6438, showed the greatest reduction in mineralisation (p<0001) and was further tested at 5 ascending concentrations (Figure1). The greatest anti-mineralisation activity of EPZ6438 (normalised to cell viability) was observed at 100nM (fc=1.37, p<0.001) and mineralisation tended to increase with reducing concentration. A reduction in endpoint (8 days) *Axin2* gene expression (Fc=1.30, p<0.05) was consistent with Wnt pathway inhibition. Unexpectedly, a significant reduction in *Sost* expression was also observed (Fc = 4.43, p<0.05), along with no change in *Runx2,* which may be due to the cells reaching homeostasis by day 8. Early time-point analysis at 24 hours revealed no change in *Sost* expression, however, an upregulation in *Sfrp4* expression (fc=1.39, p<0.005) was observed suggesting that EZH2 may restrict osteocyte mineralisation via Wnt antagonists other than *Sost*.

A novel interaction between EZH2 and Sfrp4 pinpoints a node in the Wnt pathway that could be harnessed as a therapeutic in bone diseases. Longitudinal transcriptomic analysis and multi-gene profiling would be required to test this hypothesis and further understand the control of osteocyte biology by EZH2.

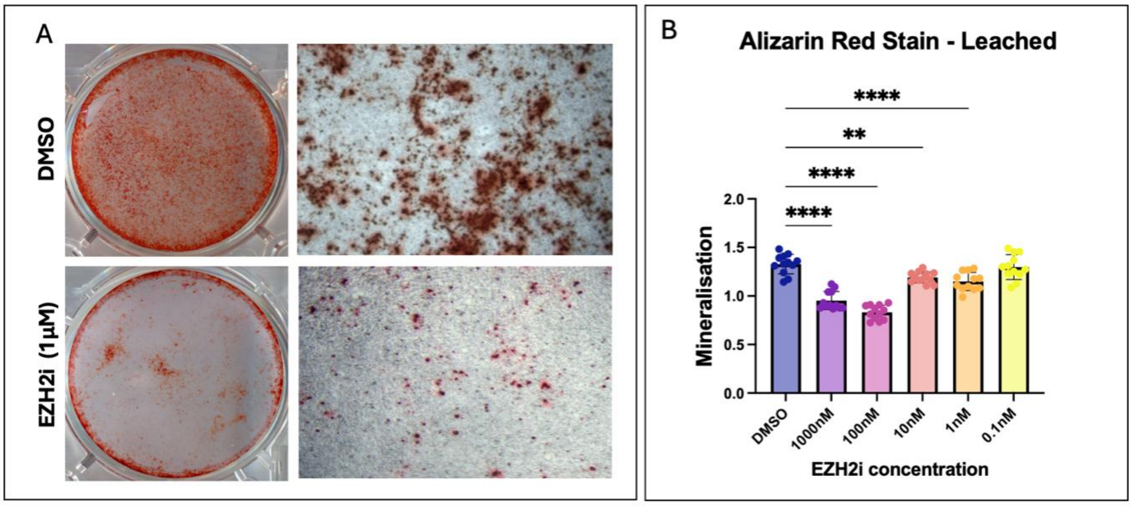

**Figure 1. EZH2 inhibitor (EZH2i) causes a reduction in mineralisation in MLOA5 cells**

A) Representative well & light microscope images of MLOA5 cells stained with Alizarin Red (AR) at day 8.

B) Quantification of AR stain showing effect of EZH2i on MLOA5 cell mineralization after 8 days. Assays were performed using three technical replicates (*n* = 3-6/group). Statistical comparisons: $^{**}P \leq 0.01$, $^{***}P \leq 0.001$ and $^{****}P \leq 0.0001$

### P31

### Multiple Vertebral Fractures and Spinal Fixation Failure Following Rapid Weight Loss During Tirzepatide Therapy: A Case Report

Chadi Rakieh, Claire Mennan, Diane Powell, Shu Ho, Bernhard Tinns, Birender Balain

The Robert Jones & Agnes Hunt Orthopaedic Hospital NHS Foundation Trust, Oswestry, UK

### Abstract

**Background**
Patients with obesity and type 2 Diabetes may have impaired bone quality despite relatively preserved bone mineral density. Dual incretin receptor agonists such as Tirzepatide are increasingly used for weight management and can induce substantial weight loss over a short period. Rapid weight loss may reduce skeletal loading and potentially exacerbate underlying bone fragility. Conventional assessment using Dual-energy X-ray Absorptiometry (DXA) may not fully capture deterioration in trabecular bone microarchitecture.

**Case Presentation**
We report the case of a 55-year-old man with obesity and long-standing type 2 diabetes with no prior history of fragility fractures. Baseline DXA performed in 2020 demonstrated osteopenia with a femoral neck T-score of −2.0. Lumbar spine bone mineral density could not be assessed due to spinal instrumentation, and trabecular bone score assessment using TBS version 3 was not feasible because body mass index exceeded the validated range. In March 2025, tirzepatide therapy was initiated for weight management. Over the subsequent six months the patient experienced rapid weight loss of approximately 14 kg. In July 2025, while weight loss was ongoing, he underwent revision L1–S1 posterolateral spinal fusion for degenerative spinal disease. Pre-operative imaging confirmed the absence of acute vertebral fractures.

**Clinical Event and Investigations**
Three months after surgery, in October 2025, the patient presented with acute back pain and was found to have instrumentation failure with new vertebral fractures at T12 and L1 requiring vertebroplasty. Secondary causes were excluded and laboratory investigations showed HbA1c 47 mmol/mol, adjusted calcium 2.44 mmol/L, alkaline phosphatase 135 U/L, parathyroid hormone 8.1 pmol/L, and 25-hydroxyvitamin D 51 nmol/L. Repeat DXA demonstrated persistent osteopenia at the femoral neck (T-score −2.1). However, further assessment using Quantitative Computed Tomography revealed severe trabecular bone deficit with a volumetric trabecular T-score of −5.08 (Figure 1).

**Conclusion**
This case demonstrates marked discordance between DXA and QCT in assessing skeletal fragility in a patient with metabolic risk factors for impaired bone quality. Vertebral fractures and spinal fixation failure occurred temporally following rapid weight loss during six months of tirzepatide therapy, although causality cannot be established. The inability to assess lumbar spine BMD and trabecular bone score using conventional methods further highlights the diagnostic challenges in such patients. Advanced assessment of bone quality may be considered in selected individuals undergoing rapid pharmacological weight loss or major orthopaedic procedures.

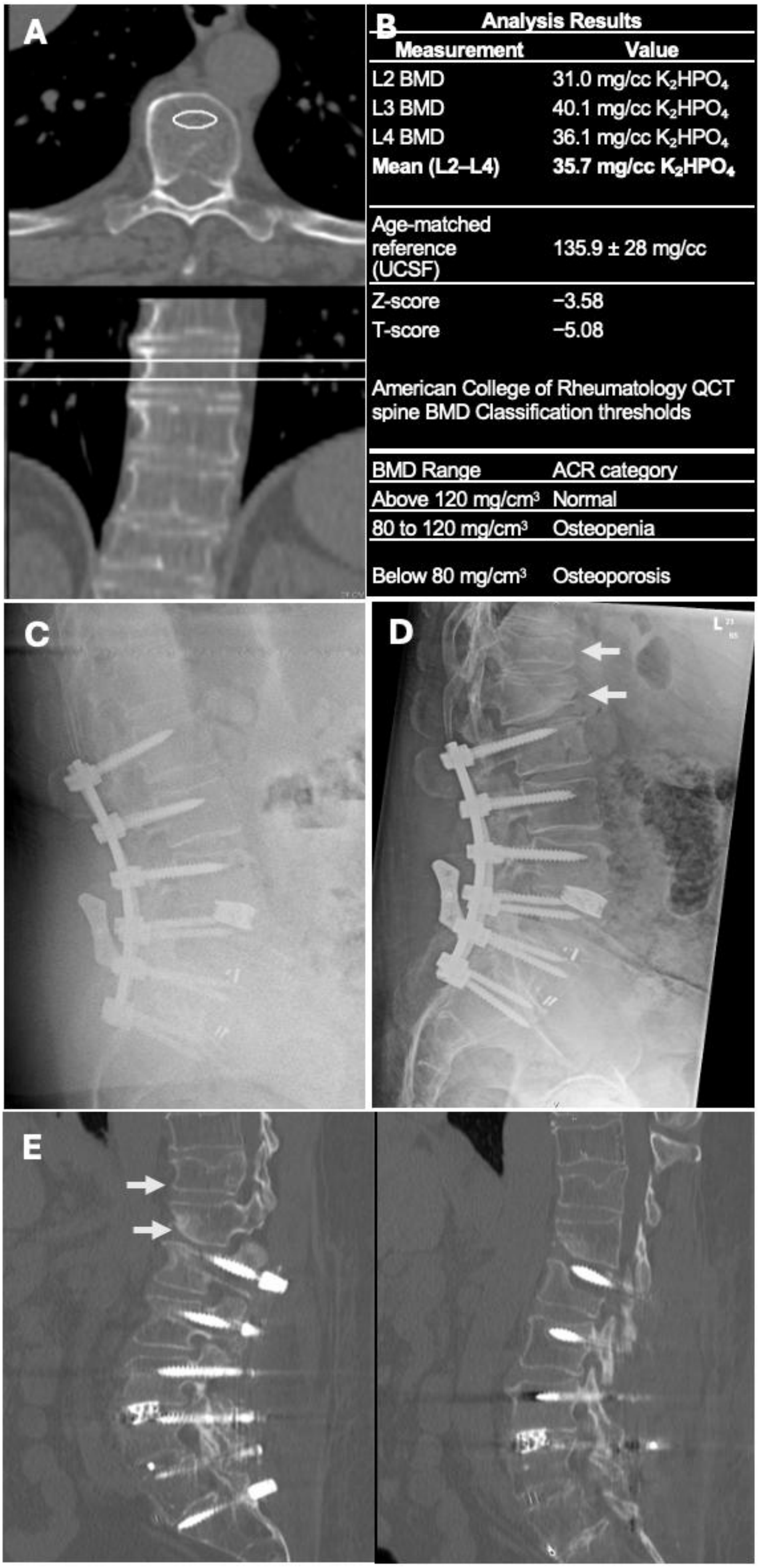

**Analysis Results**

| Measurement | Value |
|---|---|
| L2 BMD | 31.0 mg/cc $K_2HPO_4$ |
| L3 BMD | 40.1 mg/cc $K_2HPO_4$ |
| L4 BMD | 36.1 mg/cc $K_2HPO_4$ |
| **Mean (L2–L4)** | **35.7 mg/cc $K_2HPO_4$** |
| Age-matched reference (UCSF) | 135.9 ± 28 mg/cc |
| Z-score | −3.58 |
| T-score | −5.08 |

American College of Rheumatology QCT spine BMD Classification thresholds

| BMD Range | ACR category |
|---|---|
| Above 120 mg/cm³ | Normal |
| 80 to 120 mg/cm³ | Osteopenia |
| Below 80 mg/cm³ | Osteoporosis |

**Figure 1**. Imaging demonstrating severe trabecular bone deficit and subsequent vertebral fracture with instrumentation failure.
(A) Quantitative computed tomography (QCT) images of the thoracolumbar spine showing the region of interest used for trabecular bone analysis, including an axial slice of the vertebral body and a coronal reconstruction indicating the analysed vertebral level.
(B) QCT analysis demonstrating markedly reduced trabecular volumetric bone mineral density (mean 35.7 mg/cm$^3$) with a trabecular T-score of −5.08, indicating severe trabecular bone deficit despite only osteopenia on dual-energy X-ray absorptiometry (DXA).
(C) Lateral lumbar radiograph obtained immediately following revision L1–S1 posterolateral spinal fusion (10 July 2025).
(D) Follow-up lateral radiograph three months post-operatively (October 2025) demonstrating new vertebral fractures at T11–T12 (arrows) with associated failure of the proximal aspect of the instrumentation.
(E) Sagittal CT reconstructions three months post-operatively (October 2025) demonstrating vertebral fractures at T11–T12 (arrows) with instrumentation failure, including screw loosening at L1–L2 and breach of the L1 superior endplate with extension into the adjacent intervertebral disc.

### P32

### A Cross-Sectional Study of DXA-Derived Chondrocalcinosis and its Associations with Biochemical Findings and Musculoskeletal Outcomes in 62,597 UK Biobank Participants

Jason da Costa[1], Rian O'Malley[1], James Pilgrim[1], Mijin Jung[1,2], Yahong Wu[3], Justin Boer[4], Cindy Boer[3], Jon Tobias[1,2], Ben Faber[1,2]

[1]Musculoskeletal Research Unit, University of Bristol, Bristol, UK. [2]MRC Integrative Epidemiology Unit, University of Bristol, Bristol, UK. [3]Department of Internal Medicine, Erasmus MC, University Medical Centre, Rotterdam, Netherlands. [4]Department of Radiology, Erasmus MC, University Medical Centre, Rotterdam, Netherlands

### Abstract

**Purpose:** Chondrocalcinosis represents abnormal calcium-crystal deposition within joint spaces on imaging. Although linked to osteoarthritis are established, underlying mechanisms, including the relationship with serum calcium, and clinical implications remain uncertain. Furthermore, population-level evidence on differences in clinical outcomes, related to sex and severity of chondrocalcinosis is limited. Typically identified on radiographs, chondrocalcinosis is also visible on high-resolution Dual-energy X-ray Absorptiometry (DXA) scans, available in UK Biobank (UKB). This study quantified DXA-derived chondrocalcinosis, and evaluated associations with musculoskeletal outcomes, including sex differences, and biochemical results.

**Methods:** UKB knee DXA scans were analysed for chondrocalcinosis. Participants with bilateral scans and complete covariate data were included. Raters semi-quantitatively graded chondrocalcinosis (0–3) in medial and lateral compartments of both knees and summed to generate a total chondrocalcinosis score (0–12). Participants were stratified in severity as mild (1–2), moderate (3–5), or severe (6–12) (Figure 1A). Outcomes included prolonged knee pain (>3 months), hospital-diagnosed knee osteoarthritis, and subsequent total knee replacements (TKR). Pain was self-reported; osteoarthritis and TKR were derived from linked healthcare records; serum calcium, phosphate and alkaline phosphatase were obtained at baseline. Logistic regression compared chondrocalcinosis severity with participants without chondrocalcinosis, controlling for: age, height, weight, and ethnicity. Sex-stratified analyses were performed.

**Results:** In a cohort of 62,597 participants (mean age 65.2 years; 52.1% female), 2,443 cases were identified with similar prevalence between sexes (F:3.8%, M:4.0%). Inter-rater reliability was high (kappa: medial 0.78; lateral 0.75). Chondrocalcinosis demonstrated progressive associations with knee pain (mild OR 1.62 [95% CI 1.40–1.87], severe 1.92 [1.56–2.38]), knee osteoarthritis (mild 1.82 [1.44–2.30], severe 1.94 [1.38–2.73]) and TKR (mild 1.26 [0.72–2.21], severe 2.32 [1.22–4.39]) (Figure 1B). Per 1-SD increase, serum calcium (1.18 [1.13-1.23]) and alkaline phosphatase (1.06 [1.02-1.11]) were positively associated with the presence of chondrocalcinosis. Males showed stronger associations with clinical outcomes (Figure 1C).

**Conclusions:** This is the largest study evaluating associations between chondrocalcinosis and musculoskeletal outcomes. Chondrocalcinosis severity showed progressive associations with prolonged knee pain and osteoarthritis. This is the first study demonstrating association between increased serum calcium and chondrocalcinosis, implying a potential causal link. Therefore, it seems advisable to avoid calcium supplementation in those individuals with chondrocalcinosis and normal serum calcium levels. The differences in associations between sexes suggests the possibility of sex-specific pathomechanisms. The large UKB sample will facilitate further genetic analyses, increasing our understanding of the biological pathways involved.

| Exposure | Mild chondrocalcinosis | | | | Moderate chondrocalcinosis | | | | Severe chondrocalcinosis | | | |
|---|---|---|---|---|---|---|---|---|---|---|---|---|
| | Unadjusted | | Adjusted | | Unadjusted | | Adjusted | | Unadjusted | | Adjusted | |
| | OR [95% CI] | P | OR [95% CI] | P | OR [95% CI] | P | OR [95% CI] | P | OR [95% CI] | P | OR [95% CI] | P |
| Combined | | | | | | | | | | | | |
| Serum alkaline phosphatase | 1.10 [1.04 - 1.17] | $1.23\times10^{-03}$ | 1.04 [0.98 - 1.11] | 0.21 | 1.15 [1.07 - 1.22] | $4.40\times10^{-05}$ | 1.07 [1.00 - 1.15] | 0.05 | 1.15 [1.06 - 1.26] | $8.68\times10^{-04}$ | 1.08 [0.99 - 1.19] | 0.09 |
| Serum Calcium | 1.11 [1.05 - 1.19] | $8.03\times10^{-04}$ | 1.10 [1.03 - 1.17] | $3.02\times10^{-03}$ | 1.18 [1.10 - 1.27] | $4.41\times10^{-06}$ | 1.17 [1.09 - 1.26] | $1.10\times10^{-05}$ | 1.35 [1.24 - 1.48] | $2.84\times10^{-11}$ | 1.35 [1.24 - 1.48] | $3.99\times10^{-11}$ |
| Serum Phosphate | 0.97 [0.91 - 1.03] | 0.36 | 0.96 [0.90 - 1.03] | 0.27 | 0.90 [0.84 - 0.97] | $7.06\times10^{-03}$ | 0.91 [0.84 - 0.98] | 0.01 | 0.97 [0.88 - 1.07] | 0.52 | 0.98 [0.89 - 1.09] | 0.74 |
| Female | | | | | | | | | | | | |
| Serum alkaline phosphatase | 1.15 [1.07 - 1.24] | $1.19\times10^{-04}$ | 1.06 [0.98 - 1.15] | 0.18 | 1.19 [1.10 - 1.30] | $4.27\times10^{-05}$ | 1.04 [0.94 - 1.15] | 0.50 | 1.25 [1.12 - 1.38] | $3.25\times10^{-05}$ | 1.08 [0.95 - 1.23] | 0.22 |
| Serum Calcium | 1.19 [1.10 - 1.29] | $2.47\times10^{-05}$ | 1.13 [1.04 - 1.18] | $4.48\times10^{-03}$ | 1.33 [1.21 - 1.46] | $5.86\times10^{-09}$ | 1.20 [1.09 - 1.33] | $2.17\times10^{-04}$ | 1.54 [1.37 - 1.74] | $2.79\times10^{-13}$ | 1.39 [1.23 - 1.57] | $7.02\times10^{-08}$ |
| Serum Phosphate | 1.00 [0.91 - 1.09] | 0.93 | 0.95 [0.86 - 1.04] | 0.26 | 0.93 [0.83 - 1.05] | 0.24 | 0.83 [0.74 - 0.94] | $2.69\times10^{-03}$ | 0.95 [0.82 - 1.11] | 0.52 | 0.84 [0.72 - 0.98] | 0.03 |
| Male | | | | | | | | | | | | |
| Serum alkaline phosphatase | 1.01 [0.91 - 1.12] | 0.83 | 1.00 [0.90 - 1.11] | 0.97 | 1.09 [0.98 - 1.21] | 0.10 | 1.09 [0.98 - 1.21] | 0.11 | 1.04 [0.90 - 1.19] | 0.62 | 1.04 [0.90 - 1.20] | 0.58 |
| Serum Calcium | 1.00 [0.90 - 1.11] | 0.99 | 1.05 [0.95 - 1.16] | 0.39 | 1.04 [0.94 - 1.16] | 0.46 | 1.10 [0.99 - 1.23] | 0.07 | 1.17 [1.02 - 1.34] | 0.03 | 1.25 [1.09 - 1.43] | $1.29\times10^{-03}$ |
| Serum Phosphate | 0.91 [0.83 - 1.00] | 0.05 | 0.93 [0.85 - 1.02] | 0.14 | 0.91 [0.82 - 1.00] | 0.05 | 0.93 [0.84 - 1.03] | 0.16 | 1.02 [0.89 - 1.16] | 0.76 | 1.05 [0.92 - 1.21] | 0.45 |

**Table 1.** Unadjusted and adjusted associations of serum calcium, phosphate, and alkaline phosphatase with severity of chondrocalcinosis

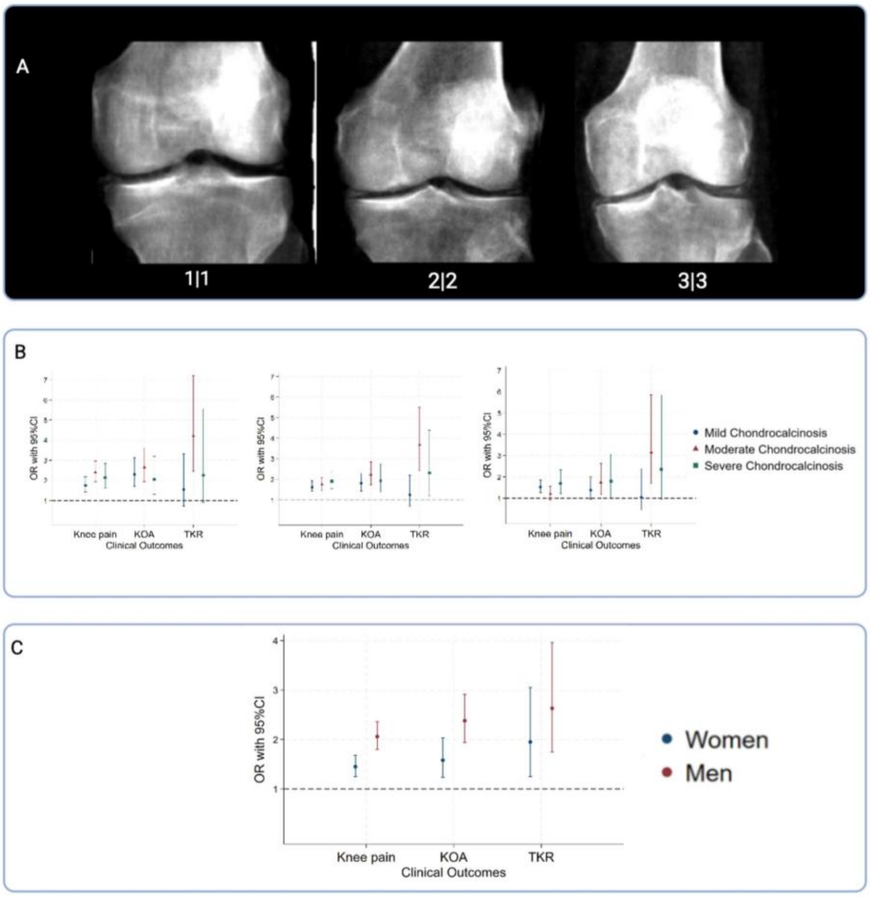

**Figures 1.** A – DXA Scans from UK Biobank showing different severities of chondrocalcinosis with assigned gradings. Left image: grade one in medial and lateral compartments. Middle image: grade two in medial and lateral compartments. Right image: grade three in medial and lateral compartments. B – Associations between mild, moderate and severe chondrocalcinosis and clinical outcomes in combined sex (left), male (middle) and female (right) analyses. C – Sex stratified analyses of associations between binary presence of chondrocalcinosis and clincal outcomes.

### P33

### Potential Long-Term Implications of Tuberculosis (TB) Infection on Bone Mineral Density and Body Composition in Zimbabwean Older Adults

Mícheál Ó Breasail[1], Kate Ward[2,3], Tadios Manyanga[4], Hannah Wilson[5], Millie Pearse[2], Anya Burton[5], Lucy Gates[2], Chris Grundy[6], Anthony Muchai Manyara[5], Farirayi Kowo Nyakoko[4], Cynthia Kahari[7], Tafadzwa Madanhire[5], Rashida Ferrand[4,8], Katharina Kranzer[4,8,9], Celia Gregson[5,4]

[1]Department of Medicine, School of Clinical Sciences, Faculty of Medicine, Monash Medical Centre, Nursing and Health Sciences, Monash University, Clayton, Australia. [2]Global Musculoskeletal Health Group, Human Development and Health, University of Southampton, Southampton, UK. [3]MRC Unit, The Gambia @ London School of Hygiene and Tropical Medicine,, Banjul, Gambia. [4]The Health Research Unit Zimbabwe, Biomedical Research and Training Institute, Harare, Zimbabwe. [5]Global Musculoskeletal Research Group, Musculoskeletal Research Unit, Bristol Medical School, University of Bristol., Bristol, UK. [6]MRC International Statistics and Epidemiology Group, London School of Hygiene and Tropical Medicine, London, UK. [7]Department of Oncology, Medical Physics and Imaging Sciences, Faculty of Medicine and Health Sciences, University of Zimbabwe, Harare, Zimbabwe. [8]Clinical Research Department, Faculty of Infectious and Tropical Diseases, London School of Hygiene and Tropical Medicine, London, UK. [9]Division of Infectious Diseases and Tropical Medicine, Ludwig Maximilian University Hospital, Munich, Germany

### Abstract

There are over 150 million tuberculosis (TB) survivors globally, primarily in low-middle-income countries. While acute infection is associated with cachexia and sarcopenia, the implications of TB on bone density and fracture-risk are less well understood. Data from large health registries in high-income countries suggest TB survivors may be at greater risk of osteoporosis and fracture. However, assessment of bone in resource-limited settings, where TB is most prevalent, has largely been neglected, with a paucity of data

This is a secondary analysis from a GIS sampled, population based cross-sectional study (Fractures in sub-Saharan Africa: epidemiology, economic impact and ethnography [Fractures-E$^3$]) of men and women aged ≥40 years conducted in Harare, Zimbabwe. Sociodemographic data, TB and HIV status, fracture history, and anthropometry were collected. Dual-energy X-ray Absorptiometry (DXA) was used to assess total hip (TH) and femoral neck (FN) areal (a)BMD (g/cm$^2$) and whole body (WB) fat mass (kg) from which fat mass index (FMI, kg/m$^2$) was calculated. Peripheral quantitative computed tomography (pQCT) assessed radial and tibial total volumetric BMD (vBMD, mg/cm$^3$), trabecular vBMD (mg/cm$^3$), cross-sectional area (CSA, mm$^2$), compressive bone strength (BSIc), cortical vBMD (mg/cm$^3$), cortical thickness (mm), proximal CSA (mm$^2$), and stress-strain index (SSI, mm$^3$). Age and sex adjusted linear regression determined differences by TB history (Model 1), with further adjustment for HIV status (Model 2), and wealth index and food insecurity (Model 3).

1022 participants, mean age 62.3(14.0) years had DXA and pQCT data (Male:49.3%, TB history:5.5%). People with a TB history were approximately 5 years younger, more likely to be male and live with HIV. No group differences in major osteoporotic fracture history or osteoporosis prevalence were present by TB history. In minimally adjusted models TB history was associated with; lower DXA TH aBMD -7.1%[-11.7%;-2.5%] and FN aBMD -5.7%[-10.0%;-1.4%]; lower radial pQCT total vBMD -6.4%[-11.9%;-1.1%] trabecular vBMD -14.6%[-22.6%;-6.5%] and BSIc -12.4%[-22.0%;-2.7%]; tibial data were similar (Figure). DXA fat mass and FMI were lower by -16.7%[-29.5%;-3.9%] and -16.3%[-28.9%;-3.7%], respectively in those with a history of TB. radial trabecular vBMD remained robust to adjustment (Figure); though DXA difference were attenuated after adjustment. (Figure).

These are the first quantitative bone data from Africa and suggest lasting trabecular compartment deficits in people with a history of TB. Larger prospective studies are needed to understand the underlying mechanisms and possible implications for age-associated fragility fracture in TB survivors particularly in the context of HIV co-infection.

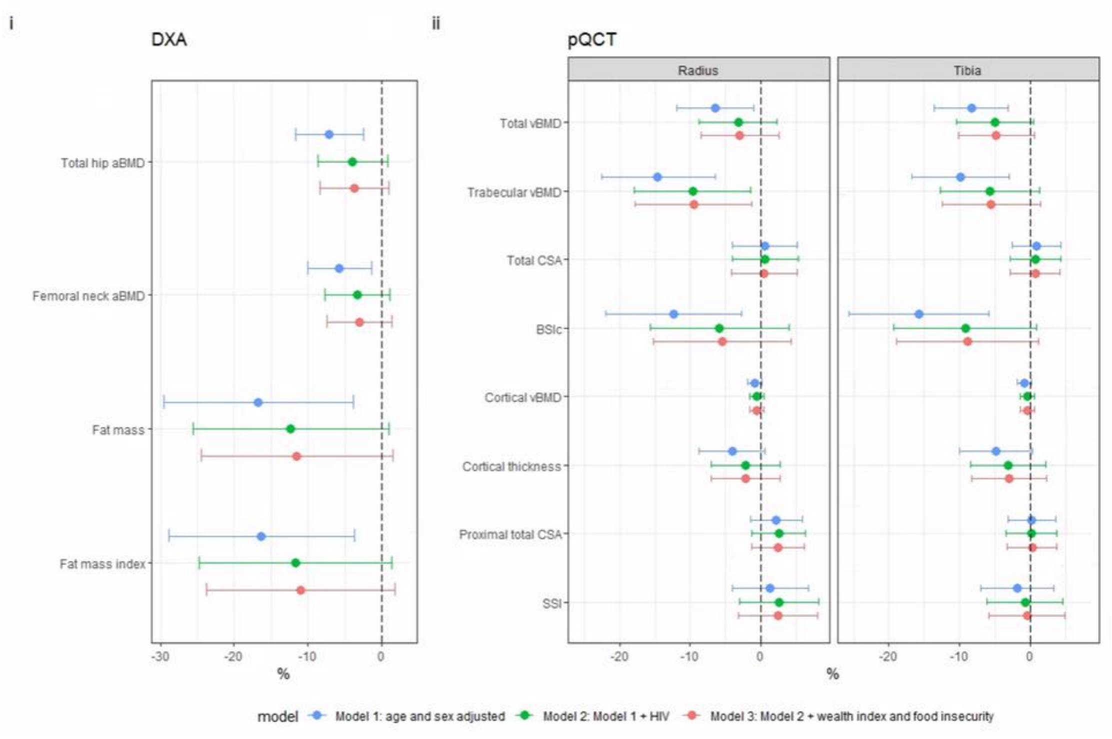

**Figure:** Differences in i) DXA and ii) pQCT measures by TB history expressed as mean[95%CI] symmetric percentage.

### P34

### Receptor Activator of Nuclear Factor Kappa B Ligand Inhibition and Ischaemic stroke: A Systematic Review

Aqsa Anwar, Robin Rumney

University of Southampton, Southampton, UK

### Abstract

**Background:** Receptor activator of nuclear factor kappa B ligand (RANKL) has an established role in osteoclastogenesis, hence pharmacological blockade of RANKL with denosumab is used for the treatment of osteoporosis. RANKL has also been found to be expressed in vascular and neural tissues, where it may be neuroprotective against the effects of ischaemic stroke. Indeed, inhibition of endogenous RANKL by osteoprotegerin (OPG) has been linked to vascular calcification. Thus, RANKL inhibition may be beneficial in controlling osteoporosis but may have detrimental effects upon ischaemic stroke.

**Aims:** The aims of this systematic review were to evaluate associations between RANKL inhibition and ischaemic stroke, specifically: (1) the relationship between OPG and ischaemic stroke risk, severity and prognosis; and (2) whether denosumab therapy in postmenopausal osteoporotic women is associated with ischaemic stroke.

**Methods:** This systematic review was conducted using a predefined PICOS framework. Exposure included denosumab therapy, circulating OPG, or RANKL. Outcomes comprised ischaemic stroke severity, functional outcome, and mortality in stroke cohorts. MEDLINE, Embase, and PubMed were searched for publications. Of 548 records identified, 19 studies were included.

**Results:** Nine studies reported that elevated OPG levels were associated with increased ischaemic stroke risk or related vascular risk factors, with higher rates of cardiovascular events and mortality in older populations. Eight stroke cohort studies found that elevated OPG was linked with increased stroke severity, poorer prognosis, and higher mortality. There was limited evidence of the effects of denosumab from only two studies, with neither identifying a clear directional effect on stroke. Both studies on denosumab were limited by a lack of stroke-specific study design.

**Conclusions:** Elevated OPG levels are strongly associated with a greater burden of cerebrovascular disease and poorer outcomes following ischaemic stroke, while currently available data on the potential effects of denosumab upon ischaemic stroke do not show any similar effect. Although both OPG and Denosumab inhibit RANKL, only OPG is currently associated with poor stroke outcomes and the reasons for this difference between the effects of OPG and denosumab remain unclear. With implications relevant to clinicians, stroke researchers, and guideline developers, this merits further research with adequately powered cohorts and stroke-focussed endpoints.

### P35

### Associations Between Limb Proportionality and Bone and Joint Health in Older Adults

Terence O'Neill[1], Alex Ireland[2]

[1]University of Manchester, Manchester, UK. [2]Manchester Metropolitan University, Manchester, UK

### Abstract

Long bone proportions have been proposed as a sensitive marker of environmental exposures during childhood. In addition, there is some evidence that limb proportionality affects joint biomechanics during movement. Despite this, associations between limb proportionality and bone and joint outcomes, particularly those relevant to osteoporosis, remain little explored.

We examined data in 3,582 participants (1,739 female) aged 60 and over from the National Health and Nutrition Examination Survey (NHANES) third study. Associations between leg length, knee height and femur length relative to height, and crural index as exposures and dual-energy X-ray absorptiometry (DXA)-derived hip BMC, BMC and area, neck-shaft angle, neck length, and hip axis length, and Kellgren-Lawrence (KL) scores of knee osteoarthritis (available in 1,762 individuals) as outcomes were examined with adjustment for relevant covariates, as were sex and age interactions.

Greater leg and femur length was associated with lower BMD and BMC and greater bone geometrical measures, with opposing associations for knee height and crural index. Associations with osteoarthritis severity showed similar patterns. These associations were partly or wholly attenuated by adjustment for BMI. Age interactions showed decreased strength of BMC and BMC associations in older age, for example crural index and hip BMD where standardised estimates were 0.14 (95%CI 0.1-0.19) for 60-69y, 0.09 (0.03-0.16) for 70-79y and 0.04 (-0.05 to 0.13) for those aged 80 and over. In contrast, associations with bone geometry and KL score were generally consistent across age.

This is the first study to examine associations between limb proportions and bone health outcomes, and our findings also broadly agree with previous observations in osteoarthritis. Although associations were not attenuated by adult socioeconomic indicators, we could not explore childhood markers. That lower BMD and reduced osteoarthritis risk were observed in individuals with longer femurs or shorter tibiae may implicate loading as a mediating factor. Hence, decreased strength of BMD and BMC associations in older age may be explained by reduced mechanosensitivity. These results highlight limb proportions as a potential risk factor for osteoporosis as well as osteoarthritis.

### P36

### Lifestyle and Clinical Factors Associated with Femoral Neck Bone Mineral Density in African Adults: Findings from the Fracture-E3 Study

Camille Pearse[1], Lucy Gates[1], Anya Burton[2], Momodou Jallow[3], Ted Manyanga[4], Chris Grundy[5], Nic Crabtree[6], Emma Clark[7], Hannah Wilson[8], Anthony Manyara[8], Tafadzwa Madanhire[8], Bilkish Cassim[9], Rashida Ferrand[4], Celia Gregson[8], Kate Ward[1]

[1]University of Southampton, Southampton, UK. [2]University of Bristol, Bristol, UK. [3]MRC Unit The Gambia, Banju, Gambia. [4]The Health Research Unit Zimbabwe, Harare, Zimbabwe. [5]MRC International Statistics and Epidemiology Group, London, UK. [6]Birmingham Women's and Children's NHS Trust, Birmingham, UK. [7]Musculoskeletal Research Unit, Bristol, UK. [8]Bristol Medical School, Bristol, UK. [9]University of KwaZulu-Nata, KwaZulu-Natal, South Africa

### Abstract

**Background:** Low bone mineral density (BMD) and related fragility fractures are increasingly recognised as important contributors to morbidity in ageing populations globally, yet data from African populations remain limited. Nutritional stress, chronic disease burden, and lifestyle transitions may influence skeletal health across the lifecourse, but the relative contribution of these factors is poorly understood in sub-Saharan Africa. Improved understanding of these determinants is urgently needed to inform context-appropriate prevention strategies and ensure early identification of individuals at greatest risk of fracture.

**Aims:** To identify clinical, nutritional and lifestyle determinants of femoral neck BMD (FN-BMD) in women and men from Africa.

**Methods:** A population-based sample of adults aged ≥40 years, stratified by sex- and age-band, were recruited in The Gambia and Zimbabwe. FN-BMD was measured using DXA, and vertebral fractures (VF) were identified using the Genant semi-quantitative method. Linear regression examined associations between demographic, nutritional, lifestyle and clinical factors and femoral neck T-scores, stratified by sex and adjusted for age, results after adjustment for age shown on figure. Factors assessed included underweight status (BMI <18.5kg/m$^2$), mid-upper arm circumference (MUAC), HIV (Zimbabwe only), diabetes, glucocorticoid use, fracture history, rheumatoid arthritis, secondary osteoporosis, smoking and alcohol intake.

**Results:** In both men and women, markers of undernutrition were the strongest predictors of lower BMD. Underweight status and MUAC <23 cm showed robust associations after age adjustment. Previous major osteoporotic fracture (MOF) and VF were consistently associated with reduced BMD. HIV infection was independently associated with lower BMD in men, while type 2 diabetes was associated with lower BMD in women. Associations for smoking, alcohol intake and secondary osteoporosis were attenuated after controlling for age.

**Conclusion:** Undernutrition and previous fractures (MOF & VF) were the strongest predictors of lower FN-BMD. HIV infection in men and diabetes in women showed sex-specific associations, highlighting important clinical differences. These findings highlight the value of combining nutritional indicators and fracture history with BMD to identify adults at increased fracture risk in African populations.

Figure. Sex-specific associations of risk factors with FN-BMD

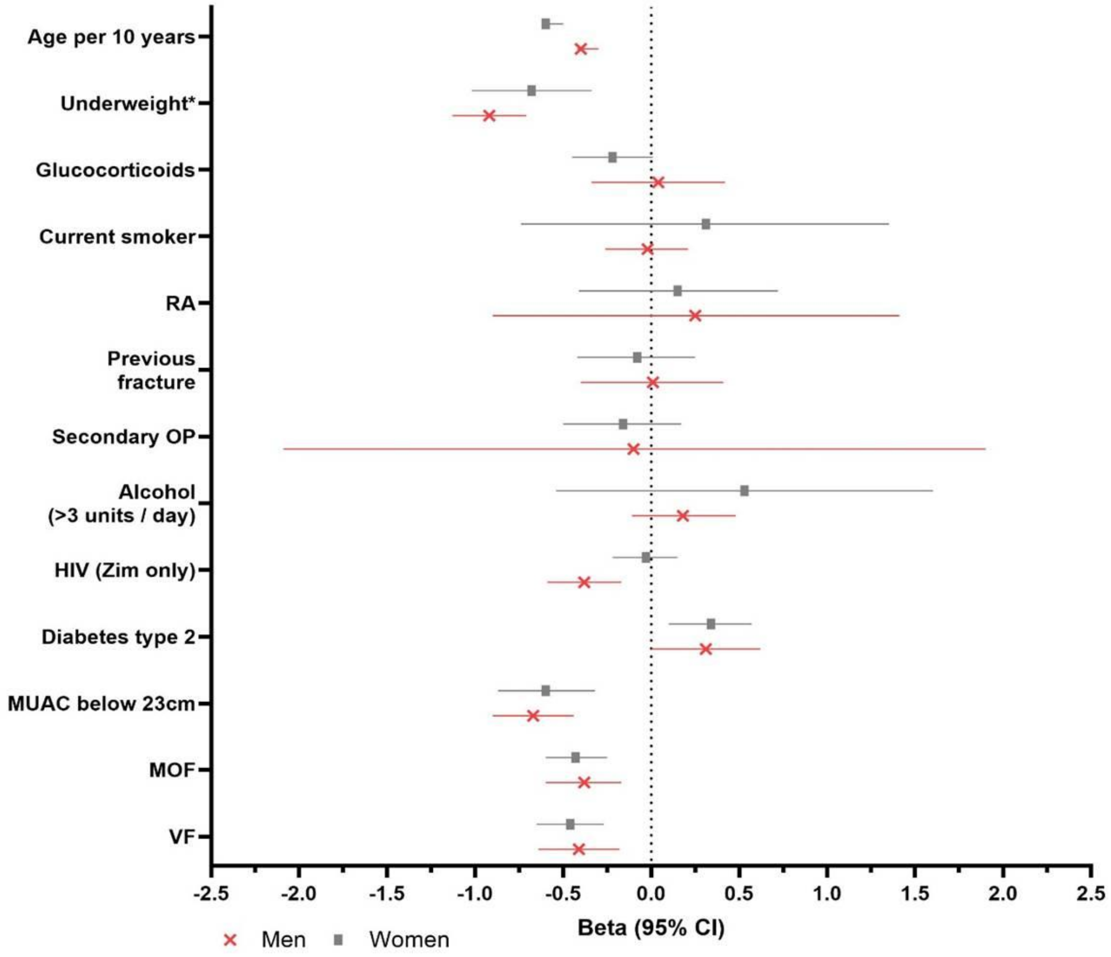

Beta and 95% CI after adjustment for age; *underweight compared to others; MUAC below 23cm compared to others

### P37

### Prospective Muscle-Fat-Fracture Relationships in UK Biobank are Sex and Distribution-Specific

Elizabeth Curtis[1,2], Sharon Lin[1], Stefania D'Angelo[1], Leo Westbury[1,2], Rebecca Moon[1,2,3], Cyrus Cooper[1,2], Kate Ward[1,2], Nicholas Harvey[1,2]

[1]MRC Lifecourse Epidemiology Centre, Southampton, UK. [2]NIHR Southampton Biomedical Research Centre, University of Southampton, Southampton, UK. [3]Department of Paediatric Endocrinology, University Hospital Southampton NHS Foundation Trust, Southampton, UK

### Abstract

### Objectives

Our aim was to quantify the prospective associations between site-specific MRI-derived fat and muscle volumes and incident fracture in the UK Biobank Imaging Study.

### Materials and Methods

UK Biobank Imaging Study participants with MRI-derived visceral adipose tissue volume (VAT), abdominal subcutaneous adipose tissue volume (ASAT) and thigh muscle volume (TMV) as exposures. The primary outcome was first osteoporotic fracture, ascertained via hospital record linkage. Cox proportional hazards models were used to estimate hazard ratios (HRs) per standard deviation (SD) increase in each exposure, adjusting for age, socioeconomic deprivation, smoking, exercise, diabetes, and prior fracture. Femoral neck bone mineral density (BMD) adjustments were applied in sensitivity analyses. Participants were followed until first osteoporotic fracture, death or loss to follow-up.

### Results

The dataset comprised 63,253 participants (mean age 64.3 years in women, 65.7 years in men) with MRI data. 2130 incident osteoporotic fractures occurred during a total 240,405 person-years follow-up time. In fully adjusted models, higher ASAT was consistently associated with lower fracture risk in both sexes [women HR/SD (95% CI) 0.78(0.73–0.83; men 0.86(0.79–0.95)], with similar findings for VAT [women 0.78(0.73-0.84); men 0.89 (0.82-0.97)]. These associations remained robust after adjustment for BMD, but not for VAT in men. Greater TMV was protective in women [0.81(0.73-0.90)] but this relationship was removed by adjustment for BMD [0.96(0.86-1.08)], and was not observed in men [1.11(0.96-1.28)].

### Conclusions

Higher subcutaneous fat volume appears protective against osteoporotic fracture in both sexes, while visceral fat shows a similar (but BMD dependent) protective pattern in women but not men. Lower thigh muscle volume was associated with fracture risk in women but not men. These findings may inform fracture risk assessment and biological understanding of body composition in skeletal health.

(UK Biobank 3593).

### P38

### Continued Improvements in Lower Extremity Alignment in Navepegritide-Treated Children with Achondroplasia: Week 104 Results from the ApproaCH Trial

Melita Irving[1], Leanne Ward[2,3], Daniel Hoernschemeyer[4], Janet Legare[5], Hanne Hove[6], Carlos Bacino[7], Philippe M Campeau[8], Paul Hofman[9], Josep Maria de Bergua Domingo[10], M Jennifer Abuzzahab[11], Stefan Jackowski[3], Kevin Smit[3], Andrew Tice[3], Sasha Carsen[3], Helle Væver Harvig[12], Lærke Clement Freiberg[12], Michael Makara[13], Michael Ominsky[13], Aimee Shu[13], Ravi Savarirayan[14,15,16], Ciara McDonnell[17,18]

[1]Guy's and St. Thomas' NHS Foundation Trust, Evelina Children's Hospital, London, UK. [2]University of Ottawa, Ottawa, Canada. [3]Children's Hospital of Eastern Ontario, Ottawa, Canada. [4]University of Missouri Children's Hospital, Columbia, USA. [5]University of Wisconsin School of Medicine and Public Health, Madison, USA. [6]Copenhagen University Hospital, Rigshospitalet, Copenhagen, Denmark. [7]Baylor College of Medicine, Houston, USA. [8]CHU Sainte-Justine Research Center, Montreal, Canada. [9]The Liggins Institute, University of Auckland, Auckland, New Zealand. [10]Unidad de Cirugia Artroscopica, Vithas Vitoria Hospital, Vitoria-Gasteiz, Spain. [11]Children's Minnesota, Minneapolis, USA. [12]Ascendis Pharma A/S, Hellerup, Denmark. [13]Ascendis Pharma Inc., Palo Alto, USA. [14]Murdoch Children's Research Institute, Parkville, Australia. [15]Royal Children's Hospital, Parkville, Australia. [16]University of Melbourne, Parkville, Australia. [17]Children's Health Ireland at Temple Street, Dublin, Ireland. [18]University of Dublin, Trinity College, Dublin, Ireland

### Abstract

**Objectives:** In children with achondroplasia, disproportionate and reduced growth contribute to lower extremity malalignment, which is often associated with pain, altered mobility, and need for surgical intervention. Navepegritide, an investigational prodrug of C-type natriuretic peptide (CNP) administered once-weekly by subcutaneous injection, is designed to provide sustained release and continuous exposure of active CNP to counteract constitutively active fibroblast growth factor receptor 3 (FGFR3) signaling in achondroplasia. In the pivotal randomized, double-blind, placebo-controlled ApproaCH Trial, children treated with navepegritide for 52 weeks had superior annualized growth velocity (AGV) versus placebo (primary endpoint), and improved proportional growth and radiographic alignment in the lower extremities. Here we report radiographic outcomes at 104 weeks.

**Methods:** ApproaCH enrolled 84 children (aged 2-11 years) with achondroplasia. Participants were randomized 2:1 to receive navepegritide 100 mg/kg/week (n=57) or placebo (n=27) for 52 weeks, followed by 52 weeks of navepegritide treatment for all participants during the open-label extension (OLE). As an exploratory endpoint, lower extremity radiographs were assessed annually by blinded central readers using a pre-specified measurement protocol.

**Results:** Continued improvements in indices of lower extremity alignment were observed during the OLE, with decreased mean tibial-femoral angle (TFA) in children continuing navepegritide treatment, as well as those who switched from placebo to navepegritide at Week 52 (Table). The TFA improvement in the placebo-navepegritide group in the OLE was in line with navepegritide-related improvements observed at Week 52. Even greater correction was evident in children with preexisting genu varum (TFA ≥5°). In the subgroup of children with preexisting genu varum at trial baseline, mean TFA was reduced from 13.3° at baseline to 8.0° after 104 weeks of navepegritide treatment. During the OLE, fibula-to-tibia length ratio remained stable in the overall population, reflecting proportional growth of the lower leg.

**Conclusions:** In the ApproaCH Trial, weekly administration of navepegritide continued to improve lower limb alignment in children with achondroplasia through Week 104, with greater effects in those with more pronounced genu varum. These findings suggest that through continuous systemic exposure to active CNP, navepegritide may address features of skeletal dysplasia that contribute to major health-related complications associated with achondroplasia.

**Table.**

| | Treatment group (DB period/OLE) | |
|---|---|---|
| | **navepegritide/navepegritide** | **placebo/navepegritide** |
| **Mean [SE] change in TFA (degrees), Week 0 to Week 52 (DB Period)** | | |
| All children | -1.33 [0.41] (n=54) | +0.21 [0.52] (n=26) |
| Children with TFA ≥5° at Week 0 (Baseline) | -2.07 [0.75] (n=26) | +0.51 [0.96] (n=13) |
| **Mean [SE] change in TFA (degrees), Week 52 to Week 104 (OLE)** | | |
| All children | -0.80 [0.34] (n=53) | -1.70 [0.67] (n=26) |
| Children with TFA ≥5° at Week 52 | -1.84 [0.66] (n=20) | -3.54 [0.70] (n=16) |

DB, double-blind; OLE, open label extension; SE, standard error; TFA, tibial-femoral angle
Data reported are observed mean [SE] absolute change in TFA among children with data available at both time points; negative numbers indicate improvement in TFA toward neutral alignment.

### P39

### Imaging Altered Fibrillar-Level ECM Mechanics across the Bone-Cartilage Interface in Ageing and Injury

Himadri Shikhar Gupta[1], Waqas Badar[1], Nicholas Terrill[2], Tim Snow[2], Martin M Knight[1]

[1]Queen Mary University of London, London, UK. [2]Diamond Light Source, Harwell, UK

### Abstract

The mechanics of the bone–cartilage unit (BCU) are essential for pain-free articulation in diarthrodial joints and depend on the structure and mechanics of the extracellular matrix (ECM)[1-2]. The ECM is hierarchical, consisting at the nanoscale of type-II collagen fibrils embedded in a hydrated proteoglycan matrix in cartilage, transitioning through a calcified interface into subchondral bone. Age-related compositional changes such as advanced glycation end-product (AGE) cross-linking and proteoglycan loss may reduce resistance to injurious loads and contribute to osteoarthritis onset. Using a bespoke high-brilliance imaging technique based on microbeam synchrotron small-angle X-ray scattering (SAXS)[3], in-vitro ribose-induced cross-linking (to simulate AGE accumulation), and controlled impact loading, we investigate how ageing-related ultrastructural changes alter nanoscale mechanical homeostasis and how these changes interact with injurious loading to affect fibrillar deformation across the BCU interface.

BCU cores were extracted from bovine metacarpal–phalangeal joints (16–24 months). Samples were cultured for 7 days in DMEM supplemented with 0.6 M D-ribose to induce cross-linking and divided into control, low-injury (0.02 J) and high-injury (0.12 J) groups. Injuries were applied using a drop-tower device, followed by 7 days incubation to allow cellular responses. Synchrotron SAXS scans were performed at Diamond Light Source (beamline I22; ~20 µm beam). Samples were mapped across cartilage, the calcified plate and trabecular bone in a PBS-filled chamber within a micro-compression rig, then re-scanned after 20 % compressive strain. Ultrastructural parameters including fibrillar D-period (reflecting pre-strain), D-period variability, fibril orientation and alignment were extracted for zone-specific analysis of ageing, injury level and applied load.

SAXS mapping revealed clear age-related ultrastructural differences. In aged tissue, the collagen D-period decreased in the calcified plate(CP), indicating reduced fibrillar pre-strain, while fibril radius increased (~10–15 %) in the deep zone(DZ) and CP. Injury also altered the sharp transition in D-period across the BCU interface, making it more diffuse. The injury response was age-dependent: untreated tissue showed progressive loss of fibrillar pre-strain with increasing injury (~1 %, ~10 MPa), whereas this response was absent in aged tissue, which already exhibited reduced baseline pre-strain.

These results quantify how in young tissue the ultrastructural collagen fibrillar network can dynamically expand and contract to stabilise the proteoglycan gel phase, whereas ageing disrupts this resilience. Altered elasticity in the calcified plate may promote stress localisation, highlighting nanoscale mechanisms linking ageing, injury response and osteoarthritis initiation.

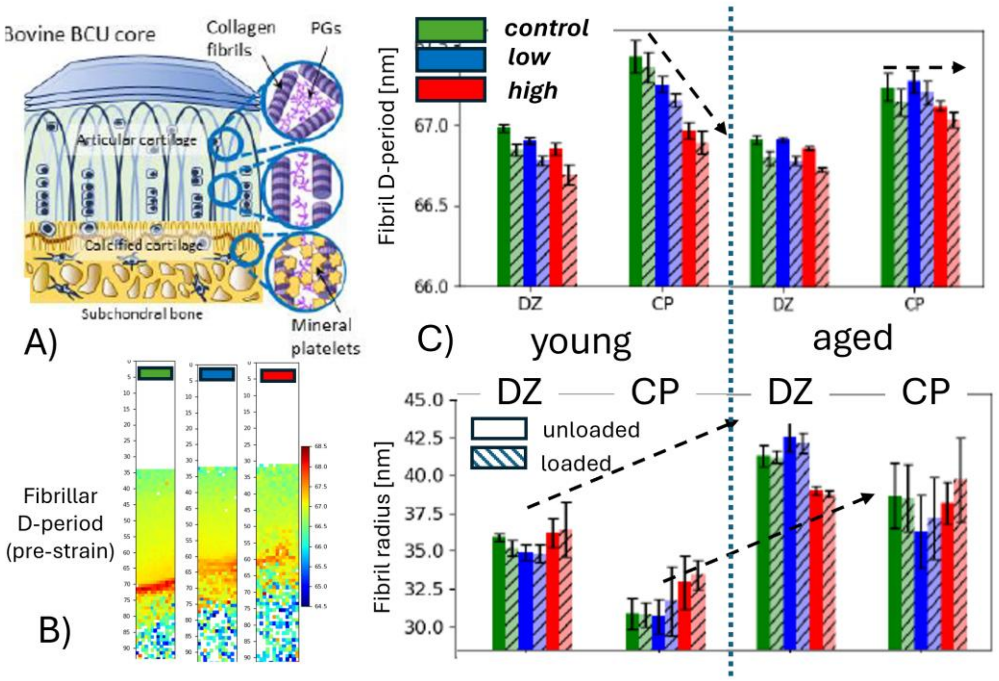

**Figure 1:** (A) Schematic of BCU core. (B–C) 2D colour maps and bar plots of D-period and fibril radius at different injury levels(control, low, high).

**References**: [1] https://doi.org/10.1038/nrrheum.2016.148 [2] https://doi.org/10.1002/jor.23204 [3] https://doi.org/10.1002/advs.202407649

### P40

### Crystallite Nanostructure Differs in Perilacunar Matrix Compared to Distant Matrix in Iliac Crest Cortical Bone from Patients with Type 2 Diabetes

Maja Østergaard[1], Karoline Lauridsen[2], Sebastian Zanner[2,3], Torgom Yezekyan[4,5], Jonas Beermann[5], Charlotte Ejersted[3], Sergey I Bozhevolnyi[5], Henrik Birkedal[1], Morten Frost[2,3], Eva M Wölfel[2,3]

[1]Department of Chemistry and iNano, Aarhus University, Aarhus, Denmark. [2]Molecular Endocrinology Department, Clinical Institute, University of Southern Denmark, Odense, Denmark. [3]Department of Endocrinology, Odense University Hospital, Odense, Denmark. [4]Center for Polariton-driven Ligth-Matter Interactions, Mads Clausen Institute, University of Southern Denmark, Odense, Denmark. [5]Nano Optics, Mads Clausen Institute, University of Southern Denmark, Odense, Denmark

### Abstract

**Introduction:** Metabolic diseases such as type 2 diabetes can lower bone strength leading to higher fracture risk. Bone strength is determined by the interplay of different structural, chemical, and cellular composites within the bone matrix. Osteocytes, mechano-sensing cells residing inside the bone matrix, orchestrate bone remodeling, a process to maintain bone mass and quality. The perilacunar matrix of osteocytes plays an important role in strain amplification and mechanosensation, which can influence osteocyte specific regulation of bone turnover. Changes in the perilacunar region can implicate altered mechanosensation, osteocyte signaling, and ultimately bone turnover. Osteocyte lacunae in cortical bone in iliac crest biopsies from patients with type 2 diabetes and microvascular disease (T2D+MVD) were larger compared to type 2 diabetes (T2D) alone. Here, we investigated the nano-scale mineral properties of the perilacunar matrix in this cohort with larger osteocyte lacunae in T2D+MVD.

**Methods:** At the microfocus beamline ID13 at the European Synchrotron Research Facility (Grenoble), 10 bone samples (5 T2D+MVD and 5 T2D) were scanned using transmission X-ray diffraction with the X-ray beam focused to 340 x 340 $nm^2$ and 250 nm step size. For each sample, 20 osteocyte lacunae in cortical bone were imaged with 40 x 40 $\mu m^2$ maps resulting in 25 600 data points per lacuna. Apparent crystallite length, crystallite width, and unit cell *c*-axis length in hydroxyapatite crystals surrounding the osteocyte lacuna were determined. Additionally, bone biopsies were scanned with 6 µm resolution using microCT to assess general microstructure and sections were processed with silver nitrate precipitation to characterize the lacuno-canalicular network using machine learning for quantification.

**Results:** When comparing T2D+MVD with T2D no significant differences in microstructure, nanostructure of crystallites, and lacuno-canalicular network were found. However, we observed an increasing apparent crystallite length and unit cell *c*-axis length with increasing distance from the osteocyte lacuna. Specifically, we observed a smaller apparent crystallite length (peri: 30.91± 2.043 nm, distant: 32.47± 2.49 nm, median ± IQR, $p<0.001$) and unit cell *c*-axis length (peri: 6.89265 ± 0.0018 Å, distant: 6.89314 ± 0.00198 Å, median ±IQR, $p=0.002$) in the perilacunar (0-5 µm distant) compared to the more distant (15-20 µm distant) matrix in all biopsies combined.

**Conclusion:** While we studied samples from patients with T2D, the data suggests that the observed smaller hydroxyapatite crystallites in the perilacunar matrix compared to more distant matrix are a general feature of the complex and heterogeneous bone matrix properties around the osteocyte lacuna, rather than an MVD-specific feature.

### P41

### 4-Octyl Itaconate Protects Osteoblasts from Ferroptosis and Attenuates Osteoid Accumulation in Iron Overload

Xuan-Thanh Le-Phuoc, Vanessa Passin, Maria Ledesma-Colunga, Heike Weidner, Ulrike Baschant, Lorenz Hofbauer, Martina Rauner

Medical Faculty and University Hospital Carl Gustav Carus, Dresden University of Technology, Department of Medicine III & Center for Healthy Aging, Dresden, Germany

### Abstract

Intravenous iron formulations are widely used as first-line therapy for iron-deficiency anemia due to their rapid corrective effect; however, repeated infusions have been associated with osteomalacia. 4-Octyl itaconate (4OI), a derivative of the immunometabolite itaconate, has demonstrated anti-oxidative properties against ferroptosis. Here, we report that 4OI restores osteoblast differentiation and mineralization by preserving lipid droplet integrity and attenuating iron-induced lipid peroxidation and ferroptosis.

Male 12-week-old C57BL/6J mice received PBS, MonoFer (0.5 g/kg body weight), or MonoFer plus 4OI (50 mg/kg body weight) twice weekly for 4 weeks. We assessed bone microarchitecture and mineralization by µCT and histomorphometry. Iron overload significantly reduced bone volume (2-fold, $p<0.001$) and increased osteoid volume (+34%, $p<0.001$). Although co-administration of 4OI did not rescue bone volume loss, it selectively reversed osteoid accumulation, suggesting a protection of osteoblast function. To investigate the underlying mechanism, we differentiated primary mouse osteoblasts from BMSCs for 7 days, then challenged them with ferric ammonium citrate (FAC, 50 µM) with or without 4OI (50 µM) for 48 hours. FAC triggered an early ROS surge (DCFDA; 2-fold at 2 hours, $p<0.05$), followed by elevated lipid peroxidation (BODIPY C11; 1.7-fold, $p<0.0001$) and cell death (Sytox; 2-fold, $p<0.002$). 4OI rescued osteoblasts from FAC-induced ferroptosis, reducing oxidative burden ($p<0.05$) and preserving lipid droplet integrity (LipidTox). Inhibiting lipid droplet biogenesis with DGAT1/2 inhibitors (T863, 20 µM; PF-06424439, 10 µM) abolished 4OI-mediated protection, identifying lipid droplet sequestration as a key anti-ferroptotic mechanism through which 4OI preserves osteoblast viability under iron overload.

Taken together, iron overload induced ferroptosis in osteoblasts and impaired mineralization in vivo. 4OI, acting as an antioxidant, reversed the lipid peroxidized state of iron-overloaded osteoblasts, suggesting its potential as a therapeutic strategy against ferroptosis-related bone loss.

### P42

### Acute High-Impact Drop-Landing Modulates Bone Remodelling Regulators and Circulating Extracellular Vesicles in Young Men

Zhuoyue Zhang, Owen Davies, Liam Heaney, Katherine Brooke-Wavell

Loughborough University, Loughborough, UK

### Abstract

**Background:** High-impact exercise, as a potent form of mechanical loading, regulates mechanosensitive pathways involved in bone remodelling, including Wnt signalling and RANK/RANKL/OPG axis. Extracellular vesicles (EVs) are emerging mediators of intercellular communication in bone and may serve as mechanotransductive interface between local skeletal strain and systemic responses. However, whether acute high-impact mechanical loading elicits coordinated changes in EVs alongside bone remodelling regulators remains unclear. Drop-landing exercise provides high-impact stimulus with minimal sustained neuromuscular and cardiovascular demand, enabling investigation of acute responses to mechanical loading. The study aims to investigate whether drop-landing modulates bone remodelling regulators and circulating EV in healthy young men.

**Methods:** In this randomised controlled crossover study, 15 participants completed drop-landing and control trials on separate days (>7-day interval). Drop-landing involved participants performing 60 drop-landings from a block (~4×body weight peak ground reaction force), with standardised rest intervals, followed by 1-hour seated recovery. In control trial, participants performed low-impact, time- and repetition-matched stepping. Blood samples were collected pre-, immediately post, and 1-hour post drop-landing/control. Serum sclerostin, DKK1, and OPG concentrations were quantified using ELISA. EVs were isolated by differential ultracentrifugation. Circulating particle (25-1000nm) numbers were determined by nanoparticle tracking analysis. Changes from baseline were calculated for each time point. Repeated measures ANOVA compared the within-subject effects of time (pre-, immediately post, and 1-hour post), trial (drop-landing vs. control), and time×trial interaction.

**Results:** Fifteen male participants (age 24.8±3.5years) completed the study. Sclerostin change showed significant time×trial interaction (P=0.008), increasing immediately following drop-landing versus control (P=0.044). Within control trial, sclerostin increased from immediately post to 1-hour post (P=0.024). DKK1 remained unchanged in both trials. OPG demonstrated significant time (P=0.035) and interaction effects (P<0.001). OPG was reduced at 1-hour post-drop-landing versus control (P=0.008). In control, OPG increased at 1-hour relative to both baseline (P=0.004) and immediately post (P=0.007). Circulating particle concentration showed significant trial (P=0.025) and interaction effects (P=0.038), increasing immediately post-drop-landing versus control (P=0.003). In control trial, circulating particle numbers declined, with values at 1-hour lower than baseline (P=0.020).

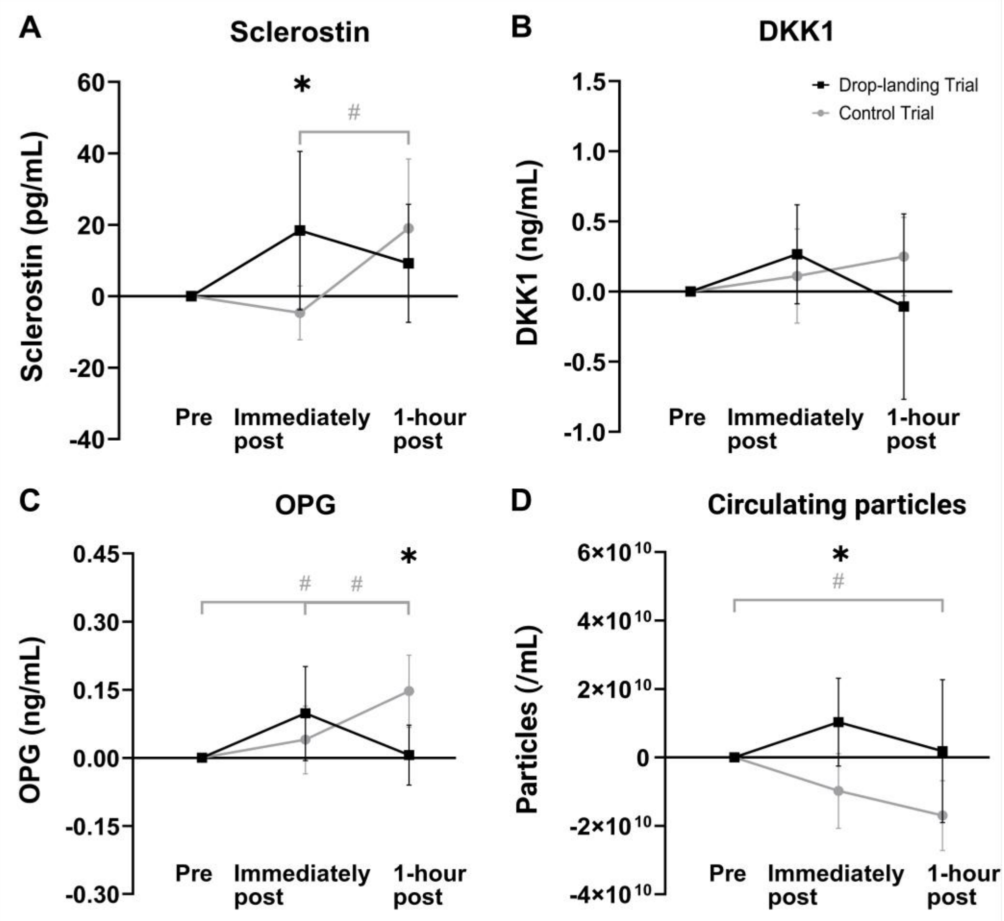

Figure 1. Changes from baseline in sclerostin (A), DKK1 (B), OPG (C), and circulating particle numbers (D) during drop-landing and the control trials. Data are presented as mean with 95%CI. *Significantly different between trials at the same time point ($P < 0.05$). #Significantly different within a trial across time ($P < 0.05$).

**Conclusion:** Acute drop-landing induced transient increases in sclerostin and circulating particle (including EVs) numbers versus control, followed by a reduction in OPG at 1-hour recovery versus control. While EVs are likely to be present within this fraction, further work is required to determine its composition. These findings suggest coordinated systemic responses to mechanical loading and a possible role for EVs in early mechanoadaptive signalling.

### P43

### Coding of Fragility Fractures in Primary Care and its Impact on Osteoporosis Risk Assessment: A Retrospective Study

Noor Khan, Aana Kapoor

Newcastle University, Newcastle, UK

### Abstract

**Background:**

Fragility fractures are often the first presentation of osteoporosis and are associated with significant morbidity and mortality in the UK. NICE recommends systematic osteoporosis risk assessment following fragility fractures, including FRAX scoring and DEXA scanning where appropriate. This study aimed to assess whether fragility fractures are appropriately coded in primary care records and to evaluate the association between coding and subsequent documentation of osteoporosis risk assessment.

**Methods:**

A retrospective review of electronic records was conducted in one GP practice. Patients aged over 50 with a fracture recorded between April 2024 and April 2025 were identified. Non-fragility fractures and predefined exclusions were removed. For each patient, data was collected on whether the fracture was coded as a fragility fracture and whether FRAX scores and DEXA results were documented.

**Outcomes:**

Twenty-nine patients met inclusion criteria. Of these, 69% did not have their fracture coded as fragility. Among uncoded cases, 12 had no documented FRAX score and 7 of these also lacked a recorded DEXA scan. Overall, 34% had no documented DEXA measurement. Appropriate coding was associated with improved documentation of osteoporosis assessment.

**Discussion:**

Accurate coding in primary care can influence clinical follow-up, risk stratification, and secondary fracture prevention. Suboptimal coding could represent a missed opportunity for early osteoporosis identification and intervention. Our findings suggest under-coding may contribute to gaps in longitudinal bone health evaluation and fragility fracture prevention, highlighting the need for improved education and communication on bone health status between primary and secondary care.

**P44**

Abstract Withdrawn

### P45

### Traditional Chinese Medicine (TCM) Formula Er Zhi Wan (EZW) Enhances Skeletal Muscle and Bone Properties in Ageing Osteosarcopenia Animal Model

Chun Au-Yeung[1], Christina Chui-Wa Poon[2], Man-Sau Wong[1,2]

[1]Department of Food Science and Nutrition, The Hong Kong Polytechnic University, Kowloon, Hong Kong. [2]Research Centre for Chinese Medicine Innovation, The Hong Kong Polytechnic University, Kowloon, Hong Kong

### Abstract

**Background:**

Osteosarcopenia, a coexistence of osteoporosis and sarcopenia, is a major contributor to the global burden of disease and disability because of its high prevalence among ageing populations and its association with increased risks of falls and fragility fractures. Er Zhi Wan (EZW) is a traditional Chinese medicine (TCM) formula widely used to strengthen tendons and bones. It consists of *Fructus Ligustri Lucidi* (FLL) and *Ecliptae Herba* (EH) in a 1:1 (w/w) ratio, which may exert multi-organ biological effects to support the musculoskeletal system by nourishing and tonifying "kidney yin". Thus, the present study aimed to determine the *in vivo* effects of EZW in musculoskeletal protection against age-related skeletal muscle and bone loss in osteosarcopenic animal model.

**Methods:**

Senescence-Accelerated Mouse Prone 8 (SAMP8) mice at 8-month-old (n = 6-8 per group) were randomly assigned to receive EZW treatment, high calcium diet (HCD; 1% calcium in diet), high vitamin D diet (HVD; 7.8 IU/g), or vehicle (saline) for 8 weeks, and compared with age-matched Senescence-Accelerated Mouse Resistant 1 (SAMR1) control mice (n = 8). The effect on age-related muscle loss was evaluated by assessing muscle function with a digital grip strength meter (Harvard Apparatus, USA). Additional evaluation was performed on tibialis anterior (TA) muscles by measuring myofibre cross-sectional area (fCSA), the composition of myosin heavy chain (MHC) isoforms (type I, IIa, and IIb), and myogenic differentiation capacity of primary satellite cells (MuSCs). Effects on bone loss were assessed by determining trabecular bone mineral density (BMD) at distal femur and proximal tibia by using micro-computed tomography (Micro-CT; Bruker Skyscan 1276).

**Results and conclusion:**

Our results demonstrated that EZW could exert stimulatory effects on skeletal muscle properties, as evidenced by increased grip strength ($p < 0.01$), fCSA ($p < 0.05$), the proportion of glycolytic type IIb myofibres ($p < 0.05$), and expression of myogenic markers ($p < 0.05$) in TA muscles. It also could improve BMD at both distal femur and proximal tibia ($p < 0.05$). This study provides scientific evidence supporting the use of EZW in managing age-related osteosarcopenia.

***Acknowledgement:***

*This work was supported by the Health and Medical Research Fund of Hong Kong Health Bureau (19200411) and PolyU internal funds (1-ZVXG and 1-BD4V). We also thank the University Research Facility in Life Sciences at The Hong Kong Polytechnic University for the technical support.*

### P46

### Impact of Romosozumab Followed by Anti-resorptive Therapy on Bone Mineral Density in Patients with Osteoporosis at South Tyneside and Sunderland Hospitals

Zahra M Iftikhar[1], Sajeel Ahmed[1], Vasiliki Syrmou[2,3], Burhan Choudhry[4], Amogha Ramasharan[4], Shahzad Azam[1], Nikhil Abraham[1], Kanchan Manchegowda[1]

[1]South Tyneside and Sunderland NHS foundation trust, Sunderland, UK. [2]University General Hospital of Larissa, Larissa, Greece. [3]University of Thessaly, Larissa, Greece. [4]Newcastle University, Newcastle, UK

### Abstract

**Background:**
The introduction of Romosozumab has transformed the management of severe osteoporosis, offering rapid and substantial anabolic gains in bone mineral density (BMD). However, its effectiveness in real-world practice depends not only on pharmacology, but on timely referral pathways, equitable service access, prescribing governance, and appropriate sequential therapy. Following the expansion of romosozumab prescribing across Rheumatology, Metabolic Services, and Care of the Elderly (COTE) at South Tyneside and Sunderland hospitals, we undertook a service evaluation to assess pathway efficiency, prescribing appropriateness, treatment completion, and post-treatment BMD outcomes.

**Methods:**
This retrospective audit included 93 patients referred for romosozumab between 2022-2025. Demographics, indications, referral source, waiting times, treatment completion, adverse events, post-treatment sequencing, and dual-energy X-ray absorptiometry (DEXA) outcomes were analysed. Between-service comparisons used Kruskal–Walli's testing with Bonferroni-adjusted post-hoc analysis. Paired t-tests and Wilcoxon signed-rank tests assessed BMD changes.

**Results:**
Mean age was 69.1 years (range 53–89). Romosozumab was clinically indicated in 83.9%, predominantly for severe osteoporosis (52.7%) and fragility fractures (31.2%). The Fracture Liaison Service generated 52.5% of referrals, followed by primary care (29.0%). Patients were mainly reviewed by Rheumatology (49.5%) or Metabolic Services (43.0%).

Waiting times differed significantly between services ($p<0.001$). Rheumatology had the shortest median referral-to-appointment interval (54 days) compared with Metabolic Services (159 days) and COTE (253.5 days), with moderate-to-large effect sizes. This difference may partly reflect staffing factors, as more Rheumatology consultants are involved in osteoporosis management compared with other services. Time from review to treatment initiation did not differ significantly (median 28–35 days), suggesting comparable prescribing efficiency once assessed.

After excluding patients still on treatment (n=20) or who declined therapy (n=6), 67 patients were analysed for completion. Sixty (89.6%) completed 12 months of romosozumab. Adverse events occurred in 6.5%; only two discontinuations were attributable to side effects.

Of completers, 71.6% commenced sequential therapy, predominantly bisphosphonates: alendronate (44.8%) and zoledronic acid (41.4%). Treatment continuity was high, with 95% transitioning without a break exceeding one month.

Repeat DEXA was available in 18 patients (30%). Lumbar spine mean T-score improved from −3.09 to −2.26 (mean +0.83), demonstrating significant improvement (paired t-test $p<0.001$; Wilcoxon $p=0.00029$). Among 12 patients with paired hip data, mean T-score improved modestly (+0.26) but did not reach statistical significance ($p=0.052$).

**Conclusions:**
Romosozumab was appropriately prescribed in most cases, with high treatment completion and low adverse event rates. Significant inter-service variation in waiting times was observed, favouring Rheumatology. Lumbar spine BMD improved significantly, confirming robust anabolic efficacy in real-world practice. However, incomplete post-treatment DEXA monitoring and suboptimal sequencing rates highlight areas for service improvement.

### P47

### An Alzheimer's-Susceptible Signalling Pathway Operating in Bone and its Potential Involvement in Maintaining Bone Mass?

Simon McArthur, David Mills, Stefaan Verbruggen, Simon Rawlinson

Queen Mary University of London, London, UK

### Abstract

Individuals with Alzheimer's disease are more likely to suffer from osteoporosis than age-matched individuals; significantly contributing to the frailty associated with dementia. Notably, many of the key pathogenic markers of Alzheimer's disease, including APP, BACE1 and β-amyloid peptide, have been found within osteoporotic bone, but their function there remains unclear.

Age-related bone loss and disuse osteoporosis are restricted to predominantly the load-bearing elements of the skeleton, but not the skull or basal bone of the mandible. This disparity of response suggests differing regulatory mechanisms at these sites. Curiously, there are molecular parallels between the pathways controlling bone strength and remodelling and those known to regulate neuronal networks, with numerous 'neuronal' components, including neurotransmitters, receptors and signalling molecules, having been identified in bone cells. In an attempt to elucidate a functional role, we have investigated responses to exogenous glutamate [1] in cell lines derived from the skull and limb bones and, additionally, examined the distribution of the glutamatergic NMDA receptor in resident osteocytes in parietal and ulna bones of wild-type and 5xFAD Alzheimer's disease model mice.

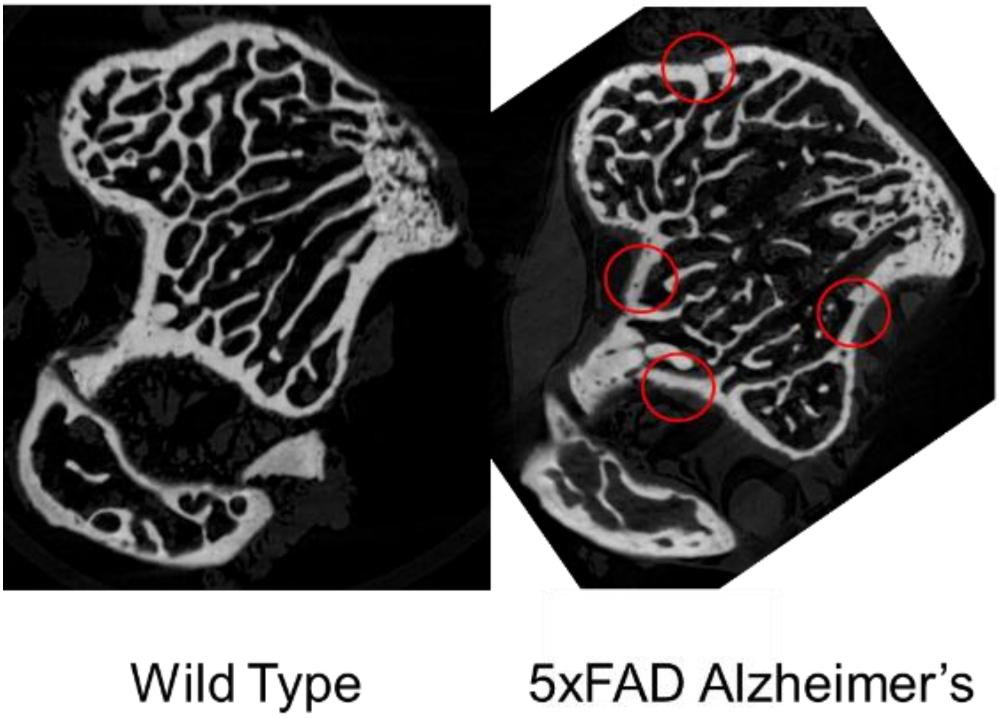

Calvarial bone cells, MC3T3-E11, and limb bone MG63 cells were exposed to glutamate agonist for 30 minutes and glcose-6-phosphate dehydrogenase (G6PD) activity levels recorded. G6PD activity increased in MG63, but not MC3T3 cells. This finding is consistent with immunostaining for the NMDA receptor subunit NR1 in bones – present in limb, but not skull bone. Notably, prior incubation of cells with the Alzheimer's disease-associated peptide ß-amyloid negated the G6PD response in MG63 cells.

Long bones from the 5xFAD, an Alzheimer's mouse model, were examined by XMT at 5 months of age, a point where pathology is evident in the brain, but cognitive function has not yet been impaired. 5xFAD mice had thinner cortices and fewer trabeculae in the proximal tibia compared with age-matched wild type mice.

The pattern of age-related and disuse osteoporosis matches the sites of bone loss in Alzheimer's patients. Whether bone loss in Alzheimer's disease can be attributed to the effects of activity levels in these patients is debatable as not everyone who suffers from Alzheimer's disease has bone loss. It is nonetheless interesting to speculate on the presence of an Alzheimer's-susceptible signalling pathway operating in bone that is involved in maintaining bone mass. Loss of such a mechanostat function would lead to inappropriate bone loss, potentially contributing to Alzheimer's associated-frailty.

[1] D J Mason et al. Mechanically regulated expression of a neural glutamate transporter in bone: a role for excitatory amino acids as osteotropic agents? Bone. 1997 Mar;20(3):199-205.

### P48

### Oral Bisphosphonate Initiation and Adherence after Fragility Fracture: Insights from a Fracture Liaison Service with Digital Medication Monitoring

Maria Talla[1], Craig Cooke[2], Ana Rankovic[2], Andrew Conkie[2]

[1]NHS Greater Glasgow & Clyde, Glasgow, UK. [2]Red Star, Glasgow, UK

### Abstract

**Background**

Fracture Liaison Services (FLS) identify people after a fracture and initiate treatment. Oral bisphosphonates are the most common type of treatment prescribed, but many patients do not start or stick to their treatment, leading to preventable fractures. FLS standards recommend that a medication review is carried out at 16 and 52 weeks following initiation of oral bisphosphonates. We sought to demonstrate the utility of technology to improve adherence.

**Methods**

We reviewed data from Red Star FLS between 1.1.23 to 31.12.23 of those enrolled into FLS within NHS GGC South Sector with a new fragility fracture. Those who were recommended a DXA scan were excluded from analysis as these patients are seen by an Osteoporosis Specialist Nurse (OSN) through a distinct pathway.

When an oral bisphosphonate is recommended via an electronically transmitted letter to the GP, this triggers a virtual medication review at 16 weeks. The review process involves checking the Emergency Care Summary (ECS), a national system providing information on prescribed medications.

Red Star FLS also receives information from NHS GGC Safe Haven allowing identification of patients who are continuing to collect their prescription.

**Results**

343 patients were recommended an oral bisphosphonate and had a subsequent a virtual medication review. 69% of the reviews were carried out within 16 weeks of the initial treatment recommendation.

At 16 weeks, 154/343 (45%) had an oral bisphosphonate prescribed on ECS. Of these 154 patients, 106 (69%) had dispensing entries indicating that they had picked up the prescription from the pharmacy in the preceding 16 weeks. At 52 weeks follow up, 85 of the 154 patients had collected 6 or more prescriptions from the pharmacy in the 52 week period.

116/343 (34%) patients who were recommended an oral bisphosphonate by FLS were never initiated on treatment.

1/343 patients was not started on treatment due to side effects.

72/343 (21%) patients were coded on the medication review as having an '*other*' outcome with associated narrative comments by the OSN.

**Conclusions**

Only 85/343 (25%) of patients were initiated on and continuing to receive an active prescription of oral bisphosphonates at 52 weeks of follow up.

Patients who are discharged to primary care without reassessment are at risk of missing out on treatment which would significantly reduce their risk of a future fragility fracture. Structured, automated digital follow up as provided by the Red Star FLS system would help reduce the harm of avoidable fractures.

### P49

### Prospective Evaluation of Opportunistic Bone Health Assessment Using OsteoSight in Adults Aged ≥50 Undergoing Routine Imaging

Judith Bubbear[1], Isabella Drummond[2], John Skinner[2], Julien M Y Willaime[3], Catherine Kelly[3], Richard Keen[1]

[1]Metabolic Unit, Royal National Orthopaedic Hospital, London, UK. [2]Surgery, Royal National Orthopaedic Hospital, London, UK. [3]Naitive Technologies Limited, Cambridge, UK

### Abstract

### Background

Osteoporosis remains underdiagnosed, with many at risk individuals identified only after a fragility fracture. OsteoSight™, an AI-based image analysis system designed for opportunistic assessment of bone mineral density (BMD) in adults aged ≥50, has previously been evaluated in retrospective datasets comprising individuals who had undergone dual-energy X-ray absorptiometry (DXA) as part of clinical care. However, such populations are inherently enriched for individuals at higher risk of low BMD.

### Aims

The aim of the study was to assess the technical feasibility and preliminary analytical performance of OsteoSight from routine radiographs through a prospective evaluation, in a more representative clinical population.

### Methods

This was a prospective, cross-sectional observational study conducted in an NHS outpatient setting. Adults aged ≥50 undergoing routine hip or pelvic radiography were recruited between July 2025 and February 2026. Radiographs were analysed using OsteoSight™, which incorporates automated checks to determine whether images meet predefined technical criteria before generating an estimated BMD (eBMD) output.

Participants who consented underwent DXA according to the study protocol. This analysis evaluated proportion of radiographs successfully processed, reasons for non-analysable studies and exploratory analytical performance through comparison of AI-derived eBMD with DXA measurements. The study was not powered for definitive diagnostic performance evaluation.

### Results

A total of 50 participants were included in the analysis. The mean age was 67.2 years (range 51–85), and 64% were female. DXA data were available for all participants.

OsteoSight generated an eBMD output in 42 (84%) cases. The rest of the participants had no radiographs analysable, with reasons including incorrect radiographic view and positioning limitations.

The ability of OsteoSight to discriminate low BMD, defined as femoral neck T-score < −1.0, yielded an AUROC of 0.825 (95% CI: 0.691-0.959) with Sensitivity of 0.273 (95% CI: 0.132-0.482) and Specificity of 0.950 (95% CI: 0.764-0.991). eBMD values were higher in male (median=0.694, interquartile range (IQR) = 0.633–0.732) than in female participants (median=0.616, IQR=0.570-0.706), although this was not statistically significant.

Participant recruitment, DXA acquisition, and linkage of radiographic, OsteoSight eBMD predictions, and DXA data were achieved according to the study protocol, with no safety concerns reported.

### Conclusions

Findings from this small prospective study demonstrate that opportunistic AI-based bone health assessment is technically workable in a broader clinical population and shows encouraging early analytical performance relative to DXA. These results extend prior retrospective evaluations of OsteoSight and provide important evidence to inform the design of subsequent, appropriately powered clinical evaluation studies.

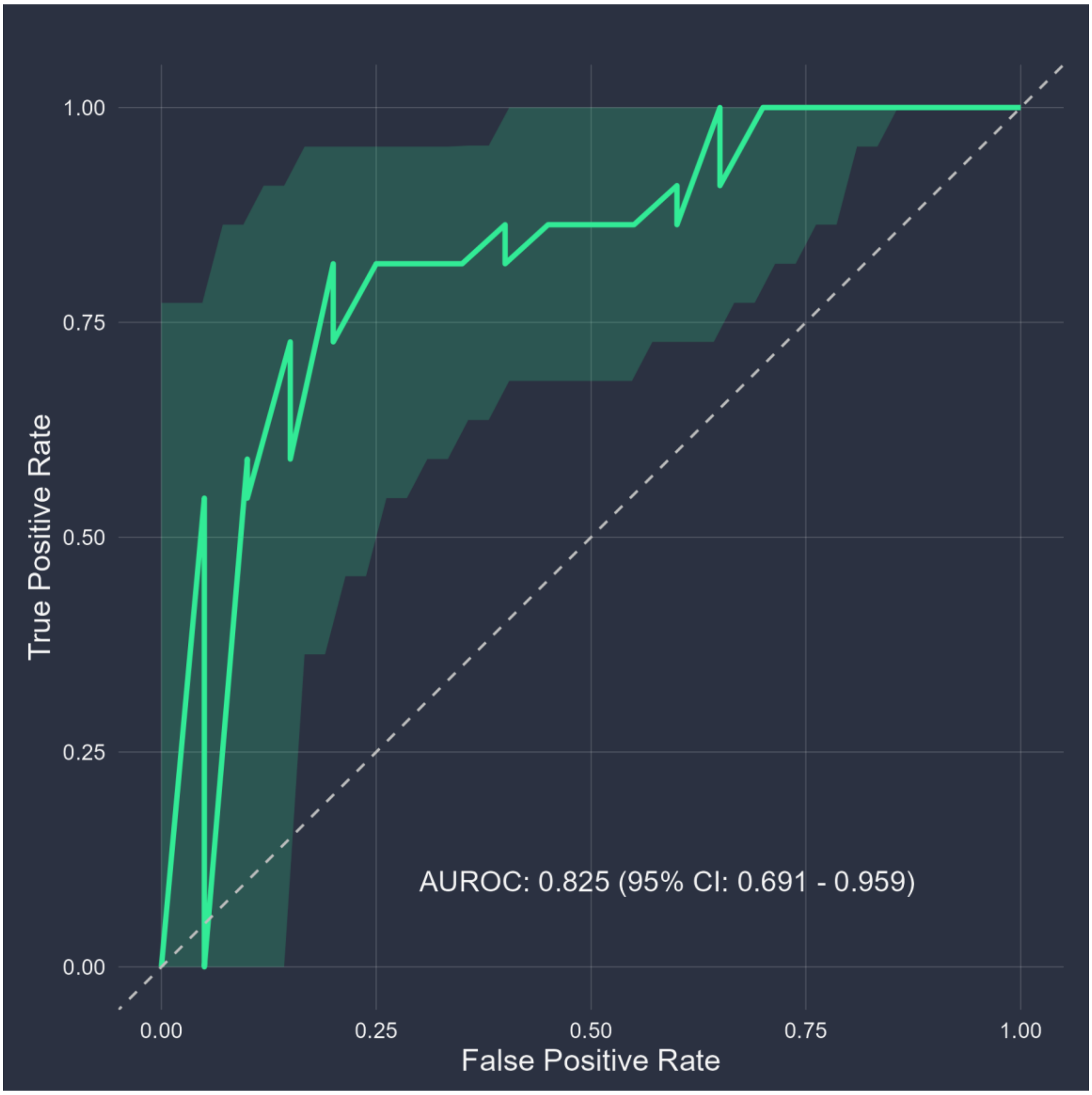

**Figure: Receiver Operating Curve and associated AUROC with 95% confidence intervals for the radiograph-based AI algorithm.**

### P50

### Experience with Anabolic Therapies (Teriparatide and Romosozumab) in Patients with Osteoporosis

Zakaria Rashid, Kapil Kumar Garg, Anupama Nandagudi

Basildon University Hospital, London, UK

### Abstract

INTRODUCTION

Teriparatide and romosozumab are approved anabolic therapies for the treatment of osteoporosis and are recommended for limited-duration use in patients at very high fracture risk, typically followed by antiresorptive therapy to maintain bone mineral density (BMD). While clinical trials demonstrate their efficacy, real-world data on treatment patterns, tolerability, and skeletal outcomes remain limited.

OBJECTIVE

To evaluate the clinical characteristics, treatment patterns, tolerability, and available bone mineral density outcomes in patients receiving teriparatide or romosozumab for osteoporosis at our centre.

METHODS

We performed a retrospective audit of patients receiving teriparatide or romosozumab for osteoporosis at our centre. Demographic data, fracture history, prior osteoporosis therapy, treatment line, adverse effects, and available DEXA results were collected. Patients with documented initiation of anabolic therapy were included. Where paired pre- and post-treatment DEXA scans were available, changes in T-scores were analysed.

RESULTS

Ninety-seven patient records were reviewed, of which 72 patients had documented anabolic therapy initiation: 48 received teriparatide and 24 romosozumab. The mean age at treatment initiation was 69.9 years for teriparatide and 68.6 years for romosozumab. Among patients with recorded sex, 95.7% were postmenopausal women. Anabolic therapy was most commonly used as second-line treatment (62.5% for teriparatide and 58.3% for romosozumab). Prior fragility fracture was documented in 88.9% of patients, most frequently vertebral fractures.

Adverse effects were reported in 37.5% of teriparatide patients and 16.7% of romosozumab patients, though most were mild. Three patients in each treatment group had documented early discontinuation.

Assessment of BMD response to teriparatide was limited by incomplete availability of paired post-treatment DEXA scans, and statistical significance could not be reliably assessed. In contrast, romosozumab demonstrated statistically significant improvements in BMD at multiple skeletal sites, with mean T-score improvements from −2.95 to −2.25 at the lumbar spine ($p<0.001$), −2.78 to −2.60 at the neck of femur ($p=0.038$), and −2.34 to −2.10 at the total hip ($p=0.005$).

CONCLUSION

In this real-world cohort, both teriparatide and romosozumab were generally well tolerated and were most frequently used as second-line therapies in patients with severe osteoporosis and prior fragility fractures. Significant improvements in BMD were observed with romosozumab, while evaluation of treatment response to teriparatide was limited by incomplete follow-up imaging. Continued follow-up will help clarify the comparative effectiveness and optimal sequencing of anabolic therapies in routine clinical practice.

### P51

### Romosozumab Impact on Bone Density and Cardiovascular Risk: A Retrospective Review of the First Cohorts Initiated on Romosozumab at a DGH in England

James Henderson, Khin Yein

Great Western Hospital, Swindon, UK

### Abstract

### Introduction

Romosozumab is a novel monoclonal antibody, anti-osteoporotic medication that has been licensed for use in the UK since 2022. Romosozumab provides an alternative treatment mechanism which can be complimentary to the existing options used in the treatment of osteoporosis, increasing bone mineral density in postmenopausal women. Initial studies have suggested a relative increased cardiovascular risk relating to use of Romosozumab, cardiovascular risk stratification is therefore crucial upon initiation.

### Aim

This review set out to retroactively observe trends in the treatment course of the first cohorts of patients started on Romosozumab in a district general hospital in the Southwest of England. Focussing on changes to bone mineral density following the treatment course, prior therapy, calculated cardiovascular risk (QRISK3) and cardio/cerebrovascular events experienced by the patient population initiated on this medication.

### Method

Data was collected using available patient documentation systems available at Great Western Hospital including digitised clinical letters, inpatient notation, digitised test results, GP records and documentation from other hospitals if that data was available. Data collected included Dexa scan T scores pre/post treatment initiation, time of initiation/cessation, duration of course, specific hospital events and side effects attributed to Romosozumab. If QRISK3 score was not documented GP records and data was used to extrapolate and calculate the score.

### Results

(N=46) Mean age at initiation 67.7 years. Completion of course: 71.4% of patients completed the course, with 11.9% of patients stopping prematurely and 16.7% of patients are still receiving the infusion. Of the 5 patients who did not complete the course, one stopped from side effects related to the medication, with two stopping after 4 months, or not initiating, due to anxiety regarding cardiovascular risk, one being lost to follow-up and one passing away due to an unrelated co-morbidity. Average QRISK3 10-year risk score of patients being initiated on Romosozumab was 13.67% (RR 1.6) (range 2.6-35.2%). Three potential cerebrovascular events were reported, two of which were chronic infarcts without disabling symptoms found following the course of Romosozumab. The other being a TIA that occurred following the initial dose of Romosozumab. There were no recorded cardiovascular events. The other side effects reported were site reactions (n=4), fatigue (n=2), MSK aches (n=4) and headaches (n=2).

The mean improvement of BMD measured on DEXA of the patients that completed the course of Romosozumab was 5.42% (95% CI: 2.72 to 8.12%) to total hip and 14.84% (95%CI 10.96 – 18.70%) to vertebral body.

### P52

### Comparative Mineralisation Phenotyping Across Human and Murine Osteoblast Models to Support Target Discovery in Osteoporosis

Jodie Birch, Goran Tomic, Torsten Gross, Cameron Field, Eugenia Sergeev, Sabine Reichert, Matthew Page, David Budd

Relation Therapeutics, London, UK

### Abstract

Post-menopausal osteoporosis is characterised by impaired bone formation and increased fracture risk resulting from dysregulated bone remodelling. Despite available therapies, there remains a need to identify novel targets with the aim of restoring osteoblast function and promoting bone formation. Relation integrates human genetic data, machine learning and functional screening platforms to identify and validate such targets for osteoporosis. We have developed a mineralisation phenotyping workflow to support early target validation within Relation's osteoporosis discovery programme. Osteogenic differentiation and mineralisation were assessed in primary human osteoblasts (HOBs) from multiple donors and in the murine pre-osteoblast cell line MC3T3-E1. Mineral deposition was quantified using complementary endpoints including calcein incorporation, OsteoImage™ hydroxyapatite detection, and Alizarin Red staining. These orthogonal assays enable comparative evaluation of mineralisation dynamics across species and donor backgrounds while providing robust readouts of osteoblast maturation and extracellular matrix mineralisation. Platform prioritised targets were interrogated using CRISPR-based genetic perturbation strategies in osteoblast models to assess their functional impact on differentiation and mineralisation phenotypes. Subsequent small-molecule profiling was performed to explore pharmacological tractability and pathway modulation. Together, this workflow establishes reproducible mineralisation assays across human and murine osteoblast systems while enabling functional interrogation of genetically informed targets. These approaches provide a scalable platform for prioritising novel regulators of osteoblast biology and advancing therapeutic discovery for post-menopausal osteoporosis.

### P53

Abstract Withdrawn

### P54

### Associations Between Age, Locomotor Stage and Size-Independent Characteristics of Multiple Bones Across Infancy

Charlotte Henderson[1], Antony Colombo[2], Alex Ireland[3]

[1]Independent Researcher, Newcastle, UK. [2]Université Bordeaux Montaigne, Bordeaux, France. [3]Manchester Metropolitan University, Manchester, UK

### Abstract

Little is known about locomotory development – a crucial life stage - in historical populations. Recent work has shown that the introduction of large skeletal loads at onset of walking is associated with large increases in bone strength, but concurrent large body size changes make studies challenging. We aimed to assess associations between age, locomotor stage and size-independent bone geometry markers across upper and lower limb bones.

We assessed 37 skeletons (estimated age-at-death 1.8±1.3y) from the Black Gate cemetery osteoarchaeological collection ($7^{th}$-$12^{th}$ century AD, Northeast England). Peripheral quantitative computed tomography (pQCT) scans of the femur, tibia and radius were taken at 5% increments along each bone from 15% to 85% distal-proximal length. Size-independent indices of bone circularity, relative bone cortical thickness (cortical area divided by total bone area) and relative bone width (total bone area divided by bone length) were calculated. Associations between age, locomotor stage and these indices were assessed via loess regression and multiple linear regression.

Circularity decreased with age in the femur and tibia (both $P < 0.05$) but not radius. Relative bone thickness initially decreased in tibia and femur, but values increased after break-points in the tibia (0.75±0.24y) and femur (1.47±0.36y). In all bones, relative bone width was larger at locomotor stage 2 (6-12 months, standing and crawling) than other stages.

Results showed bone-specific associations between age, locomotor stage and size-independent cortical bone characteristics across infancy in the lower but not upper limb bones, supportive of mediation by loading during locomotion. Further studies in contemporary populations are required to validate these indices as potential biomarkers of locomotor skill acquisition.

### P55

### Development of an In Vitro Bone Model for High-Throughput Screening of Bone Anabolic Compounds

Karthik Vaidya[1,2], Joseph Weightman[2,1], Mohamed Patel[3], Pola Oppenheimer[3], Liam Grover[2,3], Amy Naylor[1]

[1]Department of Inflammation and Aging, University of Birmingham, Birmingham, UK. [2]Healthcare Technologies Institute, University of Birmingham, Birmingham, UK. [3]School of Chemical Engineering, University of Birmingham, Birmingham, UK

### Abstract

### Abstract

Bone remodelling is governed by coordinated interactions between osteoblasts, osteoclasts, and osteocytes within a complex three-dimensional extracellular matrix. Although widely used, many existing in vitro bone models, particularly 2D cultures, fail to replicate the physiological bone microenvironment and often lack functional osteocytes, the primary regulators of bone remodelling in vivo. While several advanced 3D models have been developed, many remain difficult to scale for high-throughput applications required for drug discovery. The Self-Structuring Bone Model (SSBM)[1,2], developed at the University of Birmingham, supports long-term culture of osteoblasts within an organotypic, mineralised extracellular matrix, enabling their differentiation into osteocyte-like cells. Here, we adapted and miniaturised the SSBM for compatibility with high-throughput screening platforms for the identification of novel bone anabolic therapeutics.

Humanised SSBM constructs were miniaturised from the original 6-well plate format to 96- and 384-well plate formats. Constructs were generated using fused deposition modelling (FDM) 3D-printed scaffolds using Bonlecule filament (Ossfila Technology Limited, Hong Kong) as a source of calcium and phosphate, and human adipose-derived stem cells (hADSCs) embedded within a fibrin hydrogel, under osteogenic differentiation media conditions. Protein expression was analysed using enzyme-linked immunosorbent assay (ELISA) of culture supernatants and immunofluorescence (IF) staining to assess the presence of markers associated with mature osteoblasts, and osteocytes, e.g., ALP, P1NP, OPG, PDPN, DMP1. Mineral content was analysed using Raman spectroscopy.

Miniaturised SSBM constructs were successfully generated in both 96- and 384-well formats while maintaining structural integrity and supporting cell viability during culture. Osteoblast and early osteocyte lineage markers increased over the 12-week culture period, indicating successful progression of osteogenic differentiation of hADSCs within the constructs. Raman spectroscopy analysis detected mineral signatures consistent with bone-like mineral deposition, supporting progression of a mineralised extracellular matrix within the model over time.

Overall, this study demonstrates the successful miniaturisation of the humanised SSBM using hADSCs into formats compatible with high-throughput screening. The model maintains key biological characteristics of bone tissue, including osteogenic differentiation and matrix mineralisation, while offering improved scalability. The miniaturised SSBM therefore represents a promising physiologically relevant in vitro platform for automated drug screening and the discovery of novel anabolic therapies targeting bone remodelling.

**Keywords:** 3D in vitro bone model, self-structuring bone model (SSBM), osteoblasts, osteocytes, high-throughput, miniaturisation

### References

1. Finlay M. et al., 2024. DOI: 10.12688/f1000research.130779.3
2. Iordachescu A. et al., 2018. DOI: 10.1002/adbi.201700156

**Miniaturisation of self-structuring bone model (SSBM)**

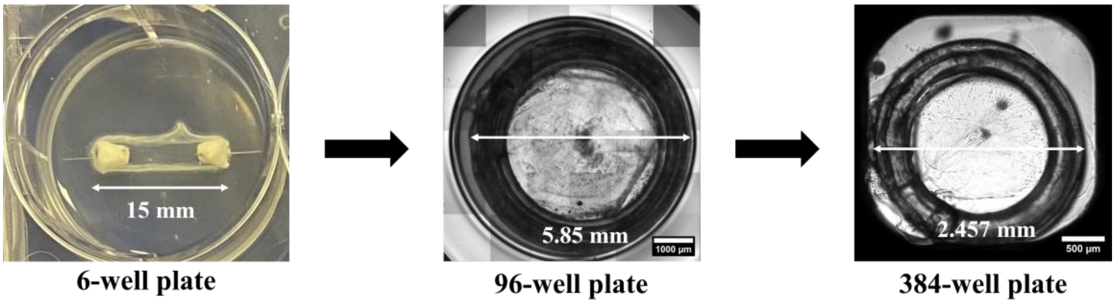

### P56

Abstract Withdrawn

### P57

### Ablation of Dopamine D1 Receptor-Expressing Cells Impairs Alveolar Bone Repair in a Murine Model

Ana Lilia Garcia-Hernandez[1], Andros Balam Islas-Garcia[1], Saul Ernesto Cifuentes-Mendiola[2], Jahaziel Eloy García-Gama[3], Patricia González-Alva[4], Arnulfo Martínez-Dávalos[5], Isaac Obed Pérez-Martínez[6]

[1]Section of Osteoimmunology and Oral Immunology. FES Iztacala, National Autonomous University of Mexico (UNAM), México, Mexico. [2]Section of Osteoimmunology and Oral Immunology. FES Iztacala, National Autonomous University of Mexico (UNAM), Mexico, Mexico. [3]Laboratory of Dental Research, Section of Osteoimmunology and Oral Immunology. FES Iztacala, National Autonomous University of Mexico (UNAM), México, Mexico. [4]4. Laboratory of Tissue Bioengineering, Faculty of Dentistry, National Autonomous University of Mexico, Mexico, Mexico. [5]5. Physics Institute, National Autonomous University of Mexico, México, Mexico. [6]3. Laboratory of Dental Research, Neurobiology of Oral Sensations and Movements Section, FES Iztacala, National Autonomous University of Mexico (UNAM), México, Mexico

### Abstract

Dopamine Type 1 Receptors (D1R) are important regulators of bone formation and resorption and are known to protect against glucocorticoid-induced bone loss by promoting osteogenic differentiation. However, their role in alveolar bone healing after tooth extraction remains unexplored. This study aimed to evaluate the contribution of D1R-expressing cells to post-extraction bone formation using a murine model.

Male C57BL/6 wild-type and D1-Cre transgenic mice (30 weeks old) were divided into four groups (n=6/group): intact wild-type controls, wild-type with extraction (Ext), D1-Cre extraction treated with PBS (D1-Ext), and D1-Cre extraction treated with an adenoviral vector (pAAV-flex-taCasp3-TEVp), inducing selective ablation of D1R-positive cells (D1-Ext-AAV). The first maxillary molar extraction was performed under general anesthesia. The vector or vehicle was administered at the gingival margin 7 days before bilateral maxillary first molar extraction under general anesthesia. We analyzed alveolar bone microarchitecture using micro-CT. We calculated the bone formation rate using intraperitoneal administration of alizarine and calcein for 5 days and observed it using fluorescence microscopy. We analyzed collagen fibers in the socket following socket extraction using Sirius red staining 7 days post-extraction.

Micro-CT analysis revealed that D1-Cre-extraction mice did not differ in bone formation within the extraction sockets compared with wild-type counterparts. Ablation of D1R-positive cells led to a significant reduction in alveolar bone surface area (p=0.0148) and a widened, more open alveolar socket; However, bone volume fraction (BV/TV), trabecular thickness, and separation were not significantly altered compared to D1-Cre-extraction without viral infection.

We observed an increased the new bone formation rate following extraction, especially in D1-Cre extraction mice (p< 0.0001 for both markers), compared with wild-type counterparts. Importantly, selective depletion of D1R-expressing cells caused a marked decrease in fluorochrome incorporation, indicating impaired bone formation in the alveolar socket compared to D1-Cre-extraction without viral infection (p=0.001 alizarine; p=0.002 calcein).

Histologically, extraction sockets in D1-Cre mice revealed more vital bone and a well-organized collagen matrix compared to wild-type extraction sockets. Conversely, D1-Ext-AAV mice showed delayed healing, characterized by a predominance of loose connective tissue, immature collagen fibers, larger empty alveoli, and hypertrophic calcified cartilage zones, suggesting altered ossification pathways.

D1R-expressing periodontal cells are essential for timely alveolar bone repair post-extraction, likely by promoting osteoblast differentiation and extracellular matrix maturation. The absence of these cells delays bone healing and reduces new bone formation, highlighting the crucial role of dopamine signaling in alveolar bone biology.

### P58

### Advancing the Paradigm of PTH Treatment: PTH Rejuvenates the Coupling Mechanism, Inducing the Transition from Eroded to Formative Bone Surfaces in Hypoparathyroidism

Lisbeth Koch Thomsen[1], Galina Pronina[1], Niyoshia Ghalibaf[2], Tanja Sikjaer[3,4], Bente Langdahl[4,5], Christina Møller Andreasen[1,6], Jesper Skovhus Thomsen[5], Lars Rejnmark[4,5], Thomas Levin Andersen[1,6,5]

[1]University of Southern Denmark, Odense, Denmark. [2]University of Southern Den, Odense, Denmark. [3]Gødstrup Regional Hospital, Herning, Denmark. [4]Aarhus University Hospital, Aarhus, Denmark. [5]Aarhus University, Aarhus, Denmark. [6]Odense University Hospital, Odense, Denmark

### Abstract

Previously, it was thought that rapid increases in bone formation induced by daily parathyroid hormone (PTH) injections were mediated by modeling-based bone formation. However, we have recently demonstrated that PTH treatment mainly induce osteoclast-initiated bone formation modes in hypoparathyroidism. Therefore, a new explanation for the reported rapid increase in PTH-induced bone formation is required. Previous studies have linked aging- and osteoporosis-induced bone loss to an inadequate transition from erosion to formation, suggesting an impaired coupling mechanism in these conditions. Here, we investigate whether PTH treatment can rejuvenate the coupling mechanism, thereby improve transition from eroded to formative surfaces, and increase the activation frequency of bone formation.

We applied histomorphometric analyses to trabecular bone surfaces in Masson Goldner trichrome-stained sections from 48 iliac crest biopsies previously obtained in a clinical trial, where patients with hypoparathyroidism (n=48) were randomized to receive either placebo (PLB; n=24) or 100 µg rhPTH(1-84) (PTH; n=24) once daily as add-on to conventional therapy for 6 months. We determined classical histomorphometric parameters and whether eroded bone surfaces were adjacent to formative surface (active eroded) or not (arrested eroded) (fig 1).

We identified a low activation frequency of bone formation in conventionally treated hypoparathyroidism (0.02/year ± 0.04), resulting in an accumulation of arrested eroded trabecular bone surfaces without adjacent osteoid-covered bone surfaces (48% ± 10%) – more than 10-fold higher than previously reported. In the PTH-treated group, eroded surfaces were reactivated and rapidly transitioned into bone forming surfaces, while simultaneously generating new coupled bone remodeling events. This lowered the proportion of eroded surfaces from 55% ± 10% to 31% ± 17% ($p<0.001$) while the proportion of osteoid-covered surfaces rose from 1% ± 2% to 33% ± 21% ($p<0.001$), thereby reducing the ratio of eroded and bone forming surfaces ($p<0.001$). The activation frequency of bone formation was 15-fold higher in the PTH-group (0.31/year ± 0.30) compared with the PLB-group ($p<0.001$). Similarly, PTH treatment resulted in a 16-fold increase in the ratio of active to arrested eroded surfaces ($p<0.001$). Bone formation activity parameters (formation period, wall thickness and mineral apposition rate) were unchanged by PTH treatment. Supplementary active vitamin D-dosage correlated negatively with osteoid surfaces and positively with eroded surfaces and arrested eroded surfaces during PTH treatment.

Collectively, in patients with hypoparathyroidism, PTH treatment increase the initiation of bone formation by reactivating previously accumulated arrested eroded surfaces and rejuvenating the transition from eroded to bone forming surfaces, thereby restoring the coupling mechanism

Fig. 1 - Bone remodeling during hypoparathyroidism and PTH treated hypoparathyroidism

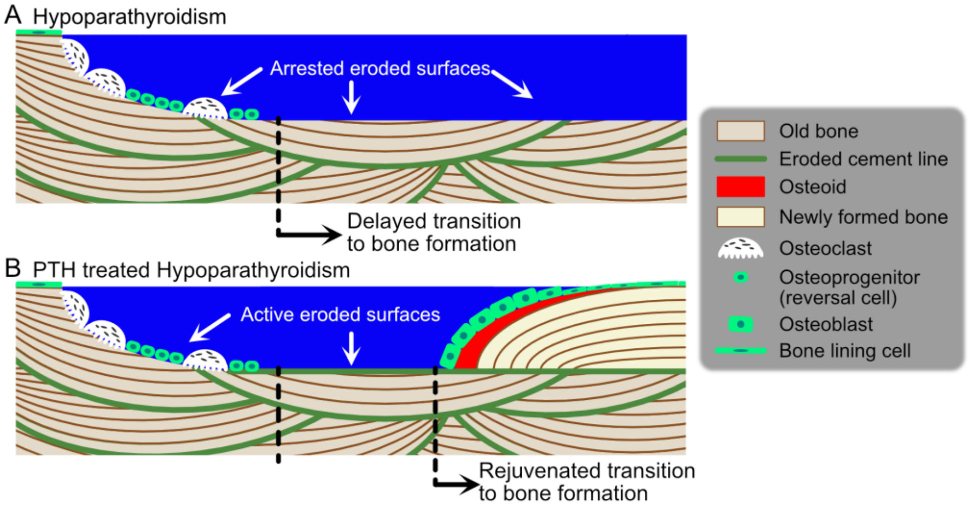

Figure 1 - Schematics illustrating (A) arrested eroded surfaces - eroded surfaces with no adjacent formative surfaces - in conventionally/placebo treated hypoparathyroidism. (B) In PTH treatment eroded surfaces are reactived to active eroded surfaces - eroded surfaces with adjacent formative surfaces - through a rejuvenation of the coupling mechanism ensuring transition to bone formation.

### P59

### Growth Outcomes and Safety of Navepegritide in Children with Achondroplasia: Results of the ApproaCH Trial Open Label Extension

Emma Webb[1], Ravi Savarirayan[2,3,4], Carlos Bacino[5], Ciara McDonnell[6,7], Hanna Hove[8], Philippe Campeau[9], Paul Hofman[10], Janet Legare[11], Daniel Hoernschemeyer[12], Josep Maria de Bergua Domingo[13], Jennifer Abuzzahab[14], Bart Degreve[15], Lærke Clement Freiberg[15], Helle Hartvig[15], Michael Ominsky[16], Allison Komirenko[16], Aimee Shu[16], Atiya Kenworthy[17]

[1]Jenny Lind Children's Hospital, Norfolk and Norwich University Hospital NHS Foundation Trust, Norwich, UK. [2]Murdoch Children's Research institute, Parkville, Australia. [3]Royal Children's Hospital, Parkville, Australia. [4]University of Melbourne, Parkville, Australia. [5]Baylor College of Medicine, Houston, USA. [6]Children's Health Ireland at Temple Street, Dublin, Ireland. [7]University of Dublin, Trinity College, Dublin, Ireland. [8]Copenhagen University Hospital, Rigshospitalet, Copenhagen, Denmark. [9]CHU Sainte-Justine Research Center, Montreal, Canada. [10]The Liggins Institute, University of Auckland, Auckland, New Zealand. [11]University of Wisconsin School of Medicine and Public Health, Madison, USA. [12]University of Missouri Children's Hospital, Columbia, USA. [13]Unidad de Cirugia Artroscopica, Vithas Vitoria Hospital, Vitoria-Gasteiz, Spain. [14]Children's Minnesota, Minneapolis, USA. [15]Ascendis Pharma A/S, Hellerup, Denmark. [16]Ascendis Pharma Inc., Palo Alto, USA. [17]Ascendis Pharma, London, UK

### Abstract

**Introduction:** Navepegritide, an investigational prodrug of C-type natriuretic peptide (CNP) administered once-weekly, is designed to provide continuous exposure to active CNP to counteract the constitutively active fibroblast growth factor receptor-3 in achondroplasia.

**Methods:** The pivotal, double-blind, placebo-controlled ApproaCH Trial randomized 84 children aged 2-11 with achondroplasia to navepegritide (100 µg/kg/week; n=57) or placebo (n=27) for 52 weeks, followed by a 52-week open-label extension (OLE) in which all participants received navepegritide. Week-104 growth and safety outcomes are reported.

**Results:** Navepegritide demonstrated superiority over placebo for annualized growth velocity (AGV) at 52 weeks (least-squares mean difference: 1.49 cm/year; p<0.0001). Children continuing navepegritide treatment (n=55) maintained elevated observed mean AGV from Week 52 (5.95 cm/year) through Week 104 (5.65 cm/year); mean AGV at Week 104 was 5.42 cm/year in children switching from placebo to navepegritide (n=27). In both groups, achondroplasia-specific height Z-score improved during the OLE (+0.27 and +0.28, respectively), and improvement was seen in upper-to-lower body segment ratio.

In the OLE, most adverse events (AEs) were mild or moderate, with no treatment-related serious AEs, deaths, nor AEs that led to trial discontinuation. The rate of injection site reactions was 0.31 events per person-year of exposure (all mild). No participants experienced symptomatic hypotension.

**Discussion:** During the ApproaCH Trial OLE, children continuing treatment with navepegritide maintained increased AGV, and children who switched from placebo to navepegritide at Week 52 demonstrated increased AGV at Week 104. Improvements in achondroplasia-specific height Z-score were consistent with navepegritide-related improvements observed in the double-blind period. Navepegritide was generally well-tolerated.

### P60

### Prevalence of Hypophosphatasia in Rheumatology and Osteoporosis Care Clinics: A Systematic Literature Review

Valentin S Schäfer[1], Katie Moss[2], Carolina Tornero[3], Julia F Charles[4], Jonathan D Adachi[5], Andrea J Singer[6], Jessica Hinman[7], Liliana-Georgiana Wegmann[8], Shona Fang[9]

[1]Department of Rheumatology and Clinical Immunology, Clinic of Internal Medicine III, University Hospital Bonn, Bonn, Bonn, UK. [2]St. George's Hospital, London, UK. [3]Hospital Universitario La Paz, Madrid, Spain. [4]Brigham and Women's Hospital and Harvard Medical School, Boston MA, USA. [5]McMaster University, Hamilton, ON, Canada. [6]MedStar Georgetown University Hospital, Washington DC, USA. [7]Epi Excellence, LLC, Philadelphia, PA, USA. [8]Alexion, AstraZeneca Rare Disease, Baar, Switzerland. [9]Alexion, AstraZeneca Rare Disease, Boston MA, USA

### Abstract

**Background:** Hypophosphatasia (HPP) is a rare, inherited metabolic disease caused by deficient tissue-nonspecific alkaline phosphatase (ALP) activity. Because of its heterogeneous clinical presentation, HPP can be misdiagnosed as osteoporosis, fibromyalgia, or a form of arthritis, among other disorders.

We sought to describe the prevalence of HPP in rheumatology and osteoporosis clinics.

**Methods:** A systematic review of full-length studies published in English between 2015 and 2024 was conducted using PubMed and search terms related to HPP Data were extracted on study characteristics, number and percentage of individuals with persistently low ALP activity (generally ≥2–3 measures below normal), and number of patients identified with HPP. The prevalence of HPP in the study population, including individuals with persistently low ALP activity, is reported.

**Results:** Of 331 publications identified, 59 met criteria for full-text review and 28 were analyzed for data extraction. Of the 28 publications, 3 reported studies in patients treated in a rheumatology setting and 2 in patients in an osteoporosis setting. The remaining 23 studies were within settings other than rheumatology and osteoporosis and are not presented here. One additional German rheumatology study with English abstract was included. Overall, the percentage of outpatients with persistently low ALP activity ranged from 0.38% (7/1839) to 1.22% (28/2289). In 2 studies in rheumatology outpatients, the prevalence of genetically confirmed HPP among those with persistently low ALP was 46.4% (13/28) and 56.5% (13/23). One study of patients with fibromyalgia and ALP activity below the upper limit of normal reported that 9.3% (57/611) had persistently low ALP, but this did not exclude known secondary causes of low ALP and there was no further evaluation to confirm HPP. Among rheumatology and internal medicine in patients with persistently low ALP activity, 6.0% (11/182) were genetically confirmed to have HPP. Among patients seen at an osteoporosis clinic who had persistently low ALP, prevalence of genetically confirmed HPP was 57.1% (4/7). In another study in patients seen at a UK osteoporosis clinic, 87.5% (14/16) of those with persistently low ALP had potentially pathogenic *ALPL* variants, although specific signs and symptoms of HPP were not stated.

**Conclusion:** The prevalence of HPP appears to be high in rheumatology and osteoporosis clinics among adults with persistently low ALP activity where at least 46.4% to 57.1% had genetically confirmed HPP. These findings should be a call to action for practitioners in rheumatology and osteoporosis clinics to test for ALP and emphasize the importance of investigating low ALP activity, as HPP is highly prevalent among patients with persistently low ALP.

### P61

### Bone Mineral Density in Adults with Hypophosphatasia with or without History of Fractures

Lothar Seefried[1], Priya S Kishnani[2], Kathryn M Dahir[3], Cheryl Rockman-Greenberg[4], Keiichi Ozono[5], Gabriel A Martos Moreno[6], Wolfgang Högler[7], Agnès Linglart[8], Anna Petryk[9], Shona Fang[9], Peter R Ebeling[10]

[1]University of Würzburg, Würzburg, UK. [2]Duke University Medical Center, Durham, NC, USA. [3]Vanderbilt University Medical Center, Nashville, TN, USA. [4]University of Manitoba, Winnipeg, Manitoba, Canada. [5]ISEIKAI International General Hospital, Osaka, Japan. [6]Hospital Infantil Universitario Niño Jesús, IIS La Princesa, Universidad Autónoma de Madrid, CIBERobn, ISCIII, Madrid, Spain. [7]Johannes Kepler University Linz, Linz, Austria. [8]Paris-Saclay University, AP-HP and INSERM, Paris, France. [9]Alexion, AstraZeneca Rare Disease, Boston, MA, USA. [10]School of Clinical Sciences, Monash University, Clayton, VIC, Australia

### Abstract

**Purpose:** To describe the prevalence of bone mineral density (BMD) T-scores ≤−2.5 in adults with hypophosphatasia (HPP) with and without history of fracture (HoF), stratified by age.

**Methods:** Fractures were analyzed in asfotase alfa-naive adults enrolled in the Global HPP Registry (data cut: June 2023). Patients were stratified into younger adults (aged 18–≤50 y) and older adults (aged >50 y). Dual X-ray absorptiometry (DXA) results within 12 months of fracture (or most recent DXA for patients without HoF) were deemed evaluable. Lumbar spine, hip neck, and total hip were measured. Osteoporosis was indicated by BMD T-scores ≤−2.5. BMD T-scores, difference in medians (DM), and 95% confidence intervals are presented.

**Results:** The analysis included 278 younger adults (106 with HoF) and 249 older adults (142 with HoF). In younger adults, 17 with HoF and 86 without HoF had an evaluable DXA. Median age at DXA was 33.9 y in those with HoF and 37.9 y in those without HoF. Median T-scores were within the normal range in younger adults but were lower at all sites measured for those with vs those without HoF (**Table**). The proportions of younger adults with HoF who had T-scores ≤−2.5 were 8% (1/13), 17% (2/12), and 9% (1/11) at the lumbar spine, hip neck and total hip, respectively. No younger adults without HoF had T-scores ≤−2.5. In older adults, 16 with HoF and 56 without HoF had an evaluable DXA. Median age at DXA was similar in those with vs without HoF (60.6 vs 60.8 y). Median T-scores were within normal range in older adults and were similar between those with and without HoF at all sites measured (**Table**). The proportions of patients with T-scores ≤−2.5 were similar between those with vs without HoF (lumbar spine: 4/16 [25%] vs 11/51 [22%]; hip neck: 3/16 [19%] vs 13/44 [30%]; total hip: 3/13 [23%] vs 3/38 [8%]). Metatarsal/femoral fractures characteristic of HPP were present in 10/17 (59%) younger adults and 9/16 (56%) older adults with HoF; fractures characteristic of osteoporosis/low BMD (eg. vertebra, hip, wrist, humerus) were present in 5/17 (29%) younger adults and 7/16 (44%) older adults with HoF.

**Conclusions:** Median BMD T-scores were within a normal range in adults regardless of fracture history. Some patients may have concomitant osteoporosis in addition to the underlying HPP-related osteomalacia. Further studies are warranted to differentiate between osteoporotic fractures and those due to compromised mineralization.

| T scores, median (range) | Lumbar spine | Hip neck | Total hip |
|---|---|---|---|
| **Younger adults (18–≤50 y)** | | | |
| With HoF | n=13<br>**−1.2** (−2.8, 1.2) | n=12<br>**−1.4** (−2.6, 0.6) | n=11<br>**−1.3** (−2.5, 0.4) |
| Without HoF | n=75<br>**0** (−1.9, 2.3) | n=61<br>**−0.4** (−2.4, 1.4); | n=53<br>**−0.3** (−2.2, 1.7) |
| DM,<br>95% CI | −1.1<br>−1.9, −0.4 | −0.8<br>−1.4, −0.1 | −0.9<br>−1.5, −0.3 |
| **Older adults (>50 y)** | | | |
| With HoF | n=16<br>**−0.8** (−4.1, 3.8) | n=16<br>**−1.5** (−3.7, −0.3) | n=13<br>**−1.6** (−2.9, 0.5); |
| Without HoF | n=51<br>**−0.8** (−3.8, 5.4) | n=44<br>**−1.4** (−3.5, 0.7) | n=38<br>**−1.1** (−3.4, 1.7) |
| DM,<br>95% CI | −0.3<br>−1.4, 0.9 | 0<br>−0.6, 0.7 | −0.8<br>−1.5, 0.1 |

### P62

### Real-World Experience of Burosumab in Adults with X-Linked Hypophosphataemia (XLH) Aged Over 65 Years in the UK

Judith Bubbear[1], Elaine Murphy[2], Gavin Clunie[3], Matthew Roy[4], Lak Kotinkaduwa[5], Ben Johnson[6], Gillian Logan[6], Daniel Stevens[7], Richard Keen[1]

[1]Royal National Orthopaedic Hospital, Stanmore, UK. [2]University College London Hospitals NHS Foundation Trust, London, UK. [3]Cambridge University Hospitals NHS Foundation Trust, Cambridge, UK. [4]University Hospitals Bristol NHS Foundation Trust, Bristole, UK. [5]Kyowa Kirin, Princeton, USA. [6]Kyowa Kirin, Marlow, UK. [7]Bionical Emas, Willington, UK

### Abstract

**Background/Introduction:** XLH is a rare, genetic, progressive, phosphate-wasting disorder that causes skeletal morbidities, stiffness, pain and impaired physical function throughout life. Burosumab inhibits fibroblast growth factor 23, increasing renal phosphate reabsorption. The phase 3 trial of burosumab excluded patients aged >65 years. To address this evidence gap, we explored the experience of burosumab in older patients in a real-world setting through a UK early access programme (EAP). Results for the overall adult EAP cohort have been reported previously.

**Purpose:** To describe the baseline population and clinical characteristics, burosumab dosing and outcomes in burosumab-naïve adults aged >65 years. Outcomes included numerical change from baseline in serum phosphate concentration, Brief Pain Inventory Short Form (BPI-SF), Western Ontario and McMaster Universities Arthritis Index (WOMAC) and EuroQol 5-dimension 5-level (EQ-5D-5L) scores at the most recent assessment for each patient.

**Results:** Data are available for 10 patients: mean (SD) age 70.7 (5.23) years; 80% female; 80% white; mean (SD) body mass index 31.0 (6.48) kg/m$^2$ (n=7). Three were receiving oral phosphate and/or active vitamin D immediately before starting burosumab treatment; 9 were taking pain medication (4 opioids). Mean (SD) burosumab dose at baseline was 63.0 (12.52) mg. All patients had serum phosphate concentrations below the lower limit of normal (LLN) at baseline; 90% had levels exceeding the LLN at their most recent assessment (median (IQR) duration of follow-up, 277 (182, 407) days). Serum phosphate concentration increased from baseline in all patients. Mean (SD) concentration increased from 0.61 (0.098) mmol/L at baseline to 1.01 (0.166) mmol/L at the most recent assessment; paired mean change (SD) from baseline was 0.39 (0.137) mmol/L (n=10). Mean (SD) WOMAC scores improved numerically from baseline across all domains (Stiffness, −8.3 (21.89); Pain, −2.5 (18.64); Physical Function, −14.0 (17.86)), as did the total score (−11.1 (16.27)) (n=6). Mean (SD) BPI-SF scores also improved numerically (Worst Pain −1.0 (2.45); Pain severity −1.0 (1.74); Pain interference −0.5 (1.25); n=6). Mean (SD) improvement in EQ-5D-5L visual analogue scale was 6.3 (19.31) (n=4) and in the index score was 0.06 (0.122) (n=7). Three subjects discontinued treatment during the EAP (1 injection site reaction, 1 lack of perceived benefit, 1 not detailed). Median (IQR) time to discontinuation was 0.3 (0.2, 0.7) years.

**Conclusions:** The numerical improvements from baseline in serum phosphate concentration and WOMAC, BPI-SF and EQ-5D-5L scores in these older adults are consistent with findings in the overall adult EAP cohort treated with burosumab.

### P63

### Echocardiography Screening and Follow-Up in Osteogenesis Imperfecta: 114 Patient Case Series

Jennifer Walsh[1,2], Oscar Dawson[1], Syazrah Salam[1], Nigel Wheeldon[1]

[1]Sheffield Teaching Hospitals, Sheffield, UK. [2]University of Sheffield, Sheffield, UK

### Abstract

It has been proposed that the collagen defect in osteogenesis imperfecta can cause cardiac abnormalities, including valve dysfunction and aortic dilatation. Sheffield Metabolic Bone clinic has offered routine echocardiography screening for adults with OI since 2008. This service evaluation was approved by Sheffield Teaching Hospitals Governance. Patients were all under the care of Sheffield Metabolic Bone clinic, but echocardiography was done at their local hospital according to local echo protocol. Therefore there was some variability in the parameters and format reported.

Echocardiography was done for 114 patients between 2008 and 2026. The mean age at first echo was 32 years (range 15-73); 52 were male and 62 were female. The range of OI severity was: mild 40, moderate 63, severe 11.

Abnormalities were reported on echo in 46 patients (some patients had more than one reported abnormality).

| Reported abnormality | Age, years mean (range) | M/F N | OI mild/mod/sev N |
|---|---|---|---|
| Valve regurgitation n=26<br><br>Aortic: mild 2, mod 1<br>Mitral: mild 10, mod 1<br>Pulmonary: mild 10<br>Tricupsid: mild 8, mod 1 | 41 (19-73) | 17/9 | 15/7/4 |
| Ventricular dysfunction n=11<br><br>Left: mild 6<br>Right: mild 2, mod 1 | 34 (20-65) | 4/5 | 4/5/0 |
| Aortic dimensions n=13<br><br>Raw values only: 4<br>Body surface area indexed only: 3<br>Raw and indexed: 6 | 46 (19-73) | 8/5 | 8/3/2 |

Twenty patients had routine repeat echo, 2 to 9 years from first scan (average 6 years). Only one had a new abnormality reported at repeat imaging (mild aortic and mitral regurgitation.)

Nearly all patients could be adequately assessed with echocardiography. Only two patients with severe OI needed MRI to obtain clear views. The most appropriate method to index aortic measurements to body size in OI is not known.

In conclusion, echocardiographic abnormalities are probably more common in patients with OI than in the general population, and are reported across all ages and severity of OI. Most reported abnormalities are mild.

### LB01

Abstract Withdrawn

### LB02

### Non-Invasive Measurement of Osteoporotic Bone Loss in Mice after Corticosteroid Treatment of Ovariectomy using High Resolution MicroCT

Jen-Chieh Tseng, Alexis Stanley, Julie Braza, Craig McMannus, Jack Sharkey, Jeffrey Peterson

Revvity, Waltham, USA

### Abstract

Osteoporosis (OP) is both a chronic and acute bone condition that impacts bone mass and density. Growth plate thickness and trabecular volume (TV) are key metrics in OP. While microCT is a key preclinical imaging modality for bone measurements, these assessments are often carried out at end point, requiring large mouse numbers.

Here, we monitored TV and growth plate thickness in two models of bone loss: chronic, ovariectomy-induced (OVEX) OP, and acute PRED associated OP. Both models were assessed at different timepoints using the Quantum GX3 microCT (Revvity, Inc.) to image and measure bone loss in the spine and femurs. High resolution images were captured both in living animals and post-mortem.

Chronic OP; SKH-1E female mice (n=3 per timepoint) were ovariectomized at 4 weeks of age, mice were imaged in vivo at 7, 8, 9, 11 and 13 weeks old. Femurs and spines were collected after each timepoint for ex vivo imaging. Acute OP; BALB/c female mice (n=3 per timepoint) received PRED (10 mg/kg, bid) for two weeks (saline control), then imaged. For in vivo imaging, both femurs and lumbar spine were scanned separately [4 mins, 80 kV (160 µA), FOV 36 mm, 0.5 mm Al filter]. Bone microCT images were acquired for high resolution ex vivo validation [4 mins, 80 kV (50 µA), FOV 8 mm, 0.5 mm Al filter]. The microCT images were then subject to bone microarchitecture analysis (BMA) using Analyze 15 software.

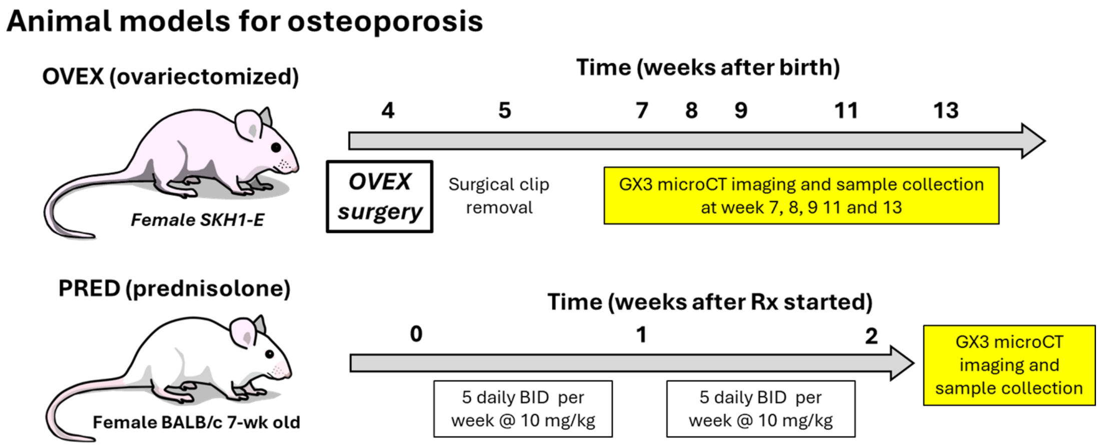

BMA analyses of OVEX microCT images shows loss of bone density and TV. Bone density reduction was seen in in vivo (FOV36) scans of the spine cortex and femur trabeculae as early as 9 weeks. TV suppression can also be observed throughout the course of imaging. High-resolution ex vivo (FOV8) scans confirmed in vivo findings and provide higher resolution volumetric assessments of OVEX spines. Similarly, two weeks of short-term PRED treatment is sufficient to cause loss in both bone density (-6% in cortex and -9% in intertrabecular) and volume (-15% TV), as shown by in vivo and ex vivo scans. The OVEX and PRED models differed significantly in the magnitude of TV loss, with dramatic loss seen in OVEX mice and mild or little trabecular bone loss seen with PRED. Assisted by high-resolution ex vivo imaging and quantification, the pixel size improved 4.4-fold to 2.86 µm. Thus, the Quantum GX3 allows assessment of the fine structural details within trabecular bone and growth plate regions, confirming non-invasive observations, and offering enhanced quantification and visualization capabilities.

FOV36 (in vivo)
FOV8 ( ex vivo)
Ctrl
PRED
Ctrl
PRED

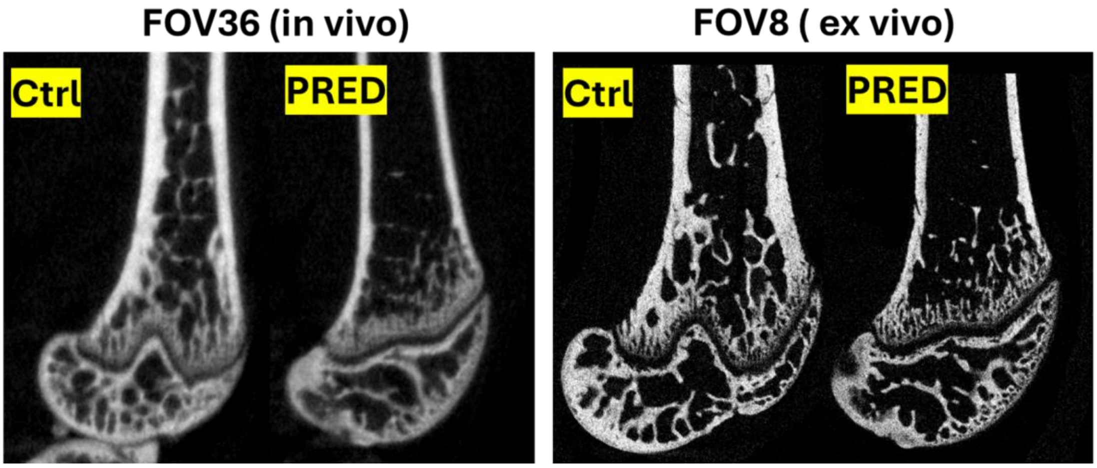

### LB03

### Agrin Overexpression Enhances Osteoblastic and Impairs Adipocyte Differentiation of Mesenchymal Stem Cells

Letícia Faustino Adolpho[1], Maria Paula Oliveira Gomes[1], Gileade Pereira Freitas[2], Helena Bacha Lopes[2], Adalberto Luiz Rosa[1], Márcio Mateus Beloti[1]

[1]School of Dentistry of Ribeirão Preto - University of São Paulo, Ribeirão Preto, Brazil. [2]School of Dentistry - Federal University of Goiás, Goiânia, Brazil

### Abstract

Exogenous agrin (AGRN) discretely increases osteoblastic differentiation of mesenchymal stem cells (MSCs). Considering that osteoblastic and adipogenic differentiation are opposite processes, we hypothesized that agrin overexpression in MSCs is effective in favouring osteoblastic and impairing adipogenic differentiation of MSCs. To test this hypothesis, MSCs derived from the adipose tissue of C57Bl/6 male mice were immortalized and genetically edited using CRISPR-Cas9 to overexpress AGRN. Subsequently, they were cultivated in osteogenic or adipogenic media for up to 17 days. The gene expression of *Agrn* and its receptors *Lrp4* and *Dag1* was assessed by qRT-PCR at 3, 5, 7, 10 and 14 days (n=4, ANOVA, p≤0,05). Osteoblastic differentiation was assessed by gene expression of *Runx2*, *Alp*, *Col1a1,* and *Bglap* (n=4, ANOVA, p≤0,05) at 3, 5, 7, 10 and 14 days, protein expression of RUNX2 at 5 days, *in situ* ALP activity at 7 days, and extracellular matrix mineralization at 17 days (n=5, Student's t-test, p≤0,05). Adipogenic differentiation was assessed by gene expression of *Pparγ*, *Adipoq*, and *Retn* (n=4, ANOVA, p≤0,05) at 3, 5, 7, 10 and 14 days, protein expression of PPARγ at 5 days, and lipid accumulation at 17 days (n=5, Student's t-test, p≤0,05). During osteoblastic differentiation, AGRN overexpression increased *Agrn*, *Runx2*, *Alp*, *Col2a1* and *Bglap* gene expression, RUNX2 protein expression, *in situ* ALP activity and extracellular matrix mineralization and reduced *Lrp4* and *Dag1* gene expression. During adipogenic differentiation, AGRN overexpression increased *Agrn* and *Dag1* gene expression, delayed *Pparγ* and *Adipoq* gene expression, and reduced *Retn* gene expression as well as PPARγ protein expression. However, *Lrp4* gene expression and lipid accumulation were not significantly affected. These results suggest that agrin may be a promising therapeutic target for inhibiting the augmentation of bone marrow adiposity in certain diseases, such as osteoporosis. Furthermore, its potential to favour osteogenesis suggests that agrin can contribute to the development of new therapeutic approaches for bone regeneration.

### LB04

### Understanding the Mechanisms of Pain and Tissue Damage Induced by Bone Marrow Lesions in Osteoarthritis

Andisheh Niakan[1], Joseph Westaby[1], Anissa Chikh[1], Franklyn Howe[1], Sulaiman Alazzawi[2], Suroosh Madanipour[2], Philip Mitchell[2], Nidhi Sofat[1,3]

[1]City St George's, University of London, School of Health and Medical Sciences, London, UK. [2]St George's University Hospitals NHS Foundation Trust, Department of Orthopaedics, London, UK. [3]St George's University Hospitals NHS Foundation Trust, Department of Rheumatology, London, UK

### Abstract

Introduction:

Osteoarthritis (OA) is the most prevalent form of arthritis worldwide and is a leading cause of chronic pain and disability. OA affects the whole joint, including cartilage, subchondral bone, and synovium. Bone marrow lesions (BMLs) are early biomarkers of joint damage in OA and are associated with structural changes, including loss of osteochondral integrity, new blood vessel and nerve formation, fibrosis, cysts, and de novo cartilage formation, as defined by the OsteoArthritis Bone Score (OABS) (1). Our group previously performed a whole transcriptomic analysis of OA-BML regions, which showed upregulation of genes involved in neurogenesis, angiogenesis, pain sensitisation, inflammation, and cartilage remodelling pathways, including stathmin 2 (STMN2), thrombospondin 4 (THBS4), and matrix metalloproteinase 13 (MMP13) (2).

Aim:

In this study, we aimed to investigate the mechanisms and functions of STMN2, THBS4, and MMP13 in the pathogenesis of OA pain and disease progression.

Methods:

Human tissues were collected with full informed consent from participants undergoing knee arthroplasty with full Ethical Approval (UK REC number 99426). Immunocytochemistry, confocal microscopy, and qPCR were used to assess the expression profiles of STMN2, THBS4, and MMP13 in MG-63 osteoblastic and C20A4 chondrocyte cell lines stimulated with IL-1β and TNF-α. In addition, the expression, localisation, and co-localisation of STMN2, THBS4, and MMP13 were studied by immunofluorescence and confocal microscopy in BML tissues from participants with OA.

Results:

Our results showed a significant increase in the expression of STMN2, THBS4, and MMP13 in MG-63 and C20A4 cells stimulated with cytokines compared to unstimulated cells. Confocal microscopy results showed localisation of these proteins in the cytoplasm, nucleus, and cell membrane of MG-63 and C20A4 cells. Our analysis of human tissue revealed STMN2, THBS4, and MMP13 expression in OA-BML tissue sections, which was absent in non-OA controls. The strongest expression was observed in articular cartilage and OA-BML subchondral bone, with the most significant expression within BMLs in blood vessels, inflammatory and fibrotic regions (see Figure showing confocal microscopy of OA-BML with THBS4, STMN2, DAPI [A] and THBS4, MMP13, DAPI [B]).

Conclusion:

Our data show that STMN2, THBS4, and MMP13 produced in OA-BML are upregulated in response to inflammatory cytokines in cell models and are also overexpressed in articular cartilage and in OA-BML subchondral bone, particularly in blood vessels and in inflammatory and fibrotic regions.

Figure

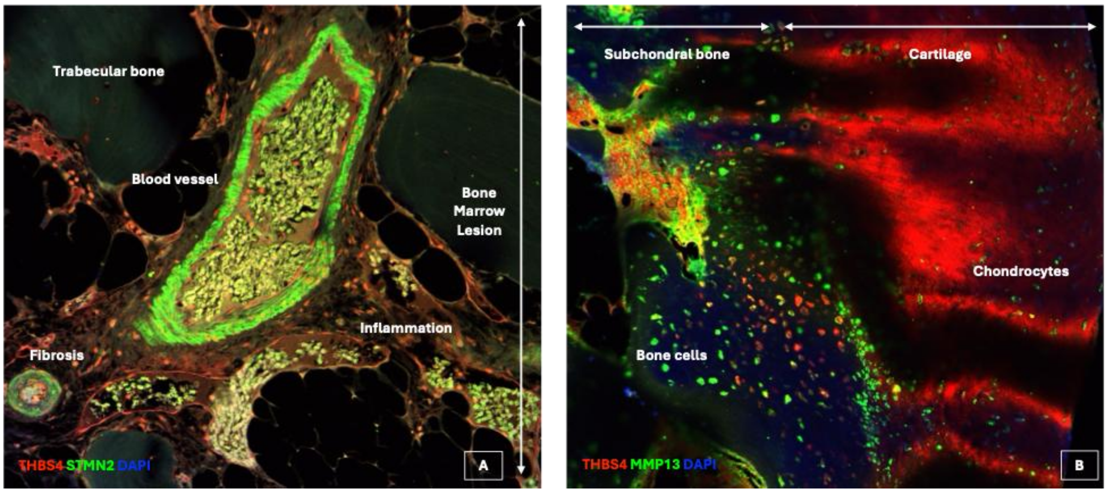

References:

1. Koushesh S, et al. Osteoarthritis Cartilage 2022;30:746-55.

2. Kuttapitiya, A, et al. Annals Rheumatic Diseases 2017; 76(10): 1764-1773.

LB05

### A 3D Bioengineered *in vitro* model to Study Primary Cilia as Mechanochemical Integrators in Bone Development

Kateřina Apolínová[1], Swati Midha[1,2,3], Amélie Jule[3], Théana Johnson[3], Clarissa Coveney[3,4], Jadwiga Zarebska[3], James Armstrong[5], Stephen Sansom[3], Tonia Vincent[3], Angus Wann[1,3]

[1]University of Southampton, Southampton, UK. [2]Queen Mary University of London, London, UK. [3]The Kennedy Institute of Rheumatology, University of Oxford, Oxford, UK. [4]Harvard University, Cambridge, Massachusetts, USA. [5]University of Bristol, Bristol, UK

### Abstract

During adolescence, rapid longitudinal bone growth is driven by endochondral ossification (EO) in the growth plate (GP), a highly orchestrated process regulated by an interplay of biological morphogens and mechanical cues. Our previous *in vivo* work in mouse GPs has revealed that Intraflagellar Transport 88 (Ift88), a gene essential for the assembly of primary cilia, is necessary for correct GP development. The conditional knockout of Ift88 in mice using the Tamoxifen-inducible Cre-Lox system causes delays in bone formation and significant GP abnormalities. Paradoxically, after limb off-loading through surgical immobilisation, the Ift88cKO phenotype is rescued, suggesting that Ift88 or the primary cilium protects the GP from extrinsic mechanical forces. However, the molecular mechanisms underpinning the role of the primary cilium as an integrator of mechanical and chemical cues remain poorly understood.

To characterise the transcriptomic changes induced by changes to mechanical loading and/or Ift88cKO, we performed bulk RNA sequencing on adolescent mouse GPs. The results revealed that 20% of the WT GP transcriptome (4060 genes) was significantly altered by limb off-loading alone (Fig A), further demonstrating that the GP is a remarkably mechano-sensitive tissue. Weighted Gene Co-expression Network Analysis (WGCNA) identified ciliary genes as a central mechanosensory hub in response to limb off-loading (Fig B), further cementing the key role of the primary cilium as a sensor and effector of mechanical cues during EO. Remarkably, off-loading also caused a downregulation of Ift88 expression itself in wild-type mice (Fig C), revealing that Ift88 is both regulated by, and itself regulates, biological responses to changes in mechanical loading during EO.

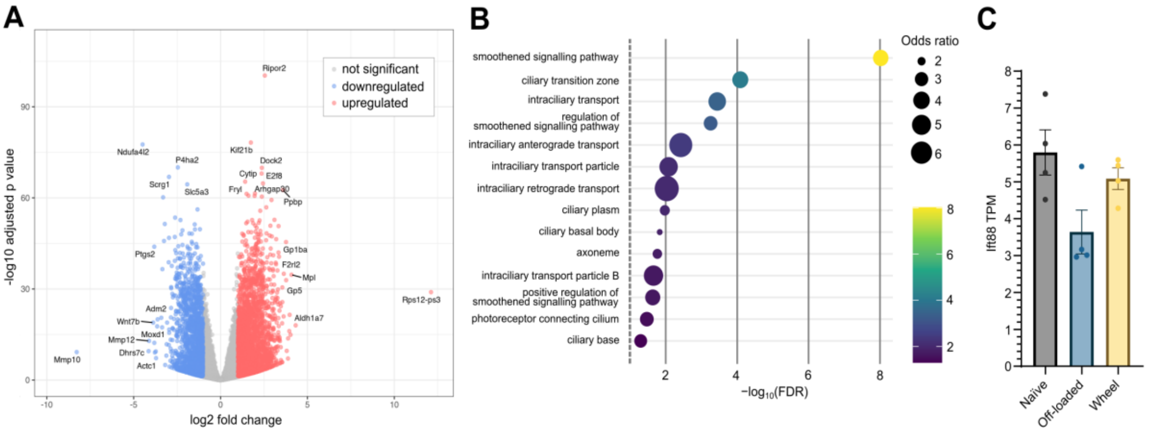

However, differential gene expression analysis also revealed that tamoxifen administration independently alters GP transcriptomics, confounding our ability to disentangle the effects of Ift88cKO from tamoxifen-induced changes. To bypass this limitation and dissect the mechanochemical interactions in a translationally exploitable setting, we move towards an *in vitro,* 3D bioengineered model of EO mechanobiology. In this setup, bone marrow-derived human mesenchymal stem cells (hMSCs) are encapsulated in gelatin methacrylate (GelMA) hydrogels of tightly controlled mechanical properties, containing a defined gradient of bone morphogenetic protein (BMP). This 28-day culture system simulates longitudinal chondro-osseous transitions, and allows for the application of controlled mechanical compression. In combination with CRISPR/Cas9-driven gene knockout, this platform will enable us to probe how the primary cilium integrates chemical and mechanical signals during the complex differentiation processes underlying EO.

### LB06

### Zebrafish *ngfb* Mutants Recapitulate Rapidly Progressive OA and Reveal Essential Roles for NGF in Joint Homeostasis

Dalia Bugshan

Institute of Genetics and Cancer, Edinburgh, UK

### Abstract

**Zebrafish *ngfb* mutants recapitulate rapidly progressive OA and reveal essential roles for NGF in joint homeostasis.**

**Dalia Bugshan1 , Beatriz Larraz Prieto1 , Jiun Kai Ng1 , Erika Kague1 1 Institute of Genetics and Cancer, University of Edinburgh, UK**

Osteoarthritis (OA) is a leading global cause of pain and disability worldwide, yet no disease-modifying therapies currently exist. While anti-Nerve Growth Factor (NGF) therapies effectively alleviate OA pain, clinical trials were halted due to incidences of rapidly progressive OA (RPOA). This suggests NGF plays critical roles in joint integrity beyond mere nociception. Understanding NGF's function in joint homeostasis and RPOA is therefore essential for developing safer therapeutics.

Because NGF knockout mice die in early development, elucidating NGF's function in skeletal biology has been severely limited. To overcome this, we generate *ngfb*$^{-}$/$^{-}$ zebrafish mutants that survive to adulthood, enabling a comprehensive investigation of NGF function in joint and skeletal maintenance.

Although initial joint development appeared normal, Alcian Blue staining revealed craniofacial cartilage abnormalities in 5dpf larvae, including increased Meckel's and ceratohyal widths. These structural changes were accompanied by functional deficits in jaw movement, with *ngfb*$^{-}$/$^{-}$ larvae exhibiting reduced jaw movement frequency and altered jaw dynamics compared to wild-type, suggesting an overall impact on joint function. Furthermore, immunostaining against acetylated tubulin revealed reduced neural input within craniofacial regions, corroborating disrupted neuroskeletal interactions during development.

In adults, skeletal phenotyping via Alizarin Red staining, micro-computed tomography (µCT), and Tartrate-Resistant Acid Phosphatase (TRAP) staining revealed consistent abnormalities. These included abnormal vertebral arch mineralisation, reduced bone mineral density, ectopic intervertebral mineralisation, and increased osteoclast activity. Remarkably, *ngfb*-/- jaw joint exhibited sclerosis and space narrowing reminiscent of OA. Most importantly, a subset of mutants showed spontaneous joint collapse, recapitulating clinical RPOA.

Together, our findings demonstrate that *ngfb* has a multifaceted role in regulating joint health, craniofacial cartilage morphology, neuronal innervation, and bone homeostasis. Our work establishes *ngfb*-/- zebrafish as a powerful in vivo model to dissect NGF's function in skeletal biology and RPOA pathogenesis, providing important mechanistic insights into the clinical failure of anti-NGF therapies.

### LB07

### Vigorous Activity in Midlife is Associated with Lower Risk of Hip Fracture: Findings from a Prospective Study in UK Biobank

Catherine Rolls[1,2,3], Benjamin G Faber[2,3], Emma M Clark[2], Marcus Munafó[4], Helen Dawes[5], Jonathan H Tobias[2]

[1]Translational and Applied Research Group, University of Bristol, UK, Bristol, UK. [2]Musculoskeletal Research Unit, University of Bristol, Bristol, UK. [3]Integrative Epidemiology Unit, University of Bristol, Bristol, UK. [4]4. Vice Chancellor's Office, University of Bath, Bath, UK. [5]3. NIHR Exeter, Biomedical Research Centre, Medical School, University of Exeter, Exeter, UK

### Abstract

**Background:** Hip fractures are a major cause of morbidity, mortality, and healthcare utilisation in older adults. In the UK, social care costs within 120 days of hip fracture are estimated at £1.25 billion annually. Physical activity may represent a cost-effective preventive strategy; however, evidence linking mid-life physical activity with later hip fracture risk remains limited, particularly regarding the volume and intensity of activity associated with benefit. This study investigated the association between leisure-time physical activity in mid-life and subsequent risk of hip fracture in UK Biobank (UKB).

**Methods:** We conducted a prospective cohort analysis of middle-aged UKB participants using self-reported leisure-time physical activity data linked to Hospital Episode Statistics records for incident hip fracture over 18 years of follow-up. Total physical activity volume was calculated by combining walking, moderate, and vigorous activity using Metabolic Equivalent Task (MET)-minutes/week. Additional analyses examined vigorous activity separately, including mutually adjusted models for vigorous activity independent of walking and moderate activity. Associations with incident hip fracture were examined using Cox proportional hazards models. Physical activity exposures were categorised into quintiles, with sex-stratified analyses performed due to violation of the proportional hazards assumption for sex.

**Results:** Among 322,749 participants with complete data, 53% were female and the mean age was 47 years. During follow-up, 2,674 incident hip fractures were recorded. Median total leisure-time physical activity increased across quintiles from 100 to 1470 minutes/week in females and from 90 to 1545 minutes/week in males (Table 1). Associations between total physical activity volume and hip fracture were non-linear and differed by sex (Figure 1). In analyses by vigorous activity participation, in females, lower hip fracture risk was observed for Q3 to Q5, the lowest risk being for Q4 (HR 0.79, 95% CI 0.70–0.87), corresponding to approximately 90 (60–120) minutes/week. In males, lower hip fracture risk was observed for Q3 and Q4 of vigorous activity, the lowest risk being for Q3 (HR 0.64, 95% CI 0.52–0.79), corresponding to 30 (20–40) minutes/week. Associations for vigorous activity remained unchanged following adjustment for walking and moderate activity.

**Conclusions:** These findings suggest that moderately increased participation in vigorous physical activity was associated with lower subsequent risk of hip fracture in both sexes. Further analyses are needed to establish whether these findings represent a true benefit of vigorous physical activity or whether participation in vigorous activity acts as a marker of other factors related to frailty and hip fracture risk.

*Table 1 Total leisure time activity composition by quintile and sex*

| Sex | PA quintile (Q) | N | Total activity MET min/week (Median (IQR) | Total activity min/week (Median (IQR) | Walking min/week Median (IQR) | Moderate min/week Median (IQR) | Vigorous min/week Median (IQR) |
|---|---|---|---|---|---|---|---|
| Female | Q1 | 34042 | 346 (198–495) | 100 (50–140) | 60 (30–100) | 10 (0–30) | 0 (0–0) |
| Female | Q2 | 35665 | 978 (812–1158) | 240 (210–300) | 150 (90–210) | 60 (30–105) | 10 (0–40) |
| Female | Q3 | 35247 | 1770 (1538–2024) | 425 (370–500) | 210 (125–360) | 120 (60–180) | 40 (0–90) |
| Female | Q4 | 34830 | 3063 (2639–3546) | 720 (620–870) | 350 (210–525) | 240 (120–360) | 90 (15–160) |
| Female | Q5 | 31915 | 5958 (4932–7758) | 1470 (1215–1890) | 840 (420–1260) | 600 (360–900) | 150 (40–300) |
| Male | Q1 | 30815 | 330 (148–492) | 90 (40–130) | 60 (20–100) | 10 (0–30) | 0 (0–1) |
| Male | Q2 | 29192 | 982 (813–1158) | 240 (205–290) | 150 (80–210) | 60 (20–90) | 15 (0–45) |
| Male | Q3 | 29610 | 1772 (1542–2026) | 420 (360–485) | 210 (120–360) | 120 (60–180) | 60 (0–120) |
| Male | Q4 | 30027 | 3060 (2640–3546) | 705 (595–840) | 315 (175–420) | 210 (120–315) | 120 (30–180) |
| Male | Q5 | 32941 | 6426 (5106–8818) | 1545 (1215–2100) | 840 (420–1260) | 630 (360–900) | 210 (75–380) |

*Figure 1 Physical activity (PA) quintile model and risk of hip fracture*

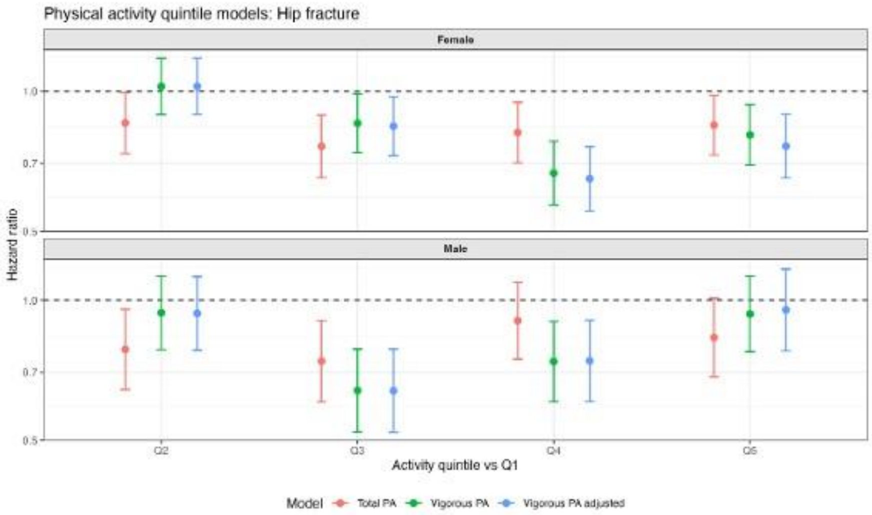

### LB08

### Physical Activity Volume, WHO Guideline Attainment and Fracture Risk in Mid-Life: Findings from UK Biobank

Catherine Rolls[1,2,3], Jonathan H Tobias[2], Emma M Clark[2], Helen Dawes[4], Marcus Munafó[5], Bejamin G Faber[2,3]

[1]Translational and Applied Research Group, University of Bristol, Bristol, UK. [2]Musculoskeletal Research Unit, University of Bristol, Bristol, UK. [3]Integrative Epidemiology Unit, University of Bristol, Bristol, UK. [4]NIHR Exeter, Biomedical Research Centre, Medical School, Exeter, UK. [5]Vice Chancellor's Office, University of Bath, Bath, UK

### Abstract

**Background:**

Concerns regarding fracture risk are known to discourage physical activity participation during mid-life, particularly among women around the menopause. Therefore, women might be failing to get the wider benefits of physical activity. This study aims to investigate associations between physical activity participation and fracture risk in UK Biobank to inform public health messaging.

**Methods:**

We performed a cross-sectional analysis of UK Biobank participants aged 40–65 years using self-reported physical activity and fracture data. Total physical activity volume (minutes/week) was calculated by combining frequency and duration of walking, moderate, and vigorous activity. Associations between total activity volume and fracture risk were initially explored across quintiles. Participants were additionally categorised according to whether they did not meet, met, or exceeded WHO physical activity recommendations (150–300 minutes/week of moderate-equivalent activity), based on weekly activity minutes calculated as walking plus moderate plus (2 × vigorous) activity. Multivariable logistic regression models examined associations with self-reported fracture within the previous five years. Sex-stratified analyses were conducted due to established differences in physical activity and fracture risk.

**Results:**

Among 322,749 participants, 53% were female, the mean age for the cohort was 47 years. 95% of the cohort were of white ethnicity. Fracture within the previous five years was reported by 9.7% of women and 8.9% of men, corresponding to 30,088 self-reported fractures. Physical activity volume increased progressively across quintiles in both sexes. Participants in Q1 accumulated a median of approximately 95–100 WHO-equivalent minutes/week, below recommended activity levels, whereas those in Q4–Q5 accumulated substantially higher activity volumes, exceeding recommended levels (women: 840–1620 minutes/week; men: 830–1740 minutes/week) (Table 1). Physical activity demonstrated a non-linear association with fracture risk. Meeting WHO-recommended activity levels was not associated with increased fracture risk in either women (OR 0.96, 95% CI 0.91–1.02) or men (OR 1.01, 95% CI 0.94–1.08). In contrast, activity volumes exceeding WHO recommendations were modestly associated with increased fracture risk, particularly in men (women: OR 1.06, 95% CI 1.01–1.11; men: OR 1.30, 95% CI 1.23–1.37). Findings were consistent between adjusted and sensitivity analyses.

**Conclusions:**

Physical activity undertaken within WHO-recommended levels was not associated with increased fracture risk in either women or men during mid-life. In contrast, activity volumes exceeding WHO recommendations were associated with a modest increase in fracture risk, particularly among men.

These findings support current public health recommendations encouraging participation in physical activity within recommended guideline levels.

**Table 1:** Activity profiles across physical activity quintiles by sex (Values are median (IQR))

| Sex | PA quintile | N | WHO equivalent min/week of moderate activity* | Walking min/week | Moderate min/week | Vigorous min/week |
|---|---|---|---|---|---|---|
| Female | Q1 | 34042 | 100 (55–140) | 60 (30–100) | 10 (0–30) | 0 (0–0) |
| | Q2 | 35665 | 270 (225–320) | 150 (90–210) | 60 (30–105) | 10 (0–40) |
| | Q3 | 35247 | 480 (420–550) | 210 (125–360) | 120 (60–180) | 40 (0–90) |
| | Q4 | 34830 | 840 (720–960) | 350 (210–525) | 240 (120–360) | 90 (15–160) |
| | Q5 | 31915 | 1620 (1340–2100) | 840 (420–1260) | 600 (360–900) | 150 (40–300) |
| Male | Q1 | 30815 | 95 (45–140) | 60 (20–100) | 10 (0–30) | 0 (0–1) |
| | Q2 | 29192 | 270 (225–320) | 150 (80–210) | 60 (20–90) | 15 (0–45) |
| | Q3 | 29610 | 480 (420–550) | 210 (120–360) | 120 (60–180) | 60 (0–120) |
| | Q4 | 30027 | 830 (720–960) | 315 (175–420) | 210 (120–315) | 120 (30–180) |
| | Q5 | 32941 | 1740 (1380–2370) | 840 (420–1260) | 630 (360–900) | 210 (75–380) |

* WHO equivalent minutes/week moderate activity = sum of moderate + walking + (2 x vigorous) minutes/week

Fig1

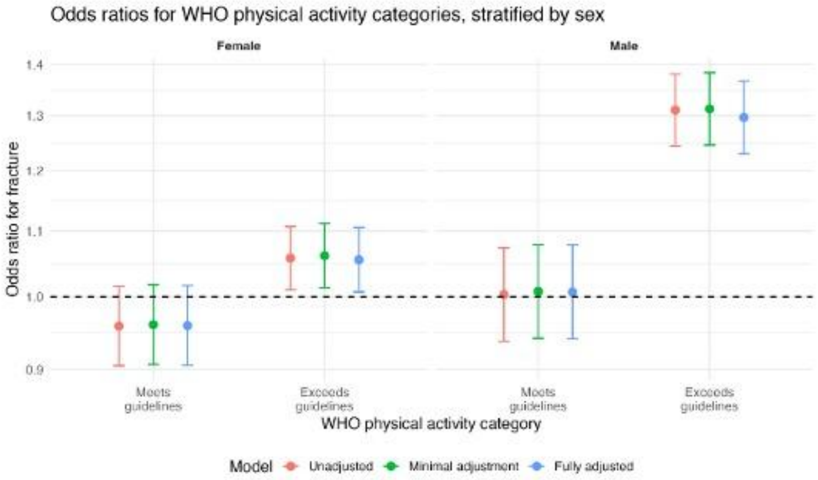

*Adjustment models: (1) unadjusted; (2) minimally adjusted for age, ethnicity, weight and height; and (3) fully adjusted, additionally including socioeconomic deprivation and education.*

### LB09

### Two Modes of Trabecular Volumetric Densification after Exercise

Alan Boyde[1], David Mills[1], Elwyn Firth[2]

[1]QMUL, London, UK. [2]University of Auckland, Auckland, New Zealand

### Abstract

The level of exercise known as cantering induces rapid replacement of fatty marrow with bone in distal regions of equine third metapodial bones. We used archived material from the Massey University Grass Exercise Study (MUGES) to study this phenomenon: seven 2–year–old Thoroughbred fillies trained over 13 weeks, and seven untrained confined to grass yards. Distal metacarpal (Mc3) slices were embedded in PMMA. Micro-milled block surfaces were studied by backscattered electron mode SEM, confocal fluorescence microscopy and – after more subdivision and sectioning – by more SEM, high contrast resolution X–ray MicroTomography (XMT) and transmitted ordinary and polarized light microscopy. Finally, bone tissue was removed by sequential HCl and hypochlorite bleach treatments (with ultrasonication to remove osteocyte casts) to leave the PMMA as a mould of the marrow and blood vessel canal space for 3D SEM (Figure).

Very rapidly generated and densely mineralised strands of ‘woven’ bone formed in fatty marrow space distant from the nearest pre-existing trabecular bone surfaces and any osteocytes: this new phase provides scaffold for lamellar bone deposition in close relationship to and enclosing a dense capillary blood vessel network. The fine–bore blood vessel canals in this rapidly-formed new infill bone connect with those within the pre-existing trabecular bone plates. Additional small blood vessel canals are formed by osteoclastic-team tunnelling, without the repair-refilling phase seen in secondary osteon formation.

New lamellar bone formed on pre–existing trabecular surfaces without prior resorption, had mineralization levels below and autofluorescence levels above those in existing bone and incorporated blood vessel canals perpendicular to the growth surface: capillaries penetrating the osteoblastic carpet as observed at growing endosteal surfaces in small rodent tubular bones.

Both these types of new bone contribute to volumetric densification, although their net fabric density is less than that of preceding bone.

Using correlated findings from several SEM modes, ordinary and polarised light microscopy and XMT (µCT), we demonstrate previously unrecognised morphological types of bone and conclude that osteocytes cannot be involved in signalling to produce the most rapidly produced, highly vascular bone infill.

**Figure caption**. MUGES exercised Mc3 palmar oblique section, lateral condyle, subsample with right–angled edge, before and after removing bone. Top side is medio–lateral, lower is antero–posterior (para–sagittal). Left is distal. 20kV BSE–SEM. Field widths = 4.27mm.

A
B
C
D
E
F
B
A
C
E
F
D

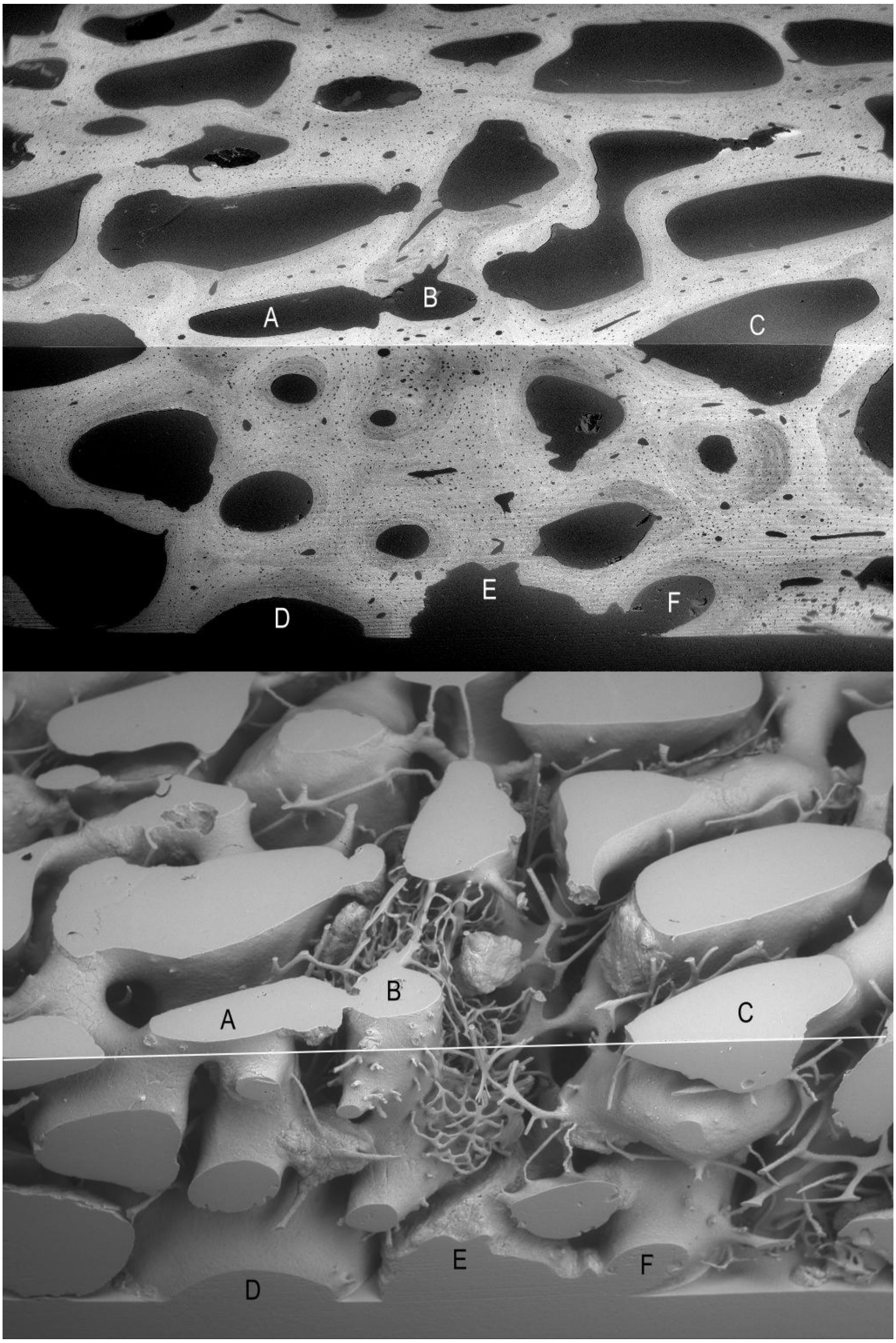

### LB10

### The Role of P2rx7 in the Bone Phenotype of the *Dmd* $^{mdx\text{-}\beta Geo}$ Mouse

Robin Rumney[1], Charles Wood[2], Dariusz Górecki[2]

[1]University of Southampton, Southampton, UK. [2]University of Portsmouth, Portsmouth, UK

### Abstract

Duchenne muscular dystrophy (DMD) is a severe X-linked condition associated with muscle degeneration resulting in severe, life threatening cardiac and respiratory complications in early adulthood. It is noteworthy that DMD is also associated with reduced bone strength and increased skeletal fragility. DMD is caused by mutations in the *DMD* gene that encodes dystrophins. There are multiple dystrophin isoforms from the full length Dp427 down to Dp71, and so the location of the *DMD* mutation will determine which of these isoforms are affected.

Previously we demonstrated that ablation of the P2RX7 purinergic receptor in the *Dmd* $^{mdx}$ mouse that has a point mutation preventing expression of full-length dystrophins only resulted in specific phenotypic alterations. In this study, we characterise the bone morphometry and the impact of P2RX7 ablation in the *Dmd* $^{mdx\text{-}\beta Geo}$ mouse that does not express any dystrophin isoforms and has exacerbated pathology. Tibiae from 8-week-old male C57BL/6, *Dmd* $^{mdx\text{-}\beta Geo}$, *P2rx7*$^{KiKo}$ (cell-specific ablation) and *P2rx7*$^{-/-}$ mice were imaged with high resolution micro-CT (Zeiss Xradia) and data analyzed with CTAN.

Cortical bone volume was decreased 0.7-fold in *Dmd* $^{mdx\text{-}\beta Geo}$ mice compared to C57BL/6 controls while the bone surface/volume (BS/BV) ratio was increased 2.3-fold. The BS/BV ratio was further increased in *Dmd* $^{mdx\text{-}\beta Geo}$ *P2rx7*$^{-/-}$ mice compared to *Dmd* $^{mdx\text{-}\beta Geo}$ alone. In trabecular bone, bone volume/total volume was over 0.9-fold lower in *Dmd* $^{mdx\text{-}\beta Geo}$ mice compared to C57BL/6 controls, while the BS/BV ratio was 7-fold higher. In *Dmd* $^{mdx\text{-}\beta Geo}$ mice trabecular spacing was 1.6-fold higher and trabecular number 0.9-fold lower than C57BL/6 controls.

These data clearly demonstrate the importance of dystrophin isoforms in bone morphology and that P2X7 has a mediating role.

### LB11

### 3D Bone Surface Reconstruction of the Tibia Using Tracked Freehand Ultrasound Sequences and an Image Segmentation Model

Chuin Hong Yap[1], Connah Kendrick[1], Amaka Offiah[2], Alex Ireland[1]

[1]Manchester Metropolitan University, Manchester, UK. [2]University of Sheffield, Sheffield, UK

### Abstract

Ultrasound offers a real-time, cost-effective, radiation-free alternative to traditional bone imaging modalities (i.e. CT, MRI), but current 3D ultrasound techniques are limited by issues such as scanning volume or post-processing workload. This study presents a novel ultrasound probe tracking approach designed to reconstruct the 3D tibial bone surface from freehand ultrasound sequences, utilising an articulated arm to continuously record the probe's spatial position during scanning.

Data were collected from eighty-two adult participants (46% female, mean age 38±9y). The experimental setup incorporated an Acuson P500 ultrasound machine fitted with a linear L10-5v probe and attached to a FARO FaroArm QuantumE 7-axis portable coordinate measuring machine to provide spatial tracking. B-mode ultrasound image sequences were captured simultaneously with the probe's tracked positional data during multiple sweeps of the shank. For the 3D reconstruction, bone surface points were initially extracted by manual annotation of the bone boundary within each 2D ultrasound frame. These 2D points were subsequently projected into 3D space using the tracked positional data alongside a calibrated spatial offset. Annotations were used to train and evaluate image segmentation models, and performance of different architecture/encoder pairings was evaluated.

The 3D reconstruction process was successfully demonstrated in sequential steps. Initially, the continuous trajectory of the tracked arm was plotted in 3D space. Next, the 2D ultrasound images were aligned along the trajectory using the rotational data provided by the articulated arm. Finally, the bone surface annotations were overlaid using the coordinates from each respective ultrasound image as a spatial reference. The resulting reconstruction overlay demonstrated the annotated bone surface (shown in red) properly aligned within the 3D space, while the tracked sweep geometry (shown in blue) confirmed continuous coverage along the intended scan path. Regarding bone surface segmentation, UnetPlusPlus architecture paired with a ResNet34 encoder delivered the highest overall performance achieving an IoU_Image of 0.5604, an IoU_Dataset of 0.5612, and an F1-score of 0.7190.

Combining tracked freehand ultrasound sequences with a 7-axis articulated measurement arm successfully reconstructs a 3D tibial bone surface. The resulting 3D geometry visually corresponds accurately to the frame-level 2D annotations, and the bone segmentation model shows promising results supporting further reconstruction analysis. Future investigations will aim to automate the bone surface extraction process using deep learning-based methods, conducting quantitative accuracy validation of the 3D reconstructions against ground-truth CT or MRI scans, and evaluating the system's ergonomic feasibility in clinical settings.

### LB12

### Spontaneous Femoral Fractures in Two Patients with SGMS2-Related Skeletal Dysplasia

Eleanor Palmer, Kenneth Poole

University of Cambridge, Cambridge, UK

### Abstract

Osteoporosis with calvarial doughnut lesions is a dominant skeletal dysplasia, typically presenting with low bone mineral density, spontaneous fracture, and sclerotic lesions. This condition is thought to be caused by mutations in SGMS2, with one case series outlining six unrelated families with SGMS2-related osteoporosis.

A 47-year-old male with a previous diagnosis of infantile osteoporosis was referred to rheumatology following spontaneous femoral fractures. He has suffered from several atraumatic femoral fractures throughout his lifetime. His most recent fracture of the left femur was a spontaneous mid-femoral tensile fracture. On examination, he was well-built, 1.8 metres tall and weighing 119.2kg. He had right-sided Bell's palsy, normal sclerae and normal scarring. His calcium and phosphate were normal, with a raised ALP. X-Ray of the femora showed bilateral bowing, and intramedullary nail present in the left femur. Bone densitometry revealed femoral neck density of 1.177g/cm3 and L1-3 density of 1.003g/cm3. Genetic studies showed a heterozygous SGMS2 mutation (c.148C>T p.Arg50Ter). Biopsy has been requested but not yet performed.

The son of this patient, a 7-year-old with Down syndrome, presented at day seventeen of life with bilateral femoral fractures and bowing. He has sustained multiple atraumatic fractures throughout his lifetime, including fractures of his ribs, radius, ulna and metatarsals. Recent pelvic radiographs show continued bowing and evidence of Perthes disease affecting the right hip. His genetic studies are in process.

SGMS2 encodes sphingomyelin synthase (SMS2), which catalyses the formation of sphingomyelin, a substrate required for generation of phosphocholine. Phosphocholine is a substrate for PHOSPHO1, which is responsible for phosphate liberation required for bone mineralisation. PHOSPHO1 knockout in mice induces spontaneous fractures, bowing of the long bones, osteomalacia and scoliosis, features consistent with the findings in these cases. Additionally, the mid-femoral shaft stress fractures seen in these cases have phenotypic similarities to those seen in patients with hypophosphatasia (HPP). Fractures in HPP are proposed to occur due to abnormalities in the crystal structure of the bone mineral, rather than osteomalacia, and happen despite normal bone density. Similarly, in the first case, the femoral fracture occurred despite average bone density, in the tensile region of the femoral shaft, perhaps indicating a shared molecular mechanism.

Both patients have been treated high dose vitamin D replacement and received bisphosphonates to treat their skeletal fragility. The role of bisphosphonates in their treatment may need to be reconsidered pending further mechanistic studies into the bone crystal structure in this disease.
